# Two-photon isomerization properties of donor–acceptor Stenhouse adducts†

**DOI:** 10.1039/d3sc01223a

**Published:** 2023-04-24

**Authors:** Francisco A. Reza-González, Emmanuel Villatoro, Mariana M. Reza, Jesús Jara-Cortés, Héctor García-Ortega, Edgard F. Blanco-Acuña, José G. López-Cortés, Nuria Esturau-Escofet, Alan Aguirre-Soto, Jorge Peon

**Affiliations:** a Instituto de Química, Universidad Nacional Autónoma de México Ciudad de México Mexico jpeon@unam.mx; b Unidad Académica de Ciencias Básicas e Ingenierías, Universidad Autónoma de Nayarit Tepic 63155 Mexico; c Facultad de Química, Universidad Nacional Autónoma de México Ciudad de México Mexico; d School of Engineering and Sciences, Tecnologico de Monterrey Monterey Nuevo Leon Mexico

## Abstract

Donor–acceptor Stenhouse adducts (DASAs) are important photo-responsive molecules that undergo electrocyclic reactions after light absorption. From these properties, DASAs have received extensive attention as photo-switches with negative photochromism. Meanwhile, several photochemical applications require isomerization events to take place in highly localized volumes at variable depths. Such focused photoreactions can be achieved if the electronic excitation is induced through a non-linear optical process. In this contribution we describe DASAs substituted with extended donor groups which provide them with significant two-photon absorption properties. We characterized the photo-induced transformation of these DASAs from the open polymethinic form to their cyclopentenic isomer with the use of 800 nm femtosecond pulses. These studies verified that the biphotonic excitation produces equivalent photoreactions as linear absorbance. We also determined these DASAs' two-photon absorption cross sections from measurements of their photoconverted yield after biphotonic excitation. As we show, specific donor sections provide these systems with important biphotonic cross-sections as high as 615 GM units. Such properties make these DASAs among the most non-linearly active photo-switchable molecules. Calculations at the TDDFT level with the optimally tuned range-separated functional OT-CAM-B3LYP, together with quadratic response methods indicate that the non-linear photochemical properties in these molecules involve higher lying electronic states above the first excited singlet. This result is consistent with the observed relation between their two-photon chemistry and the onset of their short wavelength absorption features around 400 nm. This is the first report of the non-linear photochemistry of DASAs. The two-photon isomerization properties of DASAs extend their applications to 3D-photocontrol, non-linear lithography, variable depth birefringence, and localized drug delivery schemes.

## Abstract 2

Photo-switching of Stenhouse donor–acceptor adducts by a non-linear optical input.

## Graphical abstract

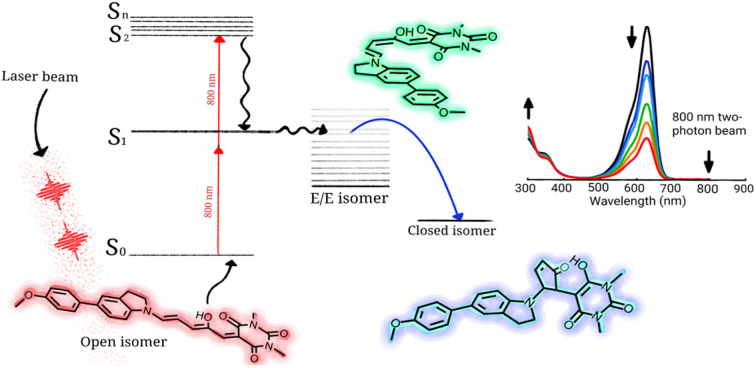

## Introduction

One of the most precise ways to control events at the molecular level is through photochemical reactions. For example, these schemes have made it possible to induce the activity of biological systems like ion-channels and enzymes.^1^ Such approaches have also served to photo-activate polymerization reactions,^2^ and to induce the function of catalysts.^3,4^ In these applications, photoinduced isomerization reactions are among the most successful methods to achieve such goals.^5,6^

Several of the mentioned technologies require actuators that can be photoconverted in a highly localized volume. This includes patterning and 3D printing,^7^ control of detachment events inside cells,^8,9^ and the release of the contents of liposomes.^10^ The option of initiating processes through two-photon absorption allows for micrometric precision through the use of appropriate lens systems, since these processes are intrinsically dependent on the square of the light intensity.^11–14^ Diverse molecular designs have recently been developed in order to combine the two-photon absorption properties of molecules with other aspects of their photophysics.^15–17^

The interest in these photo-actuators has been increasing in recent years as reflected by the development of new molecular switches with significant two-photon absorption cross sections, either through electronic transitions of the actuator itself,^18–20^ or with the use of a molecular antenna.^21–23^ Although this is an ongoing research area, at present only a few molecular motifs have been characterized with respect to their two-photon isomerization properties. The most frequent compound-class in this regard are azo molecules which undergo *E*/*Z* reactions after two-photon excitation.^19,20,24^ The typical photoconversion yields in these molecules are of the order of 30%, and their two-photon cross sections can reach, – in certain cases – a few hundred GM units.^19^ Other examples of two-photon reactive molecules include caged systems,^8,13^ Schiff bases, and azlactone compounds.^20^

As can be seen, in most of these systems the photochemical reaction is a simple conformational change around a double bond. From the point of view of the design of new photo-controllable systems with non-linear optical properties, it is desirable to explore other kinds of reactions where the molecular transformation is more pronounced than an *E*/*Z* isomerization.

**Scheme 1:**
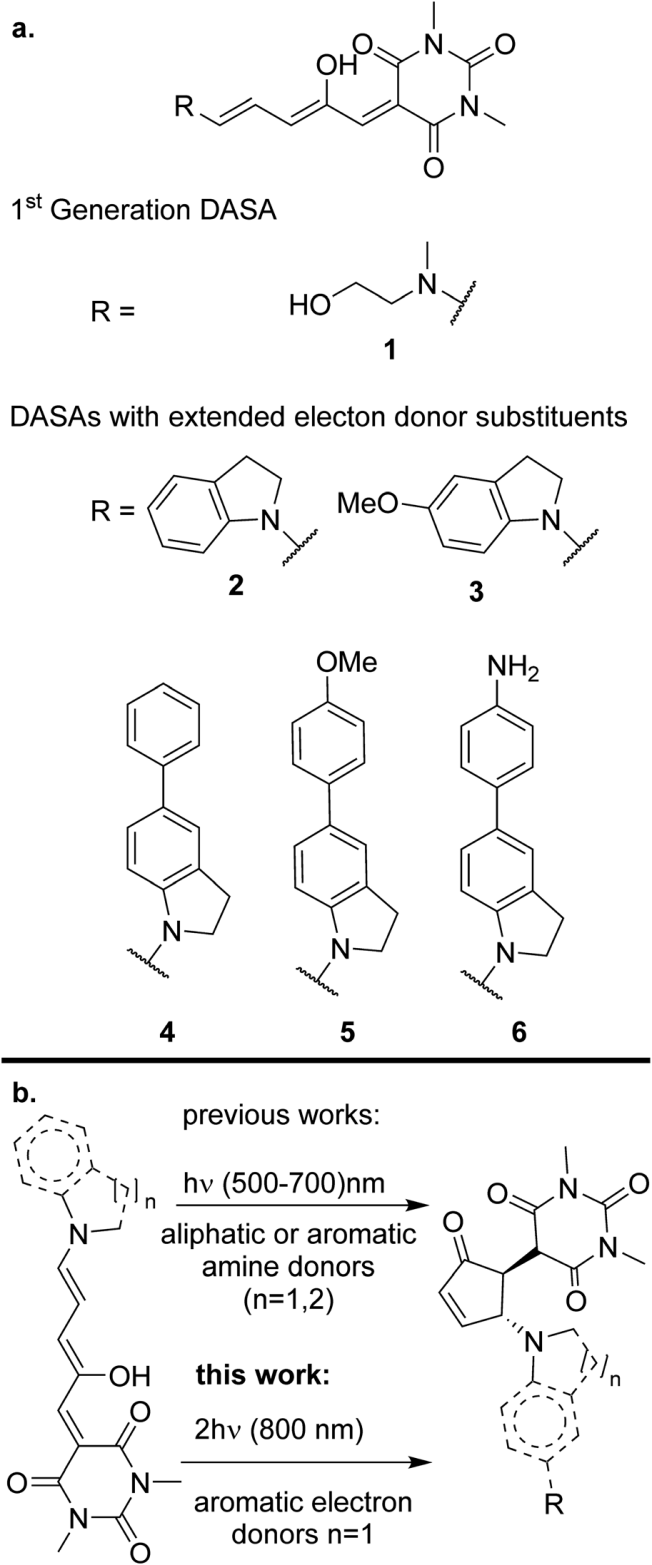
(a) Structures of DASAs studied in this paper and (b) photoisomerization of DASAs.

In this contribution we demonstrate for the first time that donor–acceptor Stenhouse adducts (DASAs) are promising options for isomerization processes induced by two-photon absorption. DASAs are a family of photochromic pigments formed by an amine electron-donating group bonded to a polymethinic chain. The polyconjugated chain is bonded to a carbon-based electron-accepting group frequently derived from a Meldrum or barbituric acid.^25,26^ The extended conjugation and donor–acceptor structure in these molecules grants them an intense absorption band in the visible region. The exact position of this band is highly sensitive to the substitution pattern at the end groups, so that it can be chemically tuned from 500 to 750 nm. Upon light absorption, DASAs undergo a rapid excited state *E*/*Z* transformation in the 3rd methinic group. This isomerization step is followed by further evolution through a ground-state thermal electrocyclic reaction to form a cyclic pentenone as shown in Scheme 1.^27^ The product of this light-initiated reaction lacks the extended conjugation of the precursor and only absorbs in the UV region.^28^ This structural change is large as the molecule evolves from a fully extended system to a much more compact structure. Such property has made DASAs excellent candidates to work as actuators to induce the release of the content of micelles and other microscopic drug delivery systems.^29,30^ This is one of the most promising applications of the non-linear photochemistry of the molecules of this contribution.

Despite the general interest in the photochemistry of DASAs, this is the first experimental study of their non-linear optical isomerization properties. For the present contribution we synthesized a set of DASAs with varying electron donating groups. The molecular structures are included in Scheme 1. On the donor side, we included indoline substituents with methoxy, phenyl, 4-methoxyphenyl and 4-aminophenyl groups. On the electron-accepting side we chose the barbituric acid group. Also, for comparison purposes we studied a first generation DASA formed from *N*-(2-hydroxyethyl)-*N*-methylamine with the same barbituric acid as acceptor.

We present a detailed study of the two-photon conversion properties of molecules 1–6 by following the photo-induced transformations which result from the irradiation with 800 nm femtosecond pulses. Significant differences in these molecules were observed from measurements of their biphotonic isomerization yields. Prior to the biphotonic studies, the linear spectra and photochemistry of these systems was characterized in some detail since knowledge of the absorption coefficients and photo-conversion yields was required to quantify the two-photon isomerization properties. Calculations at the TDDFT level together with the use of a quadratic response method were made to characterize the electronic transitions with regards to their two-photon activity, specifically to identify the specific higher lying singlet excited state responsible for the non-linear optical properties of these molecules.

## Materials and methods

**Scheme 2:**
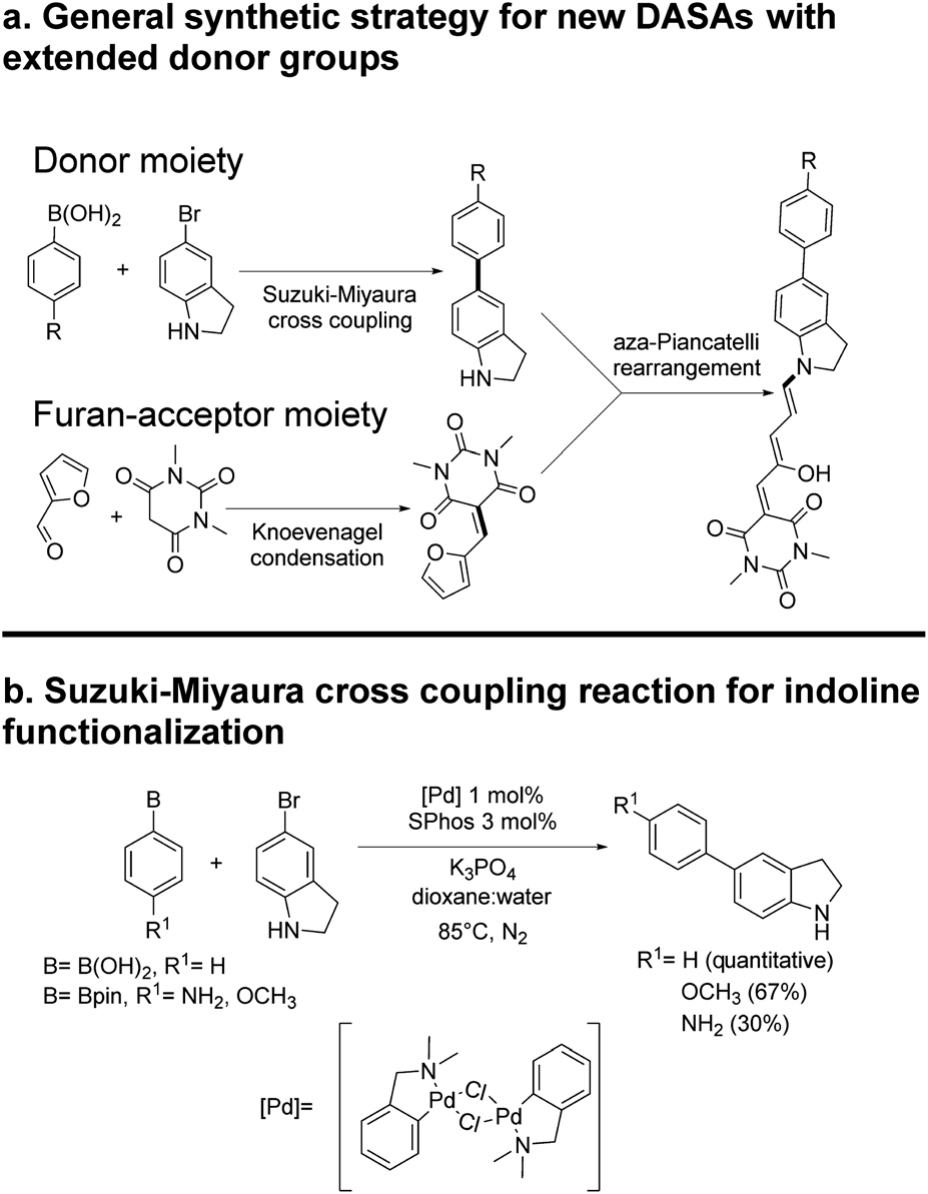
Synthesis of DASAs 2–6.

The general synthetic procedure for molecules 1–6 is shown in Scheme 2. Details are included in the ESI.† All materials were purchased from Sigma-Aldrich and used without further purification unless otherwise stated. NMR spectra were acquired in a 700, 500 or 400 MHz Bruker Avance spectrometer, or a 300 MHz Bruker Fourier equipment. The 700 MHz spectrometer is equipped with a 5 mm *z*-axis gradient TCI cryoprobe. All mass spectra were obtained at high-resolution with a JEOL JMSAX505 Spectrometer. Toluene, chloroform, methylene chloride and chlorobenzene solvents for the spectroscopic and photochemical studies were HPLC grade.

For the single photon conversion experiments, we used a 632.8 nm He–Ne laser, attenuated to 0.25 to 0.5 mW at the sample. The source for non-linear excitation was an 800 nm attenuated 1 kHz sub-picosecond pulse train from a Ti:sapphire laser (Legend Amplifier, tuned to 800 nm). To obtain easily measurable photoconversions from two-photon excitation, the beam was brought to a slow focus with a telescopic lens pair (100 and −120 mm focal lengths) giving a large Rayleigh range of 10 cm. This allowed for a near collimated beam at the sample which was placed in a 1 cm optical cell. The beam waist at the sample was 1.8 mm as measured by the knife-edge technique. The pulse duration of 265 fs with the same lens system in place was measured with a commercial autocorrelator from Coherent. The beam was sent directly to the sample inside a Cary-50 UV-Visible spectrometer so that the changes in the solutions could be made directly after different irradiation times or pulse-energies.

### Computational methods

The equilibrium open geometries were obtained in three steps. First, we carried out a conformational search for molecules 1–6 using the basin hopping method, employing the MMFF force field and the TINKER program.^31,32^ Subsequently, an additional gas-phase optimization was performed at the B3LYP/6-31G* level, using the GPU code TERACHEM.^33^ Finally, the geometries were re-optimized using the DFT method with the def2-SVP basis set, including dispersion corrections (D3BJ), and considering implicitly the effect of the solvent through the polarizable continuum model (PCM, toluene).^34–36^ The nature of the minima was verified by evaluation of the vibrational frequencies. In agreement with the experimental observations, only unfolded structures were retained in the analysis.

Regarding the choice of the DFT and TDDFT model, the optimally tuned range-separated functional OT-CAM-B3LYP (*α* = 0.0, *β* = 1.00) was chosen. This, since previous work has shown that the inclusion of exact exchange at large interelectronic distances is necessary for the description of charge transfer transitions.^37^ It is well known that the *μ* parameter involved in the partitioning of 1/*r*_12_ into short and long-range components, is dependent on the molecular system. Therefore, non-empirical tuning of *μ* to satisfy Janak's theorem (−*ε*_HOMO_^*N*^ = IP^*N*^) relating the ionization potential IP to the HOMO energy, leads to a systematic improvement in the performance of this type of functionals.^38–42^ For molecules 1–6, the parameter *μ* was determined by minimizing the following function:

1 *J*(*μ*) = [*ε*_HOMO_^*N*^(*μ*) + IP^*N*^(*μ*)]^2^

for which it was necessary to perform single-point calculations for the neutral system and the corresponding cation (IP^*N*^ = *E*^*N*−1^ − *E*^*N*^), with *μ* ranging from 0.040 to 0.085 using a step size of 0.001. Subsequently, the {*μ*, *J*} data were fitted by interpolation using cubic splines. The optimal values for *μ* are included in the ESI.†

For the evaluation of the two-photon properties of the excited states, the TDDFT method was also employed. The second transition moments, necessary for the determination of the two-photon absorption cross sections *σ*_2_ for each excited state were obtained from the residues of the quadratic response function. The electronic structure calculations were carried out with the Gaussian 16 program, and the Dalton 2020.1 program was used for the quadratic response calculations.^43,44^

## Results and discussion

### Synthesis

The general synthetic route is summarized in Scheme 2, where DASAs 2 and 3 have been reported previously.^22,25^ The overall yields for DASAs 1–6 were from 15 to 56%. Molecules 1–3 were synthesized according to procedures in the literature. Compounds 4–6 were obtained from carbon–carbon coupling reactions of 5-bromoindoline (Scheme 2), followed by the well-established reaction with the furan-acceptor moiety derived from the Knoevenagel reaction of furfural with barbituric acid.^26,28^ Detailed procedures are included in the ESI.†

To verify the structures and determine the relative populations of the two forms of these DASAs (polymethine *vs.* cyclopentenonic), we made an ^1^H-NMR analysis in deuterated DMSO (1–3) or methylene chloride (4–6). The spectra are included in the ESI.† Due to the presence of both forms in fresh solutions at room temperature, the relative populations were necessary to determine the linear absorption coefficients of the open forms, which in turn were required for the determination of the two-photon cross section (*σ*_2_) values. This is further discussed in the next section.

### Steady state spectroscopy

**Fig. 1:**
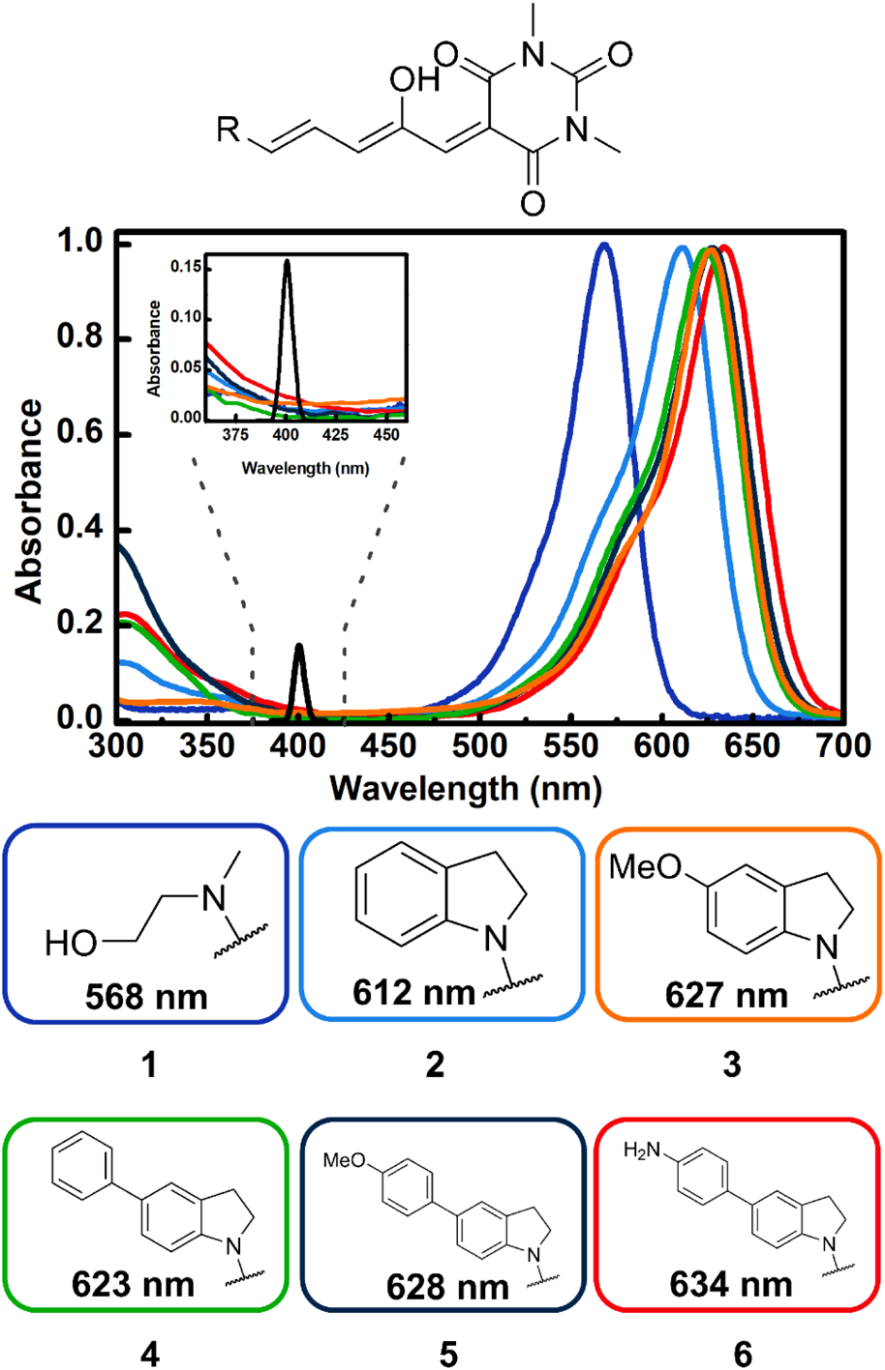
Absorption spectra of DASAs 1–6 highlighting the structural-dependence in toluene. The donor groups R are color coded with the respective spectrum. The inset shows the details of the region from 375 to 425 nm, together with the spectral intensity profile for the two-photon spectrum of the 800 nm excitation pulses (black line, two photon equivalent maximum wavelength: 400 nm).

As can be seen in Fig. 1, compounds 1–6 show a characteristic absorption band in the visible region due to the open form, and a higher energy band system with origins from 380 to 420 nm. As mentioned, the cyclopentenone species only has significant absorption below 350 nm.^28^

The relative populations of the two isomers in the fresh samples were determined through analysis of their ^1^H-NMR spectra. The peaks associated to each isomer were distinguished through their ^1^H-NMR signals before and after irradiation with visible light, assigning the signals that grow upon irradiation, as those from the closed forms. The integrals of the polymethine bridge proton signals at 6.20 and 6.80 ppm were used for the open form, while for the closed isomer, the methinic proton at the cyclopentenone ring and the indoline aromatic proton near 6.46 ppm were used. This is highlighted in Fig. S1–S6.† The open : closed relative populations were 69 : 31 for 4 and 5, and 32 : 68 for 6 in freshly prepared solutions (thermal equilibrium can take several days). This procedure was not necessary for 1 and 2 since they were not included in the determinations of the two-photon cross-sections (see below). For molecule 3 the absorption coefficient of the open form has been reported previously.^25^

To determine the absorption coefficients for 4–6, the solutions were fully converted to the cyclopentenone isomers through irradiation with visible light (given the lack of absorbance of the closed form in the visible region, the systems can easily be brought to full conversion to the pentenonic isomers). Using these spectra together with the open : closed mole fractions obtained from NMR in the initial solutions, the extinction coefficients of the open forms were calculated from the total absorbance of the samples according to:2
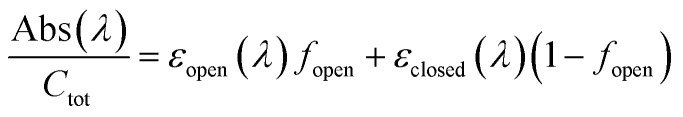
where Abs(*λ*) is the total absorbance of the sample, *C*_tot_ is the total molar concentration, *ε*_open_(*λ*) and *ε*_closed_(*λ*) are the molar absorption coefficients of the open and closed forms, and *f*_open_ is the mole-fraction of the open form. Detailed calculations are included in the ESI.† The UV-Vis spectra of the solutions fully converted to the closed forms are also included in ESI in Fig. S7–S9.† The molar coefficients of the open forms at the first band maxima are included in Table 1 and range from 124.2 × 10^3^ to 201.5 × 10^3^ M^−1^ cm^−1^.

**Table 1:** Photochemical measurements of DASAs 3–6

DASA	*ε* [M^−1^ cm^−1^]	Photoisomerization quantum yield	*σ*_2_ [GM]
3	100 × 10^3^^a^	0.30 ± 0.006	60 ± 1.7
4	201.5 × 10^3^^b^	0.15 ± 0.006	351 ± 15
5	188.5 × 10^3^^c^	0.17 ± 0.005	615 ± 13.5
6	124.2 × 10^3^^d^	0.25 ± 0.009	135 ± 5.8

a As reported by Helmmer *et al.*

b At 623 nm.

c At 628 nm.

d At 633 nm. The two-photon absorption cross sections were measured at 800 nm.

As can be seen in the spectra of Fig. 1 the position of the lowest energy open isomer band is highly sensitive to the electron donor group. The 1st generation DASA 1 has a maximum at 568 nm. This maximum shifts to 612 nm for the unsubstituted indoline 2, and upon addition of extra electron donating substituents at position 5 of the indoline moiety (*para* position with respect to the anilinic C–N bond), the spectrum red-shifts up to 634 nm for the 4-aminophenyl indoline molecule 6 in toluene. Important to the two-photon absorption properties of these molecules (see below), the onset of the high energy absorption bands also shifts to values up to 420 nm with the addition of electron donating substituents. These transitions correspond to higher energy singlets (S_*n*_, *n* > 1).

### Linear photochemistry

As a first photochemical characterization step before the non-linear optical studies, we measured the linear photoconversion quantum yields of 2–6 with 632.8 nm excitation. These yields were used in the calculations to determine the biphotonic absorption cross sections from the transformations to the closed photoproducts upon pulsed NIR excitation. The measurements of the yields were done by the method of Stranius *et al.*, using the absorbed photon count together with the bleaching of the first band (see ESI† for details).^45^ Linear excitation of 2–6, produces changes in the UV-Vis spectra consistent with a polyene (open) to cyclopentenone (closed) photoreaction with isosbestic points in the region around 325 nm for the new systems. The spectral evolutions are shown in Fig. S10–S17.†

The overall, open to closed quantum yields range from 15 to 52% and are included in Table 1. It should be emphasized that these yields account for several steps: a first *E*–*Z* photoisomerization stage, followed by the thermally activated formation of the cyclic forms.^29^

### Two-photon conversions

**Fig. 2:**
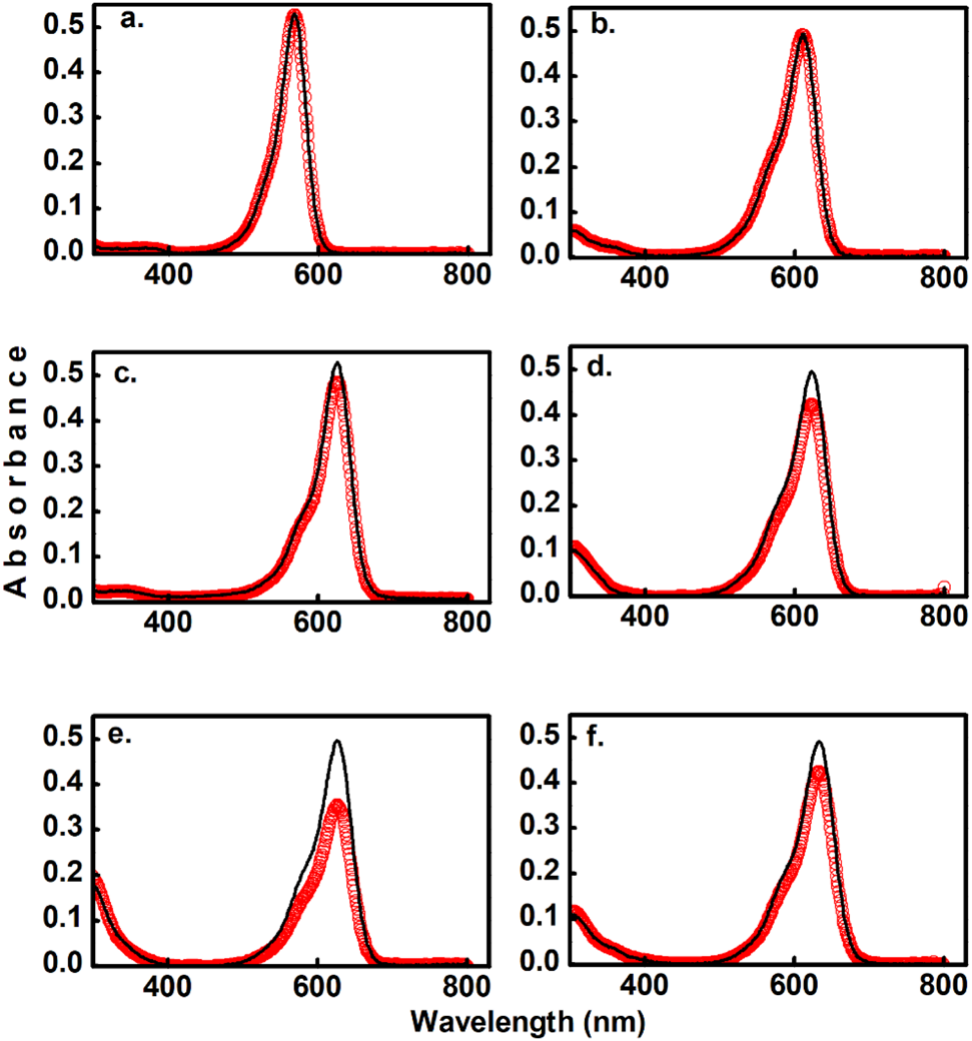
Comparison of two-photon induced spectral changes for DASAs 1 (a), 2 (b), 3 (c), 4 (d), 5 (e), and 6 (f) in toluene. The black lines indicate the absorbance before 800 nm irradiation, and the red symbols indicate the spectra after 5 min irradiation with a 1 kHz pulse train with pulsed intensity of 3.7 GW cm^−2^. The sample volume was 3 ml. These partial conversions were made to directly compare the two-photon reactivity of the different compounds.

As the main objective of this contribution, we have studied the photoconversion of 1–6 from non-linear excitation using a 1 kHz 800 nm sub-picosecond pulse train. First, the biphotonic isomerization in DASAs 1–6 was evaluated by irradiating the solutions (1–3 ml under constant stirring) for 5 min with pulsed intensities of 3.7 GW cm^−2^. In these conditions, only DASAs 3–6 underwent photo-switching as shown in Fig. 2. The spectra of these DASAs changed in an identical manner as in the linear photochemical studies showing the same isosbestic points (see ESI†). From this observation, it can be concluded that only open-to-closed photoreactions took place at this pulsed intensity. In fact, these molecules undergo a full return to the thermal distribution of the isomers as we show in a section below. For DASA 1, we did not observe any transformation for intensities up to 18 GW cm^−2^, while for 2 -with the unsubstituted indoline donor, only 1–2% spectral changes occurred in these conditions. From these results the biphotonic studies were continued only for 3–6.

**Fig. 3:**
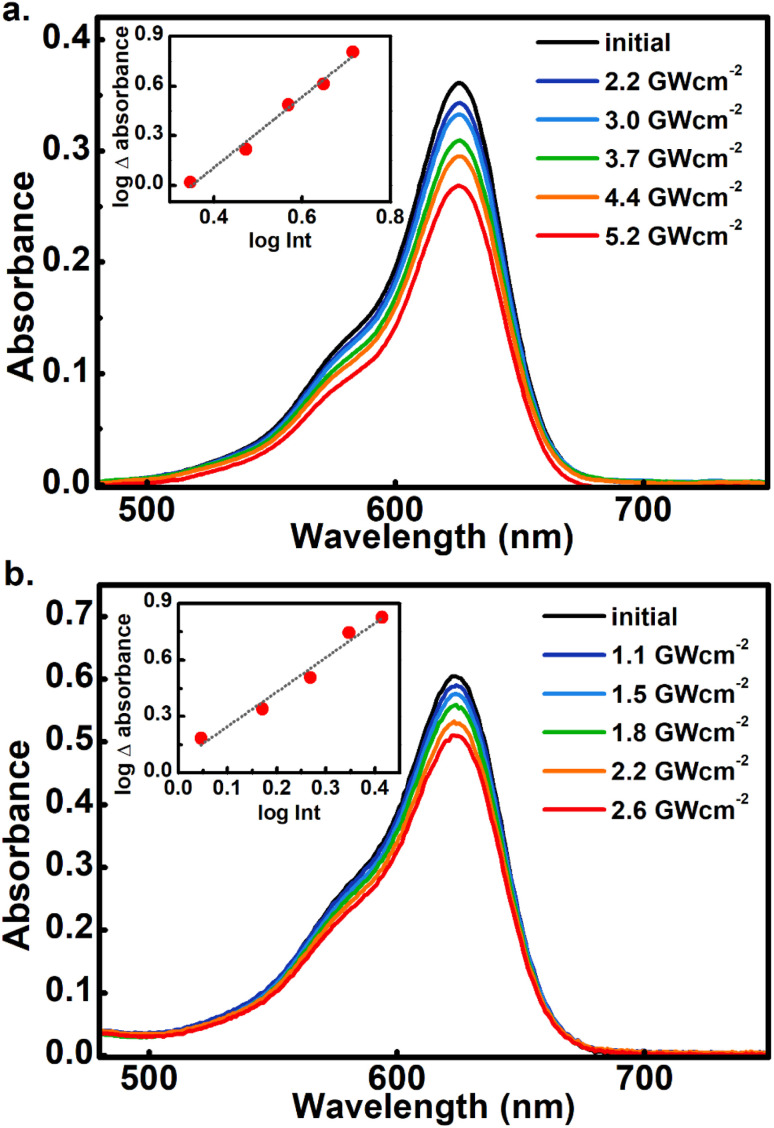
Absorbance changes for 3 (a) and 4 (b) in toluene upon 800 nm irradiation with different pulsed intensities. Inset: linear fits of the change in absorbance *vs.* intensity in log–log plots. The slope for 3 was: 2.1, *R*^2^:0.99. For 4 the log–log slope was 1.8, *R*^2^ = 0.97. For each intensity, a fresh solution sample was used.

The square dependence of the 800 nm photo-transformations as a function of intensity was verified for these molecules. The respective visible-band bleachings are shown in Fig. 3 for compounds 3 and 4, and in Fig. S18 and S19† for 5 and 6. The insets include the absorbance changes as a function of pulse intensity in log–log plots, showing the biphotonic behavior. It should be noted that for intensities above a certain value for each molecule, the behavior deviates from the two-photon square dependence, most likely due to saturation effects as observed for other systems at similar intensities.^11^

**Fig. 4:**
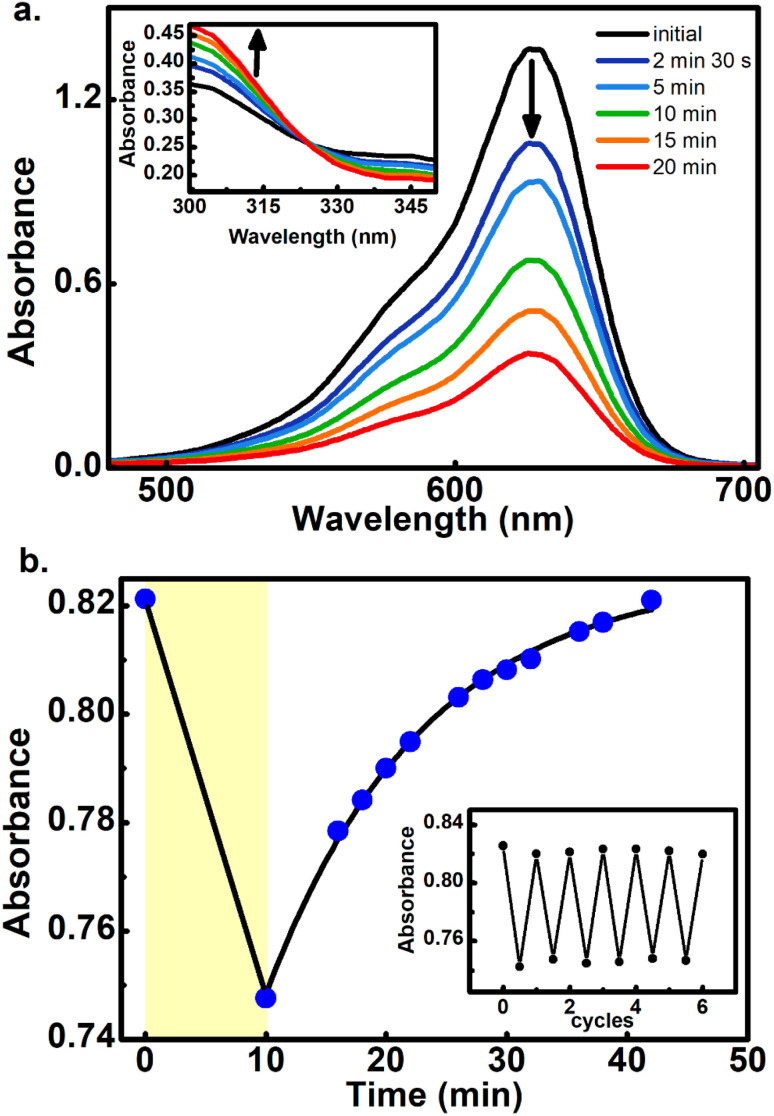
(a) Biphotonic 800 nm conversion of 5 at 3.70 GW cm^−2^ showing the sample absorbances at different pulsed irradiation times. Inset: zoom of the region around the isosbestic point (325 nm). (b) Biphotonic bleaching of 5 measured at 632 nm and the respective thermal return in chlorobenzene at 50 °C. The return to the equilibrium absorbance level was fitted to an exponential model giving an observed return rate constant of *k* = 0.076 min^−1^, (*R*^2^ = 0.99). The yellow zone corresponds to the irradiation period. The inset shows several cycles of photo-transformation and thermal returns.

To further demonstrate that the 800 nm biphotonic events give rise to DASAs open-close transformations, we also followed the evolution of the samples as a time-series by following their UV-Vis spectra during the 800 nm irradiations. As we show in Fig. 4 and S20–S22,† as the samples are exposed to the femtosecond pulse train, the spectra show the same behavior as in the linear excitation experiments, with a continuous bleaching of the visible bands, and increases in the UV region. Again, the same isosbestic points were observed as in the linear excitation cases.

Furthermore, the reversible nature of the non-linear photoisomerization reactions was verified for 4–6 from the thermal returns of the solutions to their pre-irradiation states. These thermal-return experiments (Fig. 4 for compound 5, and Fig. S23 and S24† for compounds 4 and 6) were performed after irradiation in chlorobenzene as solvent and at 50 °C since in these conditions the cyclopentenone to polyene thermal equilibration occurs on time scales of only several minutes.^28^ In these experiments, molecule 4 showed a small degree of photochemical fatigue after a few cycles, while 5 and 6 gave full returns to the original absorption values for at least several cycles. The degree of photoconversion for the two-photon experiments of 5 and 6 can actually be brought to 100%, which indicates that the two-photon absorption cross section of the closed form is much smaller than that of the extended polymethinic form. This is consistent with the large change in conjugation and push–pull character between the two isomers.

It should be mentioned that this is the first verification of multiple biphotonic reversible transformations of isomerizable molecules, where several cycles were performed without significant fatigue. Such behavior indicates that after appropriate optimization, DASAs can be interesting candidates for reversible absorbance schemes in the visible region, controlled through non-linear optical transformations. The present contribution then points towards clear non-linear photochemistry which does not appear to differ mechanistically from the single photon case.

### Two-photon cross section measurements

Foreseeing potential applications,^7,46^ knowledge of the two-photon absorption cross sections of these molecules is crucial. DASAs are minimally fluorescent, so attempts to detect their emissions upon two-photon excitation were unfruitful. The use of Z-scan type measurements suffers from appreciable changing molecular concentrations due to the rapid photoconversion of the solutions. However, the effective non-linear cross sections of these molecules can be determined from the quantification of their open-closed biphotonic conversion under well-characterized irradiation conditions and taking into consideration the photoreaction yields. Specifically, we measured the concentration changes of 3 ml solutions of 3–6 under timed exposure to the 800 nm pulse train. The visible band absorbance changes of the open form, together with the photochemical yields and extinction coefficients were then used to determine the respective two-photon absorption cross sections according to the procedure described below.^47^ First, in order to validate this method which uses a 1 kHz pulse train with a large 1.8 mm beam waist, we measured the two-photon absorption coefficient of rhodamine 6G/methanol through the reference biphotonic fluorescence procedure using a spectrograph and lens system at 90° in the same setup (we used rhodamine B/methanol as a standard, obtaining values within 10% of the reported value).^44^ It should be emphasized that the irradiance levels with this setup (of the order of 1 GW cm^−2^) are the same as those commonly used with nanojoule level pulses with focusing systems for spots sizes less than a micron wide.

The two-photon absorption cross section *σ*_2_ quantifies the changes in photon flux from losses due to molecule–light interactions through the third order susceptibilities at a given wavelength:^47,48^3
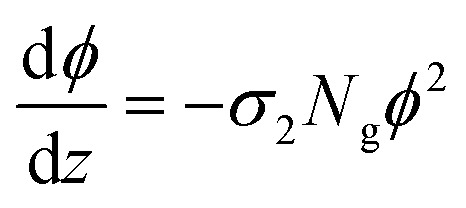
where *ϕ* is the photon flux, *N*_g_ is the ground state molecular concentration, and *z* defines the beam propagation direction. This equation calculates the number of photons absorbed per unit time and unit volume, therefore, the number of molecules excited per unit time and unit volume *n*^(2)^ is:^47^4
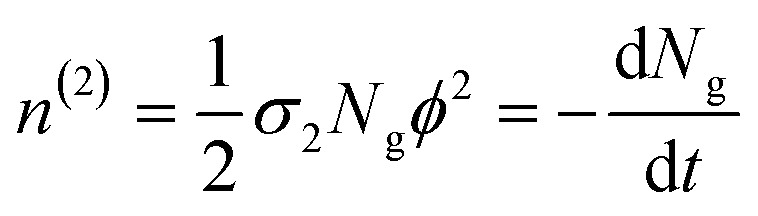


In eqn (4), the ½ factor is because two simultaneous photons produce a single molecular excitation. Eqn (4) also calculates the time derivative of the molecular ground state concentration change with respect to time: d*N*_g_/d*t*.^47^ Considering a pulse duration *τ*, and an initial ground state concentration *N*_o_, the number of excited molecules by a single pulse can be calculated from the integration of eqn (4) giving the following form for the number density of molecules left in the ground state after a pulse:5
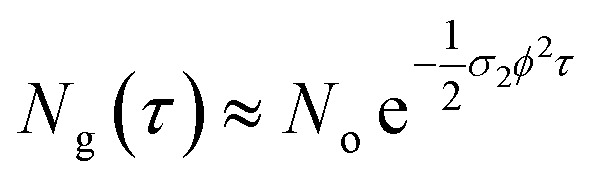


Our measurements of the two-photon absorption cross sections at 800 nm are based on the absorbance changes at the first band maxima after an irradiation time of 300 s with a 1 kHz repetition rate sub-picosecond pulse train in samples under constant stirring. This irradiation produces an absorbance bleach related directly to total number of photoconverted molecules per unit volume in the sample: *N*^total^_isom_. The number of molecules photoconverted per pulse *N*_isom_ was obtained through division by the total excitation pulses in the irradiation time. As mentioned previously, these measurements require knowledge of the open form absorption coefficient to determine the actual open form initial molecular concentration *N*_o_, and the concentration change from the two-photon reactions. The photon flux at the sample is known from the pulse energies (20 to 200 mJ), beam waist *w*_o_ (0.18 cm) and pulse duration *τ* under the approximation of a uniform irradiated area *a* = π*w*_o_^2^, considering a constant photon flux during the 265 fs full width at half maximum of the pulse intensity profile. From the measurements of isomerized molecules per pulse, the number of photoexcited molecules *N*_o_ − *N*_g_ per pulse was calculated by dividing *N*_isom_ by the open-closed reaction yield which was obtained from the previous monophotonic experiments.

These measurements were repeated for five different excitation pulse energies. This was done in order to verify that no self-focusing or saturation effects are influencing the experiments. In these five experiments, we obtained consistent two-photon cross sections as shown in Tables S3–S6.† The final *σ*_2_ values we report in Table 1 correspond to fits of the linear form of eqn (5) by varying *ϕ*^2^ as the independent variable:6
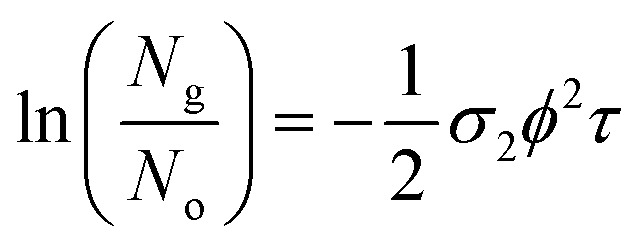


The results for the *σ*_2_ estimates are included in Table 1 and range from 60 to more than 600 GM. As can be seen, DASAs 3–6, have significant non-linear cross sections and are also the molecules where the first and second UV-Vis bands are most red shifted of the group. Specifically, these are the molecules where the onset of the second band systems lies at around 400 nm as highlighted in Fig. 1.

The two-photon energy of the 800 nm pulses with a spectral full width at half maximum of 15 nm, corresponds to 3.1 eV ± 0.03 eV, which corresponds to the energy zone near the onset of the UV absorbing bands of 3–6. Taking this into consideration, it can be suggested that the states involved in the transitions for non-linear excitation in these DASAs are higher-lying singlets with moderate to minimal linear absorbances (as is frequently encountered for two-photon active states, even in non-centrosymmetric molecules).^44^ These states should in any case be located above the S_1_ state, since the two-photon equivalent-wavelength (400 nm) is more than 200 nm from the first singlet bands (∼600 nm) as shown in Fig. 1. Considering typical rates for internal conversion dynamics from the higher singlets to the S_1_ level for molecules in solution (of the order of 10^14^ s^−1^), it is expected that if the systems are populated to higher electronic states, the photochemical step in these DASAs can occur from either the original higher states, or from the S_1_ state after internal conversion. This issue is further explored through computational approaches in the next section.

### Computational results

TDDFT studies were made on systems 1–6 to determine which are the excited states with significant non-linear absorbance properties. We employed the optimally tuned range-separated functional OT-CAM-B3LYP,^39–42^ the def2-SVP basis set,^34^ including dispersion corrections (D3BJ),^35^ and the PCM model for the solvent (see methods section).^36^ The usage of a tuned functional in molecules with significant charge-transfer characteristics is crucial for an accurate prediction of the transition energies and overall excited state properties.^37,39–42^ The values of the respective range separation parameters are included in Table S7.† Once the excited states were determined with excellent matches with the experimental spectra in solution (see below), we estimated the two-photon absorption properties of each state through the quadratic response method which calculates the two-photon transition moments from the residues of the respective response functions.^38,49–52^

**Fig. 5:**
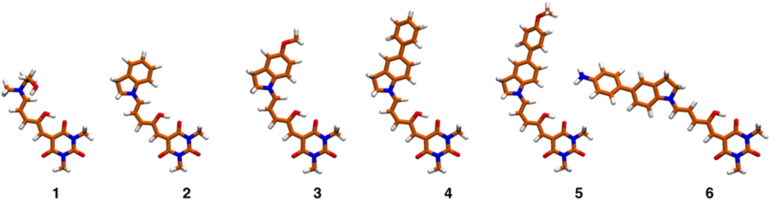
Equilibrium geometries corresponding to the global minima of molecular systems 1–6.

Fig. 5 shows the equilibrium geometries associated with the global minima of the Potential Energy Surface (PES) of molecules 1–6. Since the PESs exhibit several local minima with energies close to each other, there are several conformations that are thermally accessible at 298 K. These structures were determined as indicated in the methods section and are shown in Fig. S29 and S30.†Table 2 presents the excitation energies and oscillator strengths averaged over the thermally accessible geometries. The position and linear intensity of the electronic transitions change only slightly with the adopted geometries. For example, rotation around the first two bonds of the polymethylene bridge (from the indoline side) has a minimal effect on the ordering and position of the excited electronic states. Thanks to the use of a tuned functional and the averaging over the accessible geometries, the match with the experimental spectra is excellent as we show in Fig. S31.†

**Table 2:** Vertical transition energies (eV), oscillator strengths and two-photon absorption cross sections (GM) for the singlet excited states of 1–6, obtained from OT-CAM-B3LYP/def2-SVP calculations. The values are reported considering a Boltzmann averaging between the thermally accessible conformations (weights > 0.01) at 298 K

System		Transition energy [eV]		*f*	*σ*_2_^0*J*^ [GM]
		Calc.	Exp.		Calc.
1	1^1^ππ*	2.347	2.19	1.124	5.2
	1^1^nπ*	2.946		0.000	0.01
	2^1^ππ*	3.556		0.002	5.7
	2^1^nπ*	3.599		0.000	0.54
	3^1^ππ*	3.829		0.068	1098
2	1^1^ππ*	2.240	2.03	1.315	24.799
	1^1^nπ*	2.905		0.000	0.008
	2^1^ππ*	3.372		0.007	441.9
	3^1^ππ*	3.475		0.036	51.7
	4^1^ππ*	3.568		0.000	8.091
3	1^1^ππ*	2.10	1.98	1.40	55.5
	1^1^nπ*	2.84		0.00	0.02
	2^1^ππ*	3.01		0.03	1056
	3^1^ππ*	3.21		0.05	21.56
	4^1^ππ*	3.44		0.00	16.21
4	1^1^ππ*	2.055	2.03	1.627	24.42
	1^1^nπ*	2.773		0.000	0.01
	2^1^ππ*	2.975		0.011	3914
	3^1^ππ*	3.209		0.028	350
	4^1^ππ*	3.295		0.237	626.6
5	1^1^ππ*	2.135	1.98	1.483	133.3
	2^1^ππ*	2.852		0.070	3111
	1^1^nπ*	2.868		0.000	0.05
	3^1^ππ*	3.292		0.022	51.3
	2^1^nπ*	3.400		0.143	3.12
6	1^1^ππ*	1.943	1.96	1.633	623.4
	2^1^ππ*	2.442		0.159	4044
	1^1^nπ*	2.763		0.000	0.02
	3^1^ππ*	3.139		0.017	274.5
	4^1^ππ*	3.243		0.044	3387

**Fig. 6:**
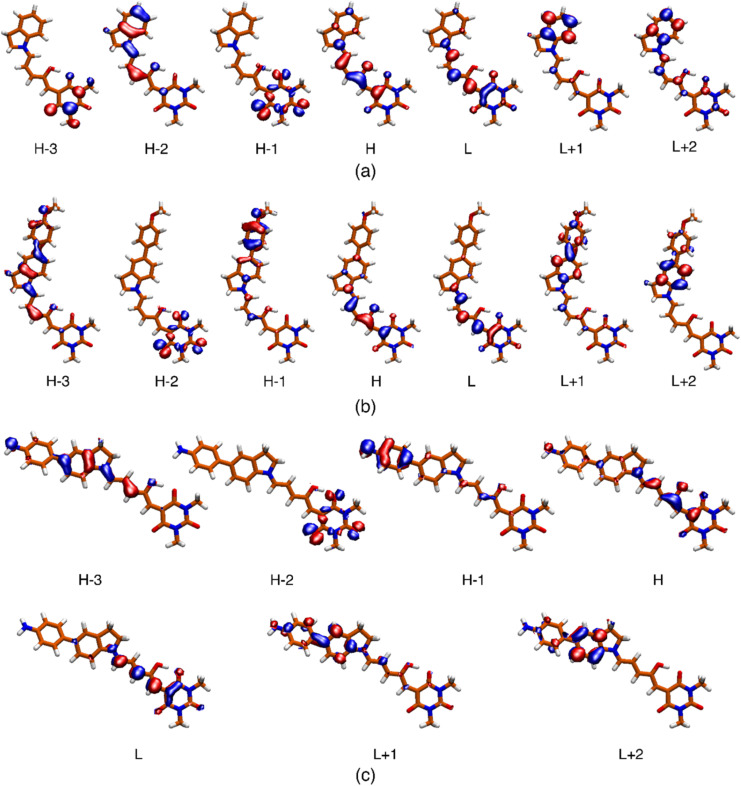
Isosurface (0.04) of the molecular orbitals involved in the description of the low-energy singlet electronic states for systems 2 (a), 5 (b) and 6 (c). The respective surfaces for molecules 1, 3 and 4 are included in the ESI.†

The most relevant Molecular Orbital (MO) isosurfaces of 1–6 are shown in Fig. 6 and S32.† It should be noted that for 1 (first generation DASA), the MOs are delocalized over the entire molecule. However, in the case of 2–6, there are MOs that have localized amplitudes either in the indoline or the methylurea moieties. The analysis of the TDDFT transitions reveals that the S_0_ → S_1_ excitation is well described by the HOMO–LUMO configurational change, giving rise to the first ^1^ππ* and most intense band in the visible region. The more localized MOs in systems 2–6 leads to the presence of intramolecular charge transfer states (^1^ππ*-ICT) above or near the energy of at least one ^1^nπ* state. These 2^1^ππ* charge transfer states (S_3_ or S_2_ depending on the system) have very small linear oscillator strengths and can be part of the red tail of the UV band systems of these DASAs. The onset of these features for molecules 3–6 is around 400 nm, and at higher energies for molecules 1 and 2 (see inset of Fig. 1). For a full description of these systems, Table S8† includes the transition energies for each of the conformers predicted to be populated at room temperature.

The macroscopic two-photon absorption cross sections from the ground state to state *J*: *σ*_2_^0*J*^ can be obtained from the rotationally averaged TPA strengths 〈*δ*^TPA^_0*J*_〉 as follows.^38,49–52^7
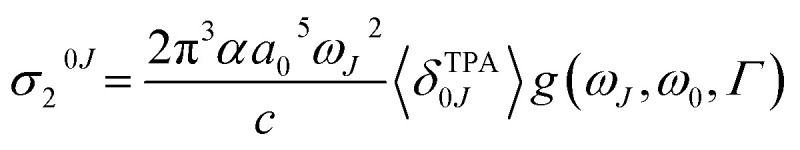
where *α* is the fine structure constant, *a*_0_ is the Bohr radius, *c* is the speed of light and *ω*_*J*_ = *E*_*J*_ − *E*_0_ is the energy difference between the |0〉 and |*J*〉 electronic states. For linearly polarized light with parallel orientation and two photons of the same energy, 〈*δ*^TPA^_0*J*_〉 can be obtained from the two photon absorption transition moments *S*_*ij*_:8
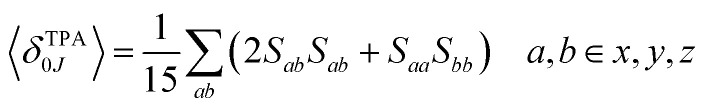


Assuming a Lorentzian function for the line shape function *g*(*ω*_*J*_, *ω*_0_, *Γ*), described by the parameter *Γ* (0.1 eV), the prediction at the maximum of each band corresponds to9
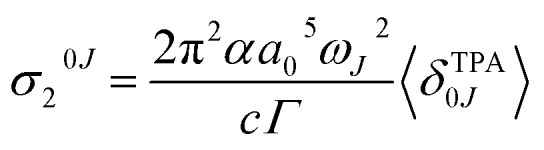


Table 2 includes the *σ*_2_^0*J*^ values evaluated from 〈*δ*^TPA^_0*J*_〉 according to the quadratic response method included in the Dalton program.^44^ Although this method is known to overestimate the values of the cross sections, which are also highly sensitive to the chosen *Γ* parameter, this method has been shown to be correct at discerning the two-photon activity of the different excited states in a molecule.^38,51,52^ The specific *σ*_2_^0*J*^ values for each relevant conformations of systems 1–6 are included in Table S9.†

For the case of the S_0_ → S_1_ transitions, the cross sections range from 24.7 GM for 2 to 654 GM for 6 (see Table S9†). Importantly, in contrast to 1, molecules 2–6 exhibit large cross sections for the transition to the second ^1^ππ* electronic states. For example, in the case of 4, the predicted *σ*_2_^0*J*^ value for the S_0_ → S_3_ transition is two orders of magnitude larger than for the S_0_ → S_1_ transition. As discussed previously, the conformational distribution does not significantly change the position or the nature of the involved states in the electronic transitions. However, the *σ*_2_^0*J*^ values are more sensitive to the adopted conformation. As a particular case, the rotation around the indoline to polymethine chain N–C bond in molecule 6 causes a decrease of the cross-section value from 4750 to 2790 GM.

The most relevant result from the computational study is that, as opposed to 1, molecules 2–6 show high values of *σ*_2_^0*J*^ for the states in the energy region around 2.5 to 3.5 eV. Therefore, the overall view that can be attained from the TDDFT calculations is that the inclusion of the substituted indolines as electron donors, significantly enhances the two-photon activity of these molecules. In fact, the predicted cross sections increase for DASAS with the extended donor sections, and furthermore with the electron donating groups. The predicted states responsible for biphotonic processes correspond to higher ^1^ππ* states with a significant charge-transfer character, where the dominant contribution is from HOMO−1 to LUMO+1 transitions. These results are consistent with the experimental observations where, as mentioned, the *E*–*Z* photoconversion of the open form of these DASAs can take place directly from the higher states, or after rapid internal conversion to the S_1_ state. It should be stressed that the single wavelength measurements at 800 nm of Table 1 most likely do not correspond to the maximum of a two-photon absorption band.

It should also be emphasized that although the calculated cross sections are most likely overestimated, the quadratic response method correctly predicts the induction of biphotonic activity from the electron donor groups and are even consistent with an increased two-photon absorbance for specific substitutions at position 5 of the indoline donor.

## Conclusions

We have characterized for the first time the two-photon isomerization properties of a series of Stenhouse adducts. Several of the compounds show robust isomerization reactions from the open form to the cyclopentenonic form upon irradiation with sub-picosecond pulses centered at 800 nm. For these molecules, the quadratic dependence of the isomer conversion as a function of pulse intensity was verified.

The study of the biphotonic conversion properties was based on a setup where the intensities were carefully adjusted with beam sizes of the order of a millimeter. This allowed for two-photon conversion in solutions where the isomer population changes could be measured directly. Importantly, the same intensity levels were kept as in setups which use more focused beam spots (∼1 GW cm^−2^), and the experimental setup was validated through comparisons with a fluorescent two-photon absorbing standard. The non-linear absorption cross sections were determined taking into consideration observed open-closed conversions. These measurements required precise knowledge of the isomerization quantum yields and the extinction coefficients of the open isomers from solutions where both isomers were present. The biphotonic cross sections range from 60 GM to more than 600 GM, which makes them excellent candidates for direct applications. Considering the 800 nm two-photon energy and the overall biphotonic activities, it is likely that the transitions involved in these processes correspond to higher-lying ^1^ππ* states. This correlates appropriately with the onset of the higher-energy absorbance features of the molecules near 400 nm.

The two-photon properties for each of the excited singlet states of these molecules were estimated through a quadratic response method. These calculations clearly ascribe important two-photon transition probabilities to the second excited singlet states with a ^1^ππ* character. In fact, in agreement with the experimental trend the transition energies for these states are predicted to be red shifted as a function of the electron donating capacity of the indoline substituents.

The biphotonic isomerization of these DASAs point towards their applicability in different schemes where photochemical reactions need to occur at highly localized volumes or at a variable depth. For example, the isomerization of DASAs has been proven to induce the release of active compounds from different kinds of nanoparticle carriers.^26,27^ The present results point towards applications of this kind through the use of NIR light in more focused volumes and at variable depths. Current efforts by our group include improving the water solubility properties of these systems and also including substituents that increase their inclusion into lipid membranes allowing applications in the release of the contents of liposomes and micelles.^53^

## Data availability

All data supporting the results discussed in this work is available within the paper and its ESI.†

## Author contributions

Francisco A. Reza-González: methodology, synthesis, investigation, validation. Emmanuel Villatoro: conceptualization, synthesis, methodology. Mariana M. Reza: methodology, investigation, validation, formal analysis. Jesús Jara-Cortés: computational studies. Héctor García-Ortega: synthetic methodology. Edgard F. Blanco-Acuña: synthesis and purification. José G. López-Cortés: synthetic methodology. Nuria Esturau-Escofet: NMR experiments. Alan Aguirre-Soto, methodology two-photon experiments, Jorge Peon: conceptualization, funding, supervision, writing, review and editing.

## Conflicts of interest

There are no conflicts to declare.

## Supplementary Material

SC-014-D3SC01223A-s001
