# Supplementary material for: Two-photon isomerization properties of donor–acceptor Stenhouse adducts
Source: Chem Sci. 2023 Apr 24;14(21):5783–94. doi: 10.1039/d3sc01223a (PMC10231324; doi:10.1039/d3sc01223a)
Supplement: SC-014-D3SC01223A-s001 [file SC-014-D3SC01223A-s001.pdf]

Electronic Supplementary Material (ESI) for Chemical Science.
This journal is © The Royal Society of Chemistry 2023

SUPPORTING INFORMATION

Two-Photon Isomerization Properties of Donor-Acceptor Stenhouse Adducts

Francisco A. Reza-González[†], Emmanuel Villatoro[†,£], Mariana M. Reza[†], Jesús Jara-Cortés[‡], Héctor García-Ortega[§], Edgard F. Blanco-Acuña[§], José G. López-Cortés[†], Nuria Esturau-Escofet[†], Alan Aguirre-Soto[φ] and Jorge Peon[†,*]

[†]*Instituto de Química, Universidad Nacional Autónoma de México, Ciudad de México, México*

[‡]*Unidad Académica de Ciencias Básicas e Ingenierías, Universidad Autónoma de Nayarit, Tepic 63155, México*

[§]*Facultad de Química, Universidad Nacional Autónoma de México, Ciudad de México, México*

[φ]*School of Engineering and Sciences, Tecnologico de Monterrey, Monterey, Nuevo Leon, Mexico*

[£]*Present Address: Department of Chemistry, Northwestern University, Evanston, Illinois 60208, United States*

* *Corresponding author*

Content

Content **2**

Synthesis and characterization **3**

Synthetic methods 3

Determination of the open-closed isomer proportions **9**

Steady-state spectroscopy **15**

Molar absorptivity coefficients ε(λ) 15

Linear photochemistry **18**

Photoisomerization quantum yields 18

Two-photon chemistry **23**

Intensity dependence of the photoisomerizations 23

Spectral evolutions during 800 nm pulsed irradiations 25

Two-photon absorption cross section measurements **29**

Computational results **33**

NMR Characterization of all the compounds **40**

Reference **79**

Synthesis and characterization

Synthetic methods

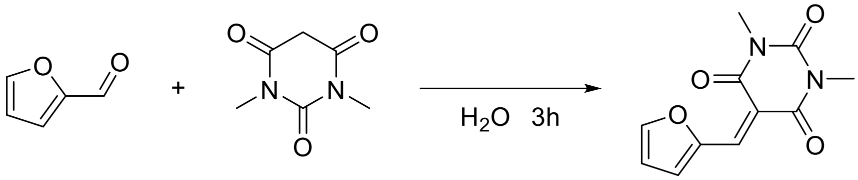

5-(furan-2-ylmethylene)-1,3-dimethylpyrimidine-2,4,6(1*H*,3*H*,5*H*)-trione: Freshly distilled 0.83 ml (10 mmol) of furfural was added to a solution of 1.56 g of barbituric acid in 40 ml of water. Color change to green was observed. The reaction was stirred for 3h at room temperature, time at which a thick slurry was formed. The reaction mixture was filtered. The filtered cake was redissolved in 100 ml CH_2Cl_2 and washed with 30 ml saturated $NaHSO_4$, 30 ml saturated $NaHCO_3$ and 30 ml brine. The organic layer was dried and the solvent was evaporated to yield 1.95 g of pure activated furane (83%). **^{1}H NMR** (400 MHz, $CDCl_3$, 298 K) δ 3.40 (s, 3H), 3.41 (s, 3H), 6.74 (ddd, *J* = 3.8, 1.6, 0.8 Hz, 1H), 7.85 (dd, *J* = 1.6, 0.7 Hz, 1H), 8.44 (t, *J* = 0.7 Hz, 1H), 8.64 (dt, *J* = 3.8, 0.7 Hz, 1H). **^{13}C NMR** (101 MHz, $CDCl_3$, 298 K) δ 28.37, 29.13, 111.59, 115.29, 128.18, 141.18, 150.53, 151.32, 151.54, 161.01, 162.62. **HRMS** (DART) *m/z* 235.07196, calc. 235.07188 for [M+1].

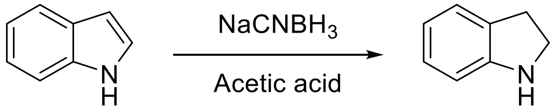

Indoline: 117.1 mg (1 mmol) of indole was dissolved with 3 ml of acetic acid in a 10 ml dry round-bottom flask suited with a water trap. Then 188 mg (3 mmol) of sodium cyanoborohydride were added. The solution was diluted with 15 ml of water two hours after stirring at room temperature and sodium hydroxide was added until pH=14. A white precipitate was formed. The aqueous solution was extracted with 3x35 ml CH_2Cl_2. The organic layer was washed with 2x50 ml water and 1x 50 ml brine, and dried with Na_2SO_4. The solvent was evaporated to yield 109 mg of pure indoline as a transparent oil. **^{1}H NMR** (301 MHz, $CDCl_3$, 298 K) δ 3.04 (t, *J* = 8.3 Hz, 2H), 3.56 (t, *J* = 8.3 Hz, 2H), 6.66 (dq, *J* = 7.7, 1.2, 0.6 Hz, 1H), 6.71 (td, *J* = 7.4, 1.1 Hz, 1H), 7.03 (tt, *J* = 7.7, 1.2 Hz, 1H), 7.13 (dq, *J* = 7.4, 1.2, 0.6 Hz, 1H). **^{13}C NMR** (76 MHz, $CDCl_3$, 298 K) 29.97, 47.45, 109.56, 118.78, 124.76, 127.34, 129.44, 151.73.

General procedure for Suzuki-Miyaura cross-coupling reactions: A clean Schlenk flask was charged with 5-bromoindoline (1 mmol, 1 eq.), boronic derivative (1.2 eq.), [Pd] (1 mol%), SPhos (4 mol%) and K_3PO_4 (1.2 eq. for boronic acid and 2.4 eq. for pinacol ester derivative). The reaction vessel was evacuated and backfilled with nitrogen gas 3 times. Then, previously distilled dioxane from sodium (4 ml) and water (0.8 ml) were added by syringe. The flask was placed in a preheated oil bath at 85 °C and stirred 24 h. The reaction mixture was cooled to room temperature, filtered through a short pad of celite, filter cake was then washed with 15 ml ethyl acetate. Then, 50 ml of water were added, and the aqueous layer were extracted with 3x20 ml ethyl acetate. The organic layer was washed with 2x30 ml water and 1x30 ml brine, dried with Na_2SO_4 and solvent evaporated. The pure product was obtained by silica column chromatography.

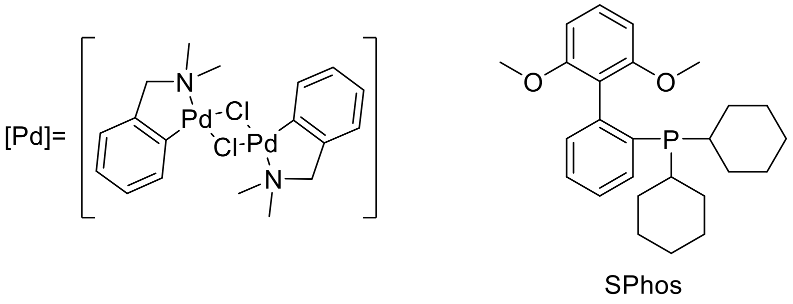

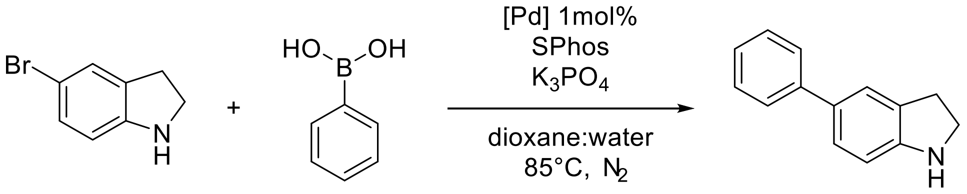

5-phenylindoline: The reaction was carried out according to the general procedure for Suzuki-Miyaura cross-coupling reactions. The crude was purified by flash chromatography silica column (80:20 hexane/EtOAc) to yield 390 mg (quantitative) of a yellow oil. **^{1}H NMR** (400 MHz, $CDCl_3$, 298 K) δ 3.10 (t, *J* = 8.4 Hz, 2H), 3.60 (t, *J* = 8.4 Hz, 2H), 6.72 (d, *J* = 8.1 Hz, 1H), 7.27 (m, 2H), 7.38 (t, *J* = 1.2 Hz, 1H), 7.39 (tt, *J* = 8.2, 1.7 Hz, 2H), 7.54 (dq, *J* = 8.2, 1.7, 1.5 Hz, 2H). **^{13}C NMR** (101 MHz, $CDCl_3$, 298 K) δ 29.93, 47.69, 109.65, 123.68, 126.22, 126.50, 126.69, 128.75, 130.21, 132.30, 141.88, 151.13. **HRMS** (DART) *m/z* 196.11283, calc. 196.11262 for [M+1].

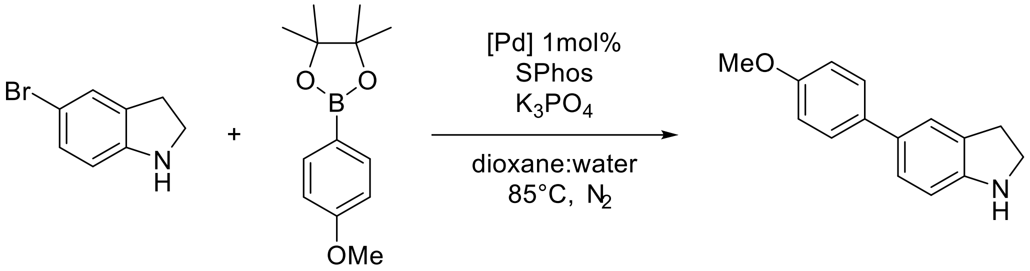

5-(4-methoxyphenyl)indoline: The reaction was carried out according to general procedure for Suzuki-Miyaura cross-coupling reaction. The crude was purified by flash chromatography silica column (80:20 → 75:25) to yield 149 mg of a white solid (67%). **^{1}H NMR** (400 MHz, $CDCl_3$, 298 K) δ 3.08 (t, *J* = 8.4 Hz, 2H), 3.60 (t, *J* = 8.4 Hz, 2H), 3.83 (s, 3H), 6.69 (d, *J* = 8.1 Hz, 1H), 6.93 (dt, *J* = 8.8, 2.1 Hz, 2H), 7.21 (dd, *J* = 8.1, 1.4 Hz, 1H), 7.32 (q, *J* = 1.4, 1.3 Hz, 1H), 7.45 (dt, *J* = 8.8, 2.1 Hz, 2H). **^{13}C NMR** (101 MHz, $CDCl_3$, 298 K) δ 29.81, 47.46, 55.32, 110.02, 114.07, 123.24, 125.95, 127.56, 130.29, 132.44, 134.39, 149.77, 158.35. **HRMS** (DART) *m/z* 226 for [M+1].

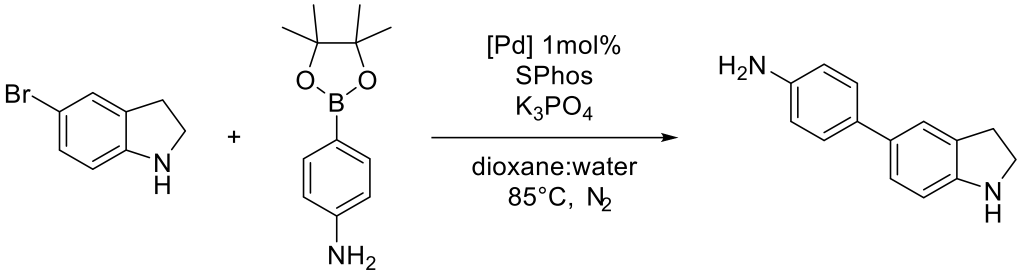

4-(indolin-5-yl)aniline: The reaction was carried out according to general procedure for Suzuki-Miyaura cross-coupling reaction. The crude was purified by flash chromatography silica column (5:5 hexane/EtOAc) to yield 62 mg of grey solid (30%). **^{1}H NMR** (300 MHz, $CDCl_3$, 295.5 K) δ 3.07 (t, *J* = 8.3 Hz, 2H), 3.59 (t, *J* = 8.3 Hz, 2H), 6.68 (dt, *J* = 8.0, 0.5 Hz, 1H), 6.72 (dt, *J* = 8.4, 2.0 Hz, 2H), 7.20 (ddt, *J* = 8.0, 1.9, 0.7 Hz, 1H), 7.31 (dt, *J* = 1.9, 0.5 Hz, 1H), 7.34 (dt, *J* = 8.4, 2.0 Hz, 2H).**^{13}C NMR** (75 MHz, $CDCl_3$, 295.5 K) δ 29.88, 47.56, 109.54, 115.45, 122.87, 123.00, 125.55, 127.47, 130.00, 132.46, 144.78, 150.29. **HRMS** (DART) *m/z* 211.12309, calc. 211.12352 for [M+1].

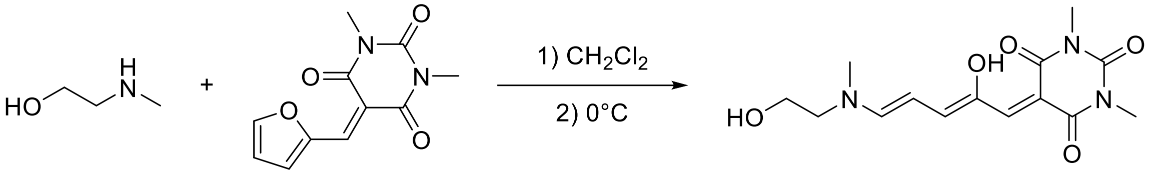

5-((2*Z*,4*E*)-2-hydroxy-5-((2-hydroxyethyl)(methyl)amino)penta-2,4-dien-1-ylidene)-1,3-dimethylpyrimidine-2,4,6(1*H*,3*H*,5*H*)-trione (1): To a solution of 192 mg activated furan dissolved in 10 ml CH_2Cl_2 1.1 eq. of 2-(methylamino)ethan-1-ol was added. Reaction was stirred 10 min at room temperature and cooled to 0 ºC and stirred 20 min more. Reaction was filtered and cake washed with cold Et_2O to yield 184 mg of pure **1** as a dark red solid. **^{1}H NMR** (500 MHz, DMSO-d_6, 300 K) δ 3.15 (s, 1H), 3.24 (s, 3H), 3.65 (ddt, *J* = 20.4, 8.7, 5.2, 3.2 Hz, 4H), 6.11 (dd, *J* = 13.1, 11.6 Hz, 1H), 6.69 (s, 1H), 7.19 (dd, *J* = 13.1, 1.6 Hz, 1H), 7.98 (d, *J* = 11.6 Hz, 1H), 12.52 (s, 1H). **HRMS** (DART) *m/z* 310.13948, calc. 310.14030 for [M+1].

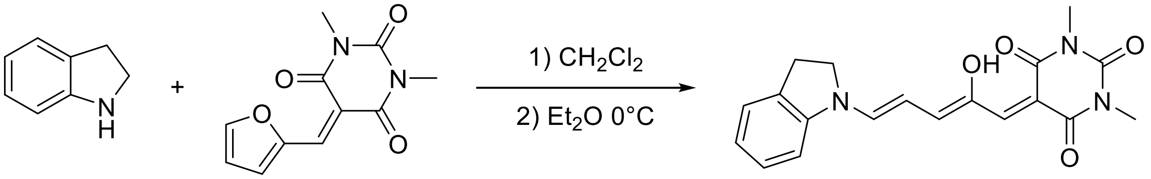

5-((2*Z*,4*E*)-2-hydroxy-5-(indolin-1-yl)penta-2,4-dien-1-ylidene)-1,3-dimethylpyrimidine-2,4,6(1*H*,3*H*,5*H*)-trione (2): 192 mg (1 mmol) of activated furan and 1.1 eq. of indoline were dissolved in 5 ml CH_2Cl_2. The reaction was stirred in darkness during 24 h, then solvent was evaporated. Residue is redissolved in 2 ml of CH_2Cl_2, cooled to 0 ºC and charged with 15 ml Et_2O. Then suspension is filtered, and cake was washed with cold methanol 3x10 ml to yield green to blue crystals of pure **2**. **^{1}H NMR** (500 MHz, DMSO-d_6, 300 K) δ 3.17 (s, 3H), 3.20 (s, 3H), 3.28 (t, *J* = 8.0 Hz, 2H), 4.22 (t, *J* = 8.0 Hz, 2H), 6.15 (t, *J* = 12.3 Hz, 1H), 7.04 (s, 1H), 7.17 (d, *J* = 12.3, t, *J* = 7.9, Hz, 2H), 7.34 (t, *J* = 7.8 Hz, 1H), 7.38 (d, *J* = 7.8 Hz, 1H), 7.48 (d, *J* = 7.9 Hz, 1H), 8.55 (d, *J* = 12.3 Hz, 1H), 12.46 (s, 1H). **HRMS** (DART) *m/z* 354.14434, calc. 354.14538 for [M+1]. This compound has been synthesized and characterized previously.[1]

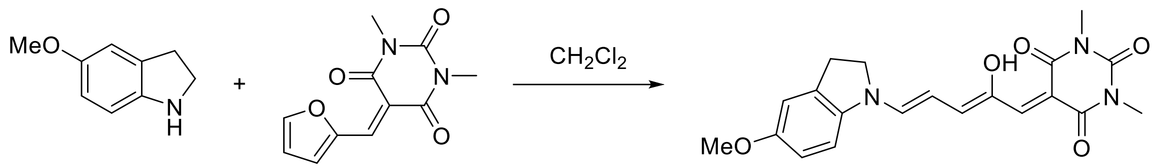

5-((2*Z*,4*E*)-2-hydroxy-5-(5-methoxyindolin-1-yl)penta-2,4-dien-1-ylidene)-1,3-dimethylpyrimidine-2,4,6(1*H*,3*H*,5*H*)-trione (3) ^{1}**H NMR** (500 MHz, DMSO-d$_6$, 300 K) δ 3.16 (s, 3H), 3.19 (s, 3H), 3.78 (s, 3H), 4.28 (t, J = 8.3 Hz, 2H), 6.17 (t, J = 12.3 Hz, 1H), 6.88 (s, 1H), 6.96 (dd, J = 9.2, 1.0 Hz, 1H), 7.01 (s, 1H), 7.14 (d, J = 12.3 Hz, 1H), 7.49 (d, J = 9.2 Hz, 1H), 8.56 (d, J = 12.3 Hz, 1H), 12.53 (s, 1H). **HRMS** (DART) *m/z* 384.15754, calc. 384.15595 for [M+1]. This compound has been synthesized and characterized previously, identical procedures were followed[1]

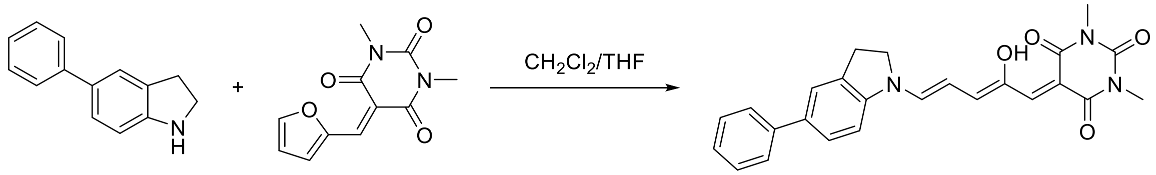

5-((2*Z*,4*E*)-2-hydroxy-5-(5-phenylindolin-1-yl)penta-2,4-dien-1-ylidene)-1,3-dimethylpyrimidine-2,4,6(1*H*,3*H*,5*H*)-trione (4): 132 mg of 5-phenylindoline were dissolved in 5 ml THF. To this solution, 132 mg of activated furan dissolved in 5ml CH_2Cl_2 were added. Reaction flask was placed in water bath at room temperature and stirred in darkness for 24h. After this time, a green slurry was observed. The reaction crude was filtered, and the cake was washed with cold methanol to yield 88 mg (37%) of pure blue crystals **(4).** Open form ^{1}**H NMR** (700 MHz, CD_2Cl_2, 300 K) δ 3.30 (s, 3H), 3.33 (s, 3H), 3.37 (t, *J* = 8.1 Hz, 2H), 4.17 (t, *J* = 8.1 Hz, 2H), 6.21 (t, *J* = 10.8 Hz, 1H), 6.79 (d, *J* = 10.7 Hz, 1H), 7.17 (d, *J* = 8.9 Hz, 1H), 7.35 (t, *J* = 7.4 Hz, 1H), 7.36 (d, *J* = 1.9 Hz, 1H), 7.44 (dd, *J* = 7.7 Hz, 3H), 7.53 (m, 1H), 7.58 (dd, *J* = 8.3, 1.2 Hz, 2H), 7.74 (d, *J* = 10.7 Hz, 1H), 12.41 (s, 1H). ^{13}**C NMR** (176 MHz, CD_2Cl_2, 300 K) δ 27.91, 28.56, 28.70, 49.87, 105.06, 110.29, 124.87, 127.10, 127.56, 127.79, 129.31, 131.50, 133.83, 138.53, 140.57, 143.57, 145.72, 148.57, 151.89, 157.84, 163.09, 165.73.

Closed form: ^{1}**H NMR** (700 MHz, CD_2Cl_2, 300 K) δ 3.06 (s, 3H), 3.10 (q, *J* = 8.5 Hz, 2H), 3.33 (s, 3H), 3.38 (q, *J* = 8.5 Hz, 1H, 15'), 3.53 (q, *J* = 8.5 Hz, 1H), 3.75 (dd, *J* = 4.3, 1.9 Hz, 1H), 4.09 (d, *J* = 2.0 Hz, 1H), 5.31 (dd, *J* = 4.3, 2.0 Hz, 1H), 6.42 (dd, *J* = 6.0, 2.1 Hz, 1H), 6.46 (d, *J* = 8.1 Hz, 1H), 7.23 (dd, *J* = 8.1, 2.0 Hz, 1H), 7.25 (t, *J* = 7.5 Hz, 1H), 7.36 (s, 1H), 7.38 (t, *J* = 7.5 Hz, 1H), 7.51 (dd, *J* = 7.5, 1.3 Hz, 2H), 7.71 (dd, *J* = 6.0, 2.1 Hz, 1H), ^{13}**C NMR** (176 MHz, CD_2Cl_2, 300 K) δ 28.56, 28.63, 28.87, 29.11, 47.55, 47.70, 47.98, 60.88, 106.72, 124.27, 126.40, 126.62, 126.65, 129.11, 132.03, 134.85, 141.63, 142.18, 150.65, 151.55, 163.03, 163.09, 167.16, 167.51, 203.35. **HRMS** (DART) *m/z* 430.17475, calc. 430.17668 for [M+1].

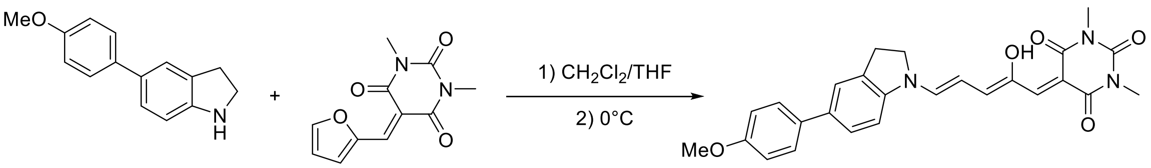

5-((2*Z*,4*E*)-2-hydroxy-5-(5-(4-methoxyphenyl)indolin-1-yl)penta-2,4-dien-1-ylidene)-1,3-dimethylpyrimidine-2,4,6(1*H*,3*H*,5*H*)-trione (5): To a solution of 190 mg of 5-(4-methoxyphenyl)indoline (2 ml of THF), 188 mg of activated furane dissolved in 5 ml CH_2Cl_2 were added. The reaction was stirred 24 h in a water bath at room temperature and in darkness. The solvent was evaporated, then the residue was suspended in 10 ml Et_2O and filtered. The filtered cake was washed with cold methanol. The crude was then purified by flash chromatography silica column ($CH_2Cl_2 \rightarrow$ 97:3 CH_2Cl_2:MeOH) to afford 65 mg (18%) of blue crystals. Open form: **^{1}H NMR** (700 MHz, CD_2Cl_2, 300 K) δ 3.30 (s, 3H), 3.33 (s, 3H), 3.36 (t, *J* = 7.9 Hz, 2H), 3.84 (s, 3H), 4.17 (t, *J* = 7.9, Hz, 2H), 6.21 (t, *J* = 11.7 Hz, 1H), 6.79 (d, *J* = 11.7 Hz, 1H), 6.98 (dt, *J* = 8.8, 2.2 Hz, 2H), 7.16 (m, 1H), 7.35 (s, 1H), 7.49 (ddq, *J* = 4.0, 1.9, 1.0 Hz, 2H), 7.52 (dt, *J* = 8.9, 2.2 Hz, 2H), 7.74 (d, *J* = 11.7 Hz, 1H), 12.42 (s, 1H). **^{13}C NMR** (176 MHz, CD_2Cl_2, 300 K) δ 27.92, 28.55, 28.68, 49.89, 55.74, 105.05, 110.33, 114.72, 124.38, 127.04, 128.15, 131.48, 132.98, 138.36, 141.56, 142.5 (determined by HSQC) 143.21, 146.0 (determined by HSQC) 153.95, 157.64, 159.82, 163.65, 165.73, 203.39.

Closed form: **^{1}H NMR** (700 MHz, CD_2Cl_2, 300 K) δ 3.05 (s, 3H), 3.08 (ddtd, *J* = 31.0, 25.8, 15.7, 9.0 Hz, 2H), 3.33 (s, 3H), 3.36 (q, *J* = 9.7, 8.3 Hz, 1H), 3.51 (q, *J* = 9.7, 8.3 Hz, 1H), 3.74 (dd, *J* = 4.2, 2.0 Hz, 1H), 3.82 (s, 3H), 4.08 (d, *J* = 2.0 Hz, 1H), 5.29 (dt, *J* = 4.2, 2.0 Hz, 1H), 6.41 (dd, *J* = 5.9, 2.2 Hz, 1H), 6.44 (d, *J* = 8.2 Hz, 1H), 6.93 (dt, *J* = 8.7, 2.1 Hz, 2H), 7.16 (dq, *J* = 8.2, 2.1, 1.5, 1.2 Hz, 1H), 7.31 (d, *J* = 1.5 Hz, 1H), 7.43 (dt, *J* = 8.7, 2.1 Hz, 2H), 7.71 (dd, *J* = 5.9, 2.1 Hz, 1H). **^{13}C NMR** (176 MHz, CD_2Cl_2, 300 K) δ 28.68, 28.86, 29.10, 47.56, 47.66, 47.98, 55.68, 60.98, 106.81, 114.53, 123.93, 125.88, 127.65, 131.87, 133.85, 134.24, 134.80, 150.13, 151.55, 158.95, 163.11, 167.17, 167.51, 203.39. **HRMS** (DART) *m/z* 460.18621, calc. 460.18725 for [M+1].

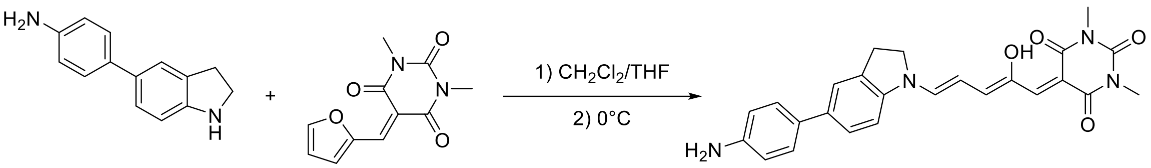

5-((2*Z*,4*E*)-5-(5-(4-aminophenyl)indolin-1-yl)-2-hydroxypenta-2,4-dien-1-ylidene)-1,3-dimethylpyrimidine-2,4,6(1*H*,3*H*,5*H*)-trione (6): To a solution of 150 mg 4-(indolin-5-yl)aniline dissolved in 3ml THF, was added 152 mg activated furan in 3 ml CH_2Cl_2. The reaction mixture was stirred in darkness in a water bath for 24 h. After this time, the solvent was evaporated and the residue was suspended in THF, cooled to 0 ºC and filtered. The crude obtained was purified by flash chromatography silica column (96:4 CH_2Cl_2:MeOH) to yield 45 mg (15%) of a dark blue powder. Open form: **^{1}H NMR** (700 MHz, CD_2Cl_2, 300 K) δ 3.30 (s, 3H), 3.33 (s, 3H), 3.35 (t, *J* = 8.4 Hz, 2H), 4.16 (t, *J* = 8.4 Hz, 2H), 6.21 (t, *J* = 11.5 Hz, 1H), 6.74 (d, *J* = 8.4 Hz, 2H), 6.80 (d, *J* = 11.5 Hz, 1H), 7.15 (dd, J = 8.3, 1.4 Hz, 1H), 7.32 (s, 1H), 7.38 (d, J = 8.4 Hz, 2H), 7.46 (dt, *J* = 3.3, 1.7 Hz, 2H), 7.74 (d, *J* = 11.5 Hz, 1H), 12.44 (s, 1H). **^{13}C NMR** (176 MHz, CD_2Cl_2, 300 K) δ 27.93, 28.67, 28.86, 49.92, 105.09, 110.37, 115.63, 123.04, 123.90, 125.44, 127.40, 127.97, 129.14, 132.42, 142.68, 143.02, 146.21, 148.42, 150.76, 151.95, 163.14, 165.71, 167.99.

Closed form: **^{1}H NMR** (700 MHz, CD_2Cl_2, 300 K) δ 3.05 (s, 3H), 3.07 (dtd, *J* = 24.8, 15.7, 14.5, 8.3 Hz, 2H), 3.33 (s, 5H), 3.35 (q, *J* = 9.7, 8.3 Hz, 1H), 3.49 (q, *J* = 9.7, 8.3 Hz, 1H), 3.74 (dd, *J* =

4.0, 1.5 Hz, 1H), 4.08 (d, *J* = 2.1 Hz, 1H), 5.28 (td, *J* = 4.0, 2.0 Hz, 1H), 6.41 (dd, *J* = 6.1, 2.1 Hz, 1H), 6.42 (d, *J* = 8.2 Hz, 1H), 6.71 (d, *J* = 8.5 Hz, 2H), 7.13 (dd, *J* = 8.2, 2.0 Hz, 1H), 7.30 (d, *J* = 8.5 Hz, 2H), 7.71 (*J* =6.1, 2.0 Hz, 1H, 1H). **^{13}C NMR** (176 MHz, CD_2Cl_2, 300 K) δ 28.53, 28.72, 29.09, 47.57, 47.63, 47.97, 61.04, 106.84, 115.61, 123.62, 126.58, 127.50, 130.33, 131.41, 131.85, 134.76, 145.83, 149.78, 151.55, 163.19, 167.18, 167.50, 203.44. **HRMS** (DART) *m/z* 445.18596, calc. 445.18758 for [M+1].

Determination of the open-closed isomer proportions

The relative populations of the two isomers in **DASA** solutions were determined by integration of specific sets of peaks assigned to each isomer in freshly prepared solutions. The relative proportions of the two isomers was used to determine the absorption coefficient at the visible band maximum (open isomer) which is required to quantify the two-photon conversion properties of molecules **4-6** as explained in the main text. Peak assignments were made from 2D (COSY) and ^{1}H-NMR experiments acquired in order to determine which peaks increase (closed form) and which diminish (open form) when the sample is irradiated with visible light. The full set of spectra are included in the respective section of this Supporting Information file.

DASA 4

Integration of the peaks at 6.20 and 6.80 ppm (open isomer): 2.09. Integration of peaks at 6.42 and 6.46 ppm (closed isomer): 0.90. Ratio open:closed 2.09:0.9 = 69% open isomer, 31% closed isomer. The spectra correspond to Figure S1 and S2 for the integrals of the open and closed isomers respectively.

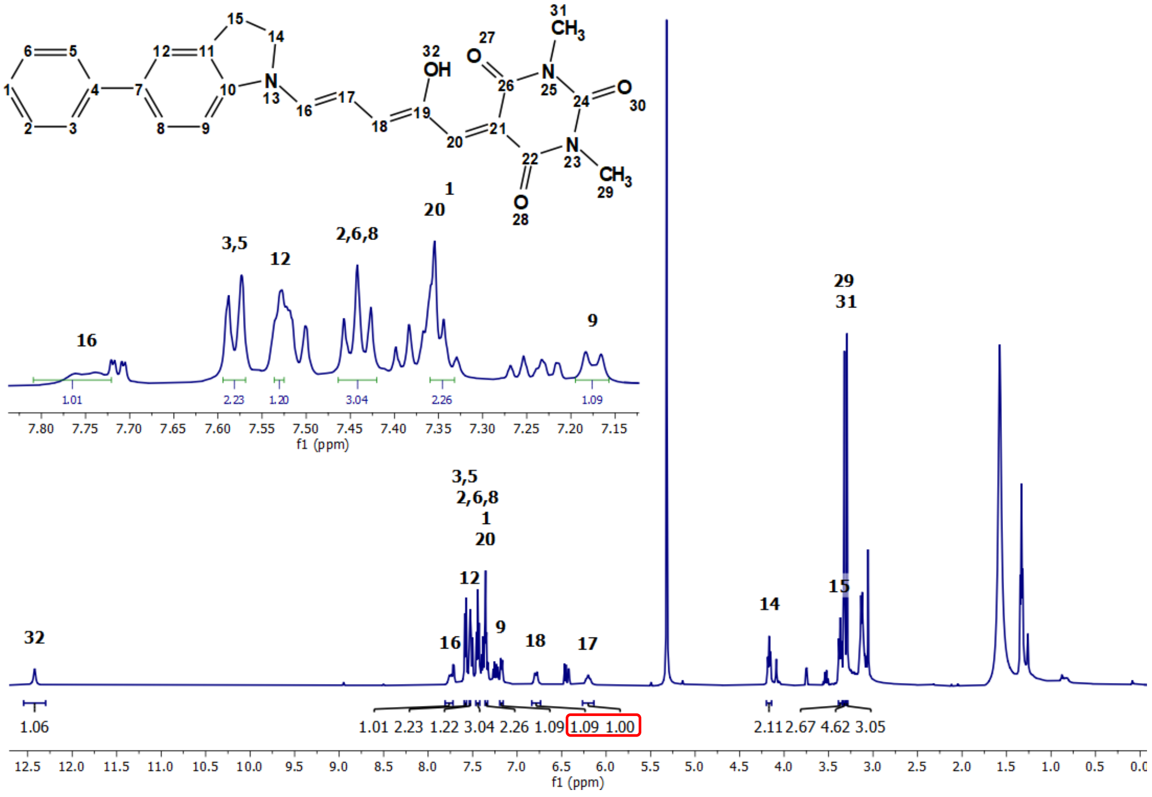

Figure S1. ^{1}H-NMR spectrum of **4** in fresh solutions in CD_2Cl_2. Red box: Signals used for integration for the open form isomer used to determine relative populations.

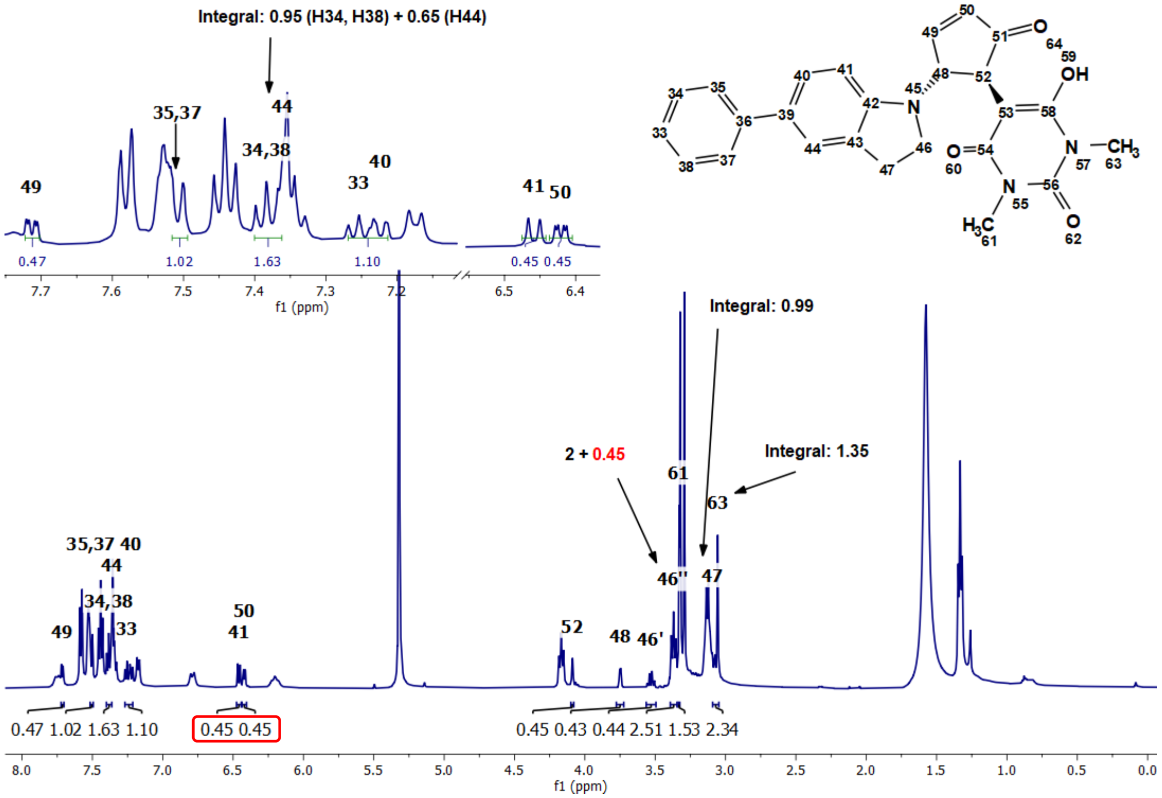

Figure S2. ^{1}H-NMR spectrum of **4** in fresh solutions in CD_2Cl_2**.** Red box: Peak integrations for the closed form isomer used to the determine relative populations. It should be mentioned that the signal from H15 of the open form is mixed with the signal from H46 of the closed form.

DASA 5

Integration for peaks at 6.20 and 6.80 ppm (open isomer): 1.95. Integration of both peaks at 6.41 and 6.43 ppm (closed isomer): 0.87. Ratio open:closed 1.95:0.87 = 69% open isomer, 31% closed isomer. The spectra correspond to Figure S3 and S4 for the integrals of the open and closed isomers respectively.

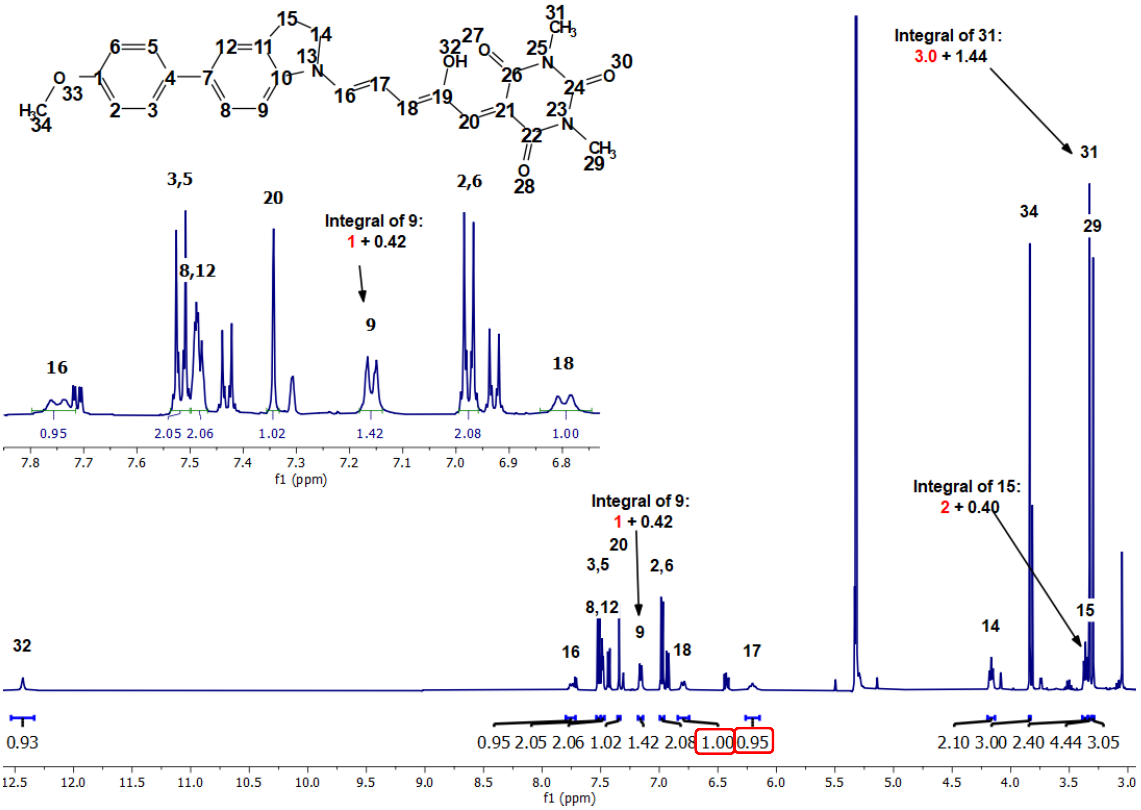

Figure S3. ^{1}H-NMR spectrum of **5** in fresh solutions in CD_2Cl_2. Red box: Signals integration for the open form isomer used to determine relative populations. It should be noted that H15 and H31 (red) are mixed with peaks of H42, H48" and H65, respectively from the closed isomer

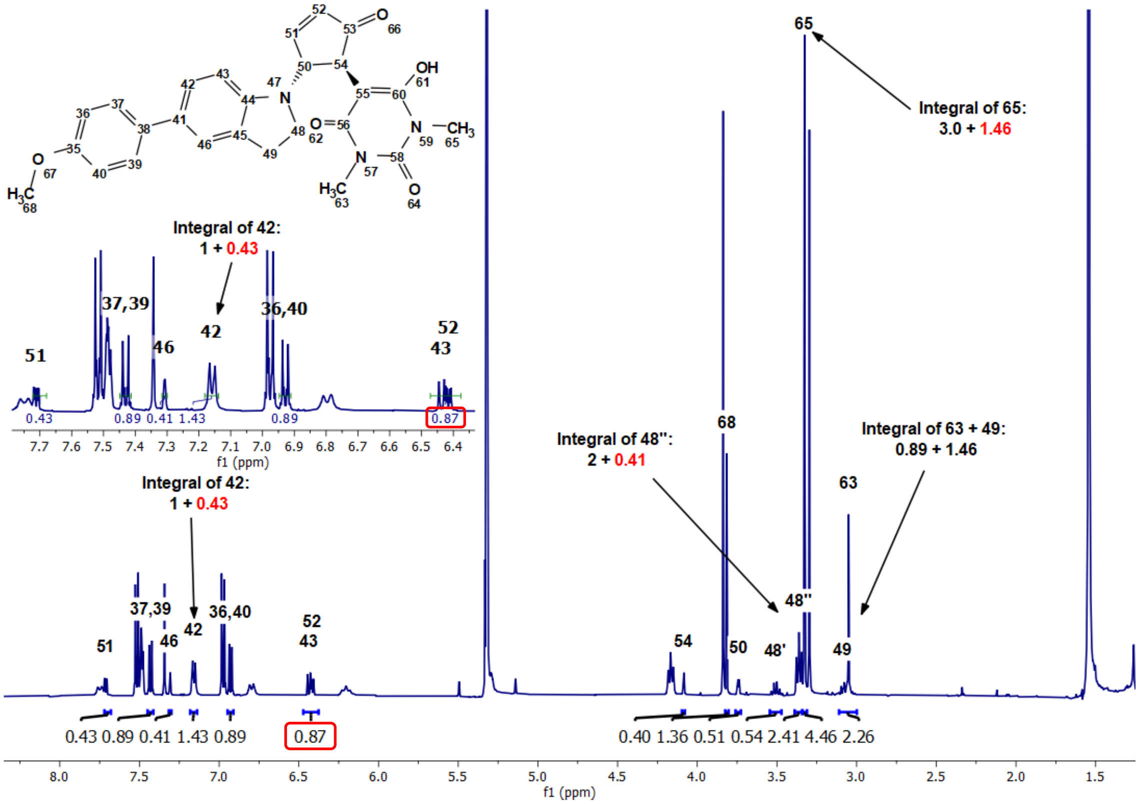

Figure S4. ^{1}H-NMR spectrum of **5** in fresh solutions in CD_2Cl_2. in closed isomer. Red box: Signal integration for closed form isomer used to determine relative populations. It should be noted that H42, H15 and H31 (in red) are mixed with peaks for H9, H15 and H31, respectively, from the open isomer.

DASA 6

Integration for both peaks at 6.20 and 6.80 ppm (open isomer): 0.99. Integration of both peaks at 6.41 and 6.43 ppm (closed isomer): 2.0. Ratio open:closed 0.99:2.0 = 33% open isomer, 66% closed isomer. The spectra correspond to Figure S5 and S6 for the integrals of the open and closed isomers respectively.

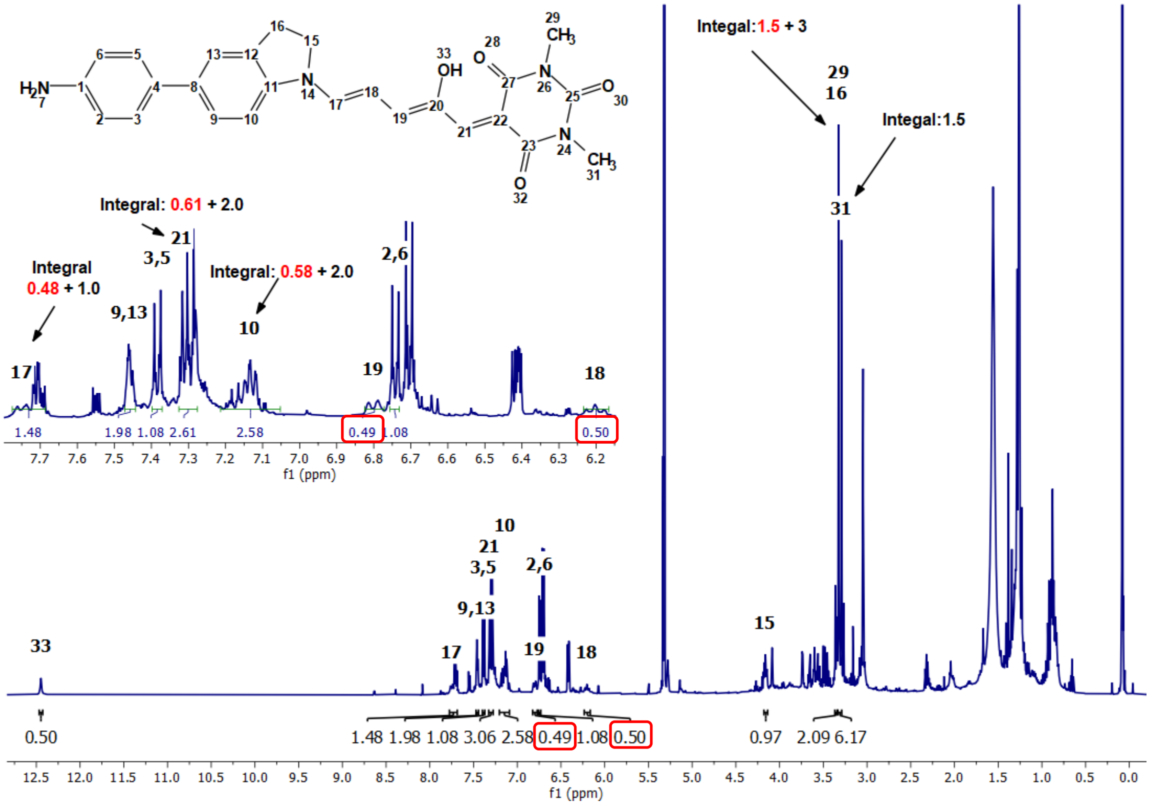

Figure S5. ^{1}H-NMR spectrum of **6** in fresh solutions in CD_2Cl_2. Red box: Signal integration for the open form isomer used to determine the relative populations. It should be noted that H29, H10, H21 and H17 (red) are mixed with the peaks of H62, H42 and 46, H36 and 38, and H52, respectively from the closed isomer

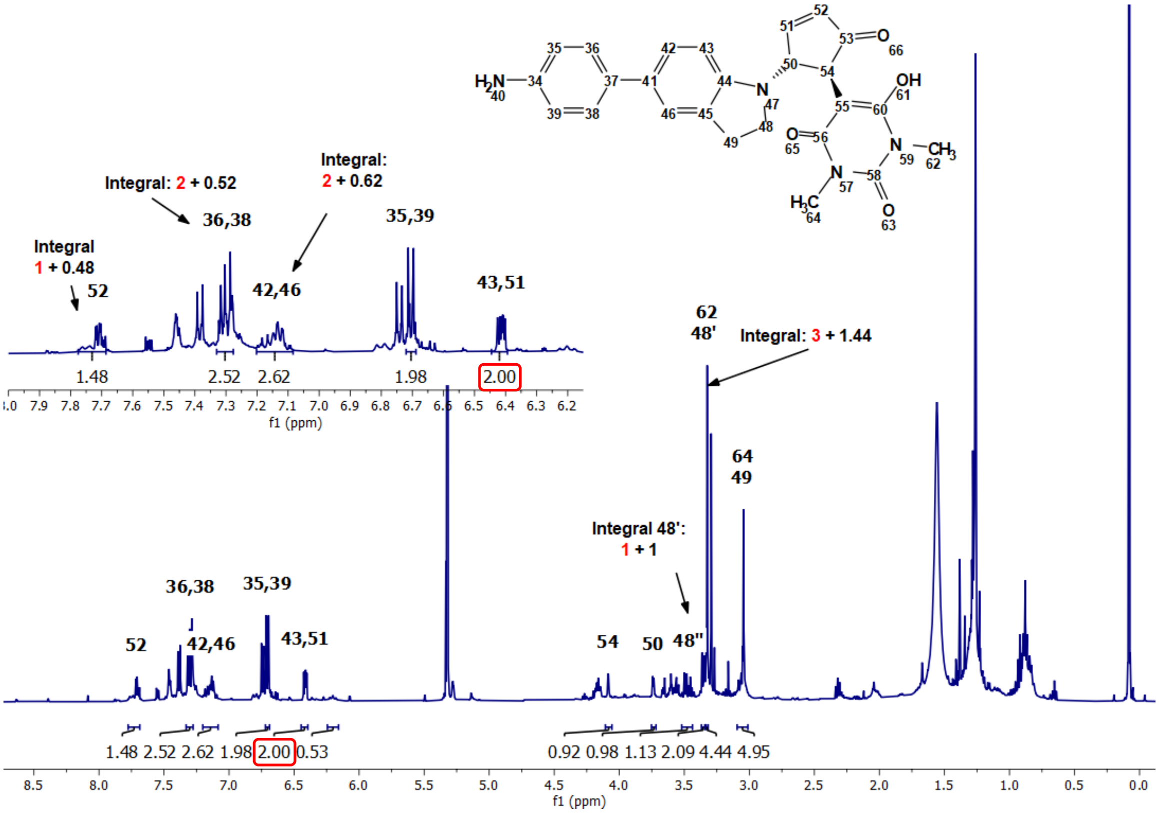

Figure S6. ^{1}H-NMR spectrum of **6** in fresh solutions in CD_2Cl_2. Red box: Signal integration of closed form isomer used to determine relative populations. It should be mentioned that H62, H42 and 46, H36 and 38, and H52(red) are mixed with the peaks of H29, H10, H21 and H17 respectively from the closed isomer.

Steady-state spectroscopy

Molar absorptivity coefficients ε(λ)

The equation used for the determination of ε(λ) of DASAs **4** to **6** for each isomer is:

$$\frac{A}{l \cdot C_{Total}} - \varepsilon_c(1 - x_o) = \varepsilon_o x_o$$

Where:

A= total initial absorbance at each wavelength.

C_{Total}= total concentration of each DASA.

ε_c and ε_o are the molar absorptivity coefficients of closed and open isomers at each wavelength.

x_o= mol fraction of the open isomer.

Due to the presence of both open and close isomers in the solutions of the DASAs of this study, in order to determine ε_o, we first measured the UV-Vis spectra of the samples of known total concentration in toluene. Then, we fully bleached the absorbance of the visible band (open form) with visible light to obtain a sample composed only of the closed isomer. This allowed a direct measure of ε_c at each wavelength. The calculation for ε_o was made taking the open mole fractions from the NMR data and the equation above.

Table S1. Absorption coefficients at the first band maxima for molecules **4** to **6.**

DASA	ε_O at Abs_{max}($M^{-1}cm^{-1}$)
**4**	201.5 x10^3
**5**	188.5 x10^3
**6**	124.2 x10^3

DASA 4

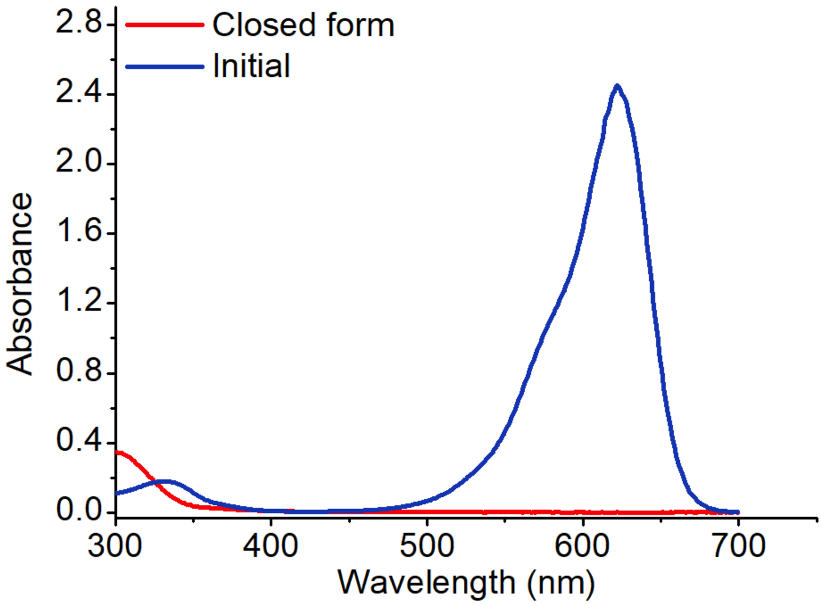

Figure S7. Absorption spectra an initial sample (blue) and a sample fully photo-converted to the closed form (red) for DASA **4** in toluene. Total initial concentration: 18 μM.

DASA 5

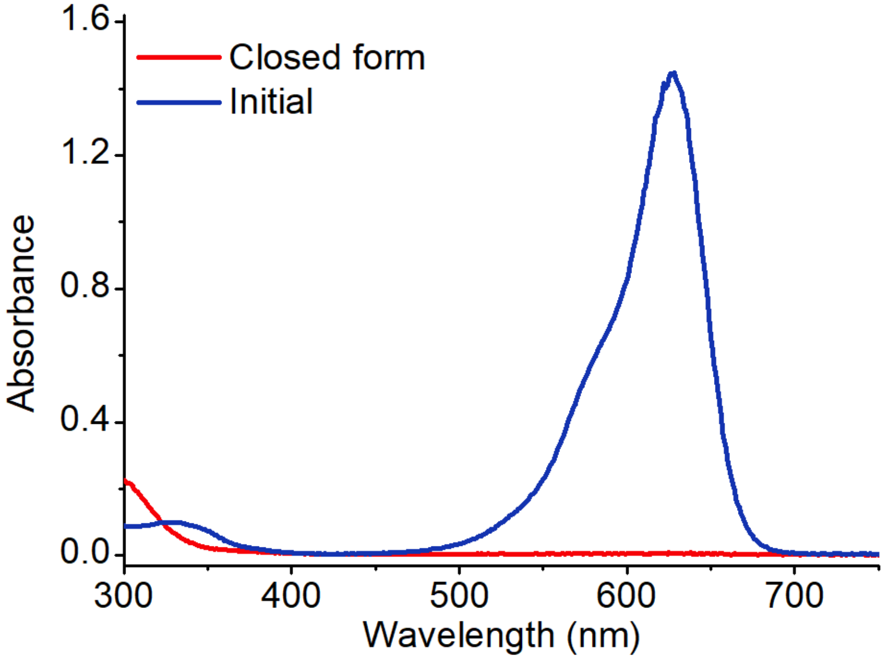

Figure S8. Absorption spectra of an initial sample (blue) and sample fully photo-converted to the closed form (red) for DASA **5** in toluene. Total initial concentration: 11 μM.

DASA 6

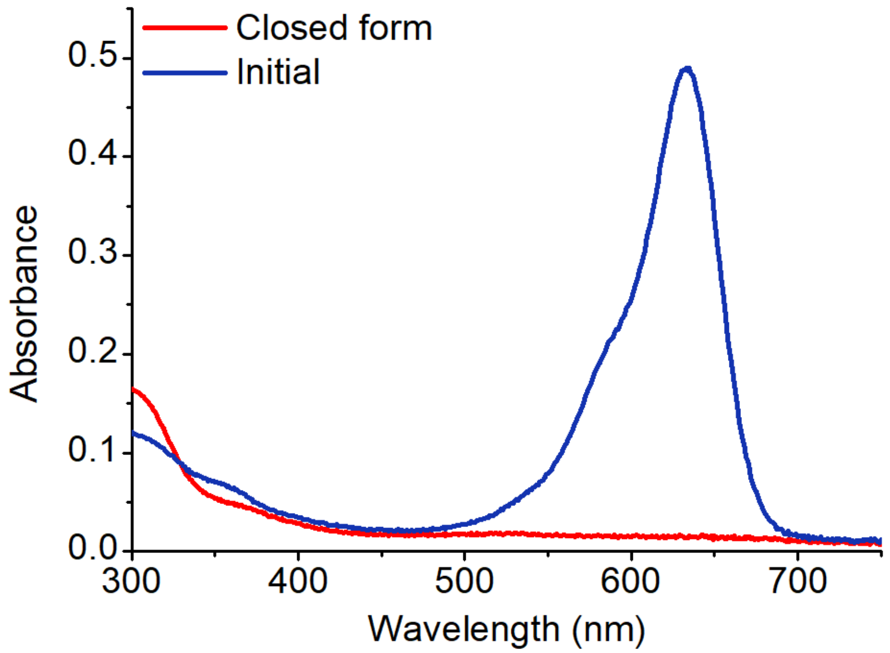

Figure S9. Absorption spectra of thermally equilibrated sample (blue) and sample fully photo-converted to the closed form (red) for DASA **6** in toluene. Total initial concentration: 11.7 μM.

Linear photochemistry

Photoisomerization quantum yields

Absorption data after linear excitation of DASA solutions were fitted according to the following equation. The yield was obtained from the slope after a linear regressions.

$$N_{isom} = \varphi_{isom}(Ph_{abs})$$

Where:

N_{isom}: number of molecules photo-isomerized in the sample.

Ph_{abs}: photons absorbed by the sample.

The equation was used in the following form:

$$\frac{\Delta Abs}{\varepsilon l} \cdot N_A \cdot V = \phi_{isom} \frac{P \cdot t}{E_{632nm}} \cdot \left(1 - 10^{-Abs}\right)$$

Where:

P= average beam intensity

t= irradiation time

E_{632nm}= energy of a 632.8 nm photon

Abs= average absorption of sample during irradiation time at 632.8 nm.

φ_{Isom}= isomerization quantum yield

ΔAbs= absorbance change at Abs_{max}

N_A= Avogadro's constant

V= sample volume

ε= molar absorptivity coefficient of the open form at the absorption maxima

The samples in toluene were irradiated with a 632.8 nm laser beam with 0.5 to 0.25 mW power for different time lapses. For each irradiation time, a fresh sample was used.

Table S2. Isomerization quantum yields for the DASAs of the present study

DASA	Isomerization Quantum Yield
2	0.52
3	0.30
4	0.15
5	0.17
6	0.25

DASA 2

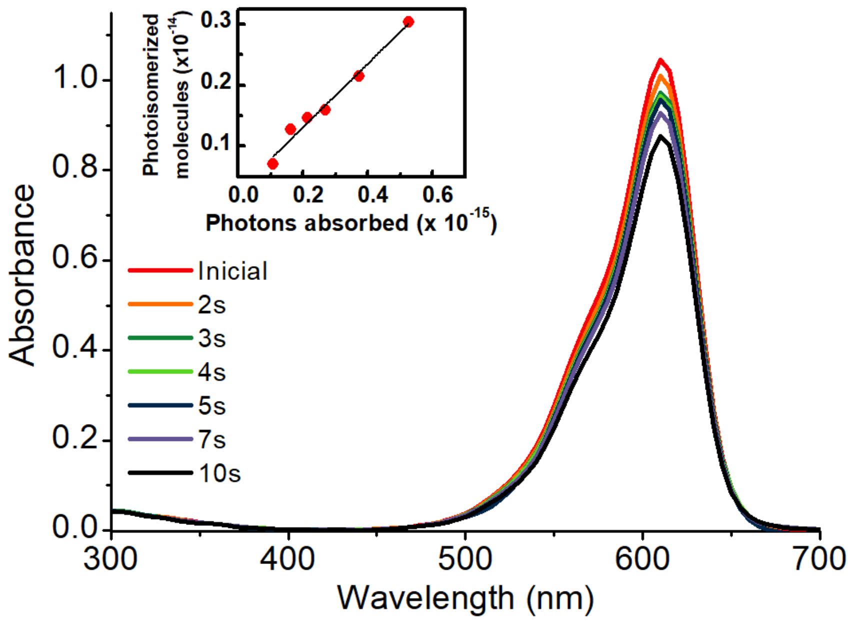

Figure S10. Changes in the absorption spectra of DASA **2** with different 632.8 nm irradiation times. Inset: Linear fit. Slope: 0.52 Intercept: $2.7x10^{-14}$, R^2: 0.98. Sample conditions: Light power: 0.25 mW, sample volume: 3 ml.

DASA 3

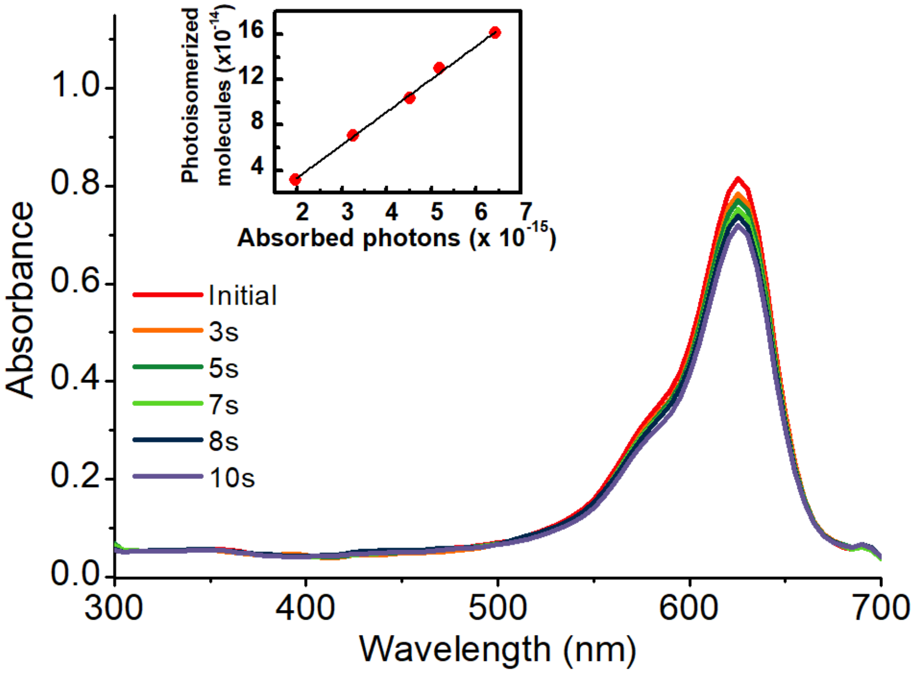

Figure S11. Changes in the absorption spectra of DASA **3** with different 632.8 nm irradiation times. Inset: Linear fit. Slope: 0.30, Intercept: $-1.99x10^{14}$, R^2: 0.99. Sample conditions: Light power: 0.25 mW, sample volume: 3 ml.

DASA 4

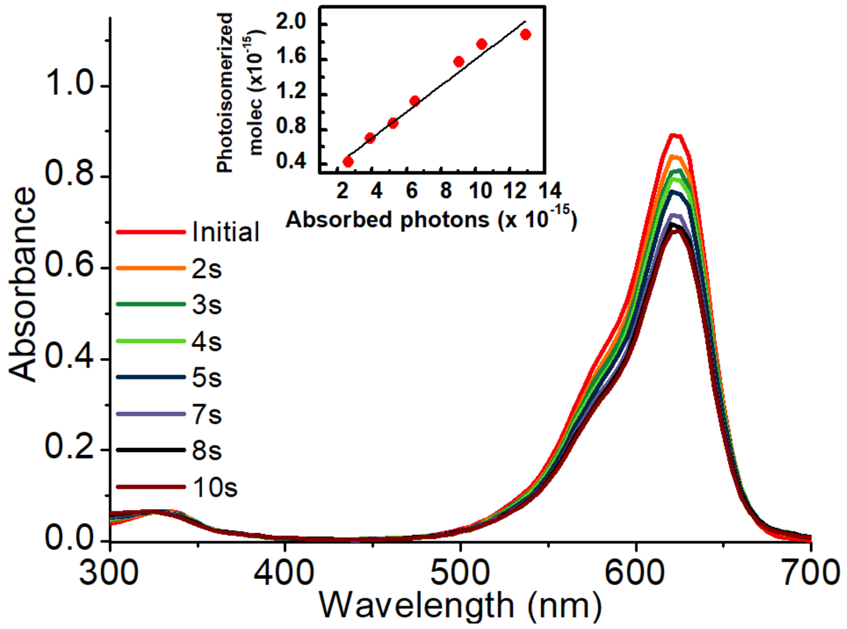

Figure S12. Changes in the absorption spectra of DASA **4** with different 632.8 nm irradiation times. Inset: Linear fit. Slope: 0.15 Intercept: $1.02 x 10^{14}$, R^2: 0.96. Sample conditions: Light power: 0.50 mW, sample volume=3ml.

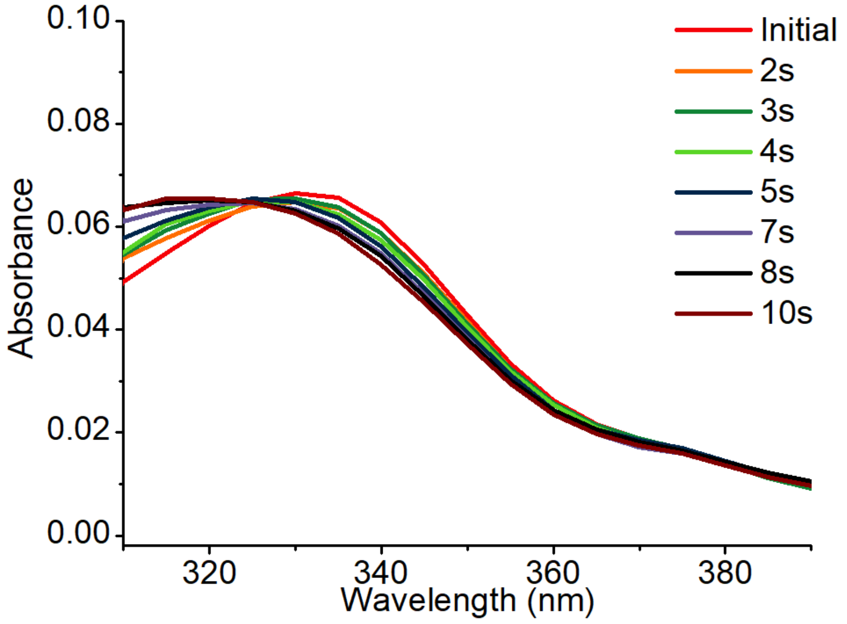

Figure S13. Closer look of the spectra presented in Figure S12 in the isosbestic point region (~325 nm).

DASA 5

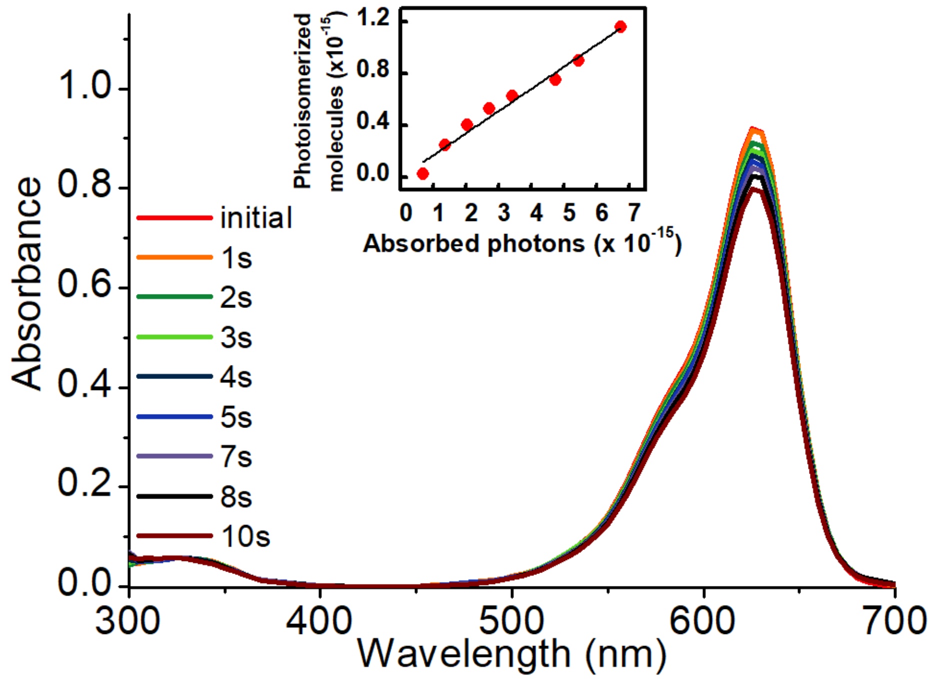

Figure S14. Changes in the absorption spectra of DASA **5** with different 632.8 nm irradiation times. Inset: Linear fit. Slope: 0.17, Intercept: -2.20x10^{12}, R^2: 0.97. Sample conditions: Average 632.8 nm power: 0.25 mW, sample volume: 3 ml

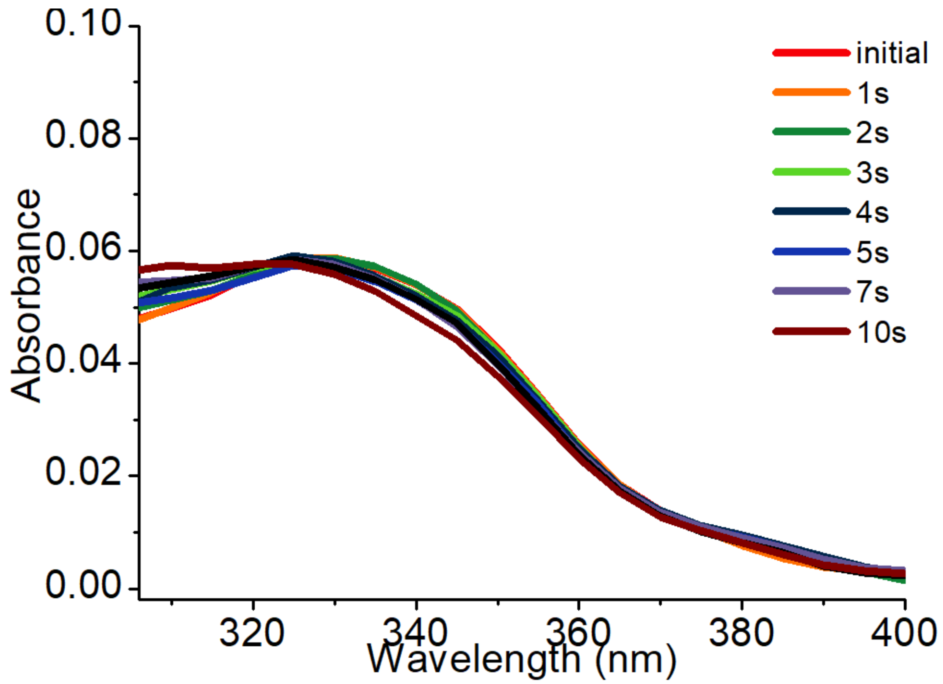

Figure S15. Closer look of the spectra presented in Figure S14 in the isosbestic point region (~325 nm).

DASA 6

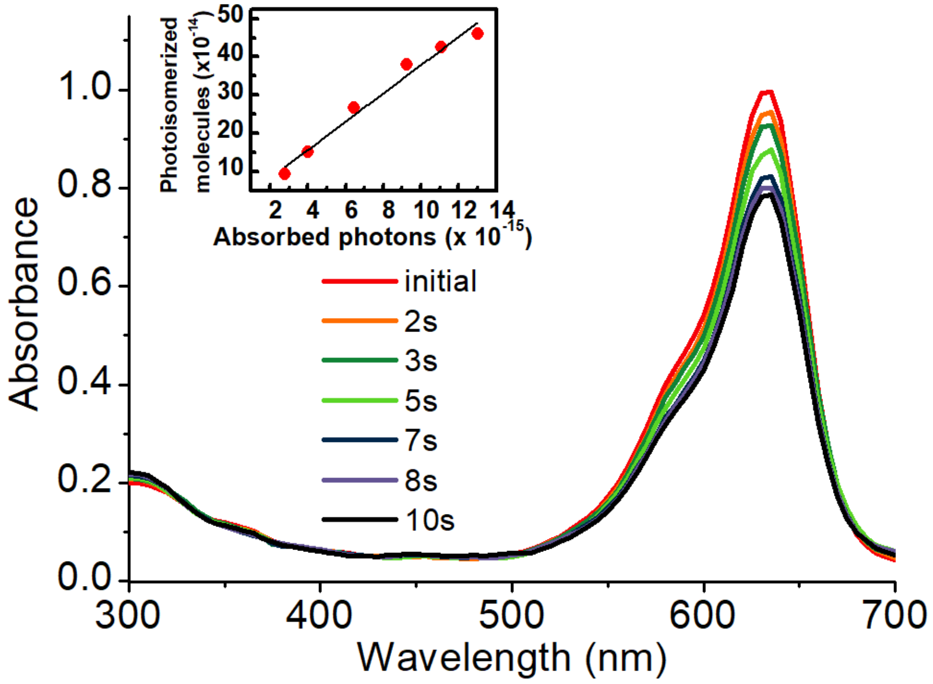

Figure S16. Changes in absorption spectra of **6** with different 632.8 nm irradiation times. Inset: Linear fit. Slope: 0.24, Intercept: $5.53x10^{13}$, R^2: 0.97. Sample conditions: 0.5 mW, sample volume: 3 ml.

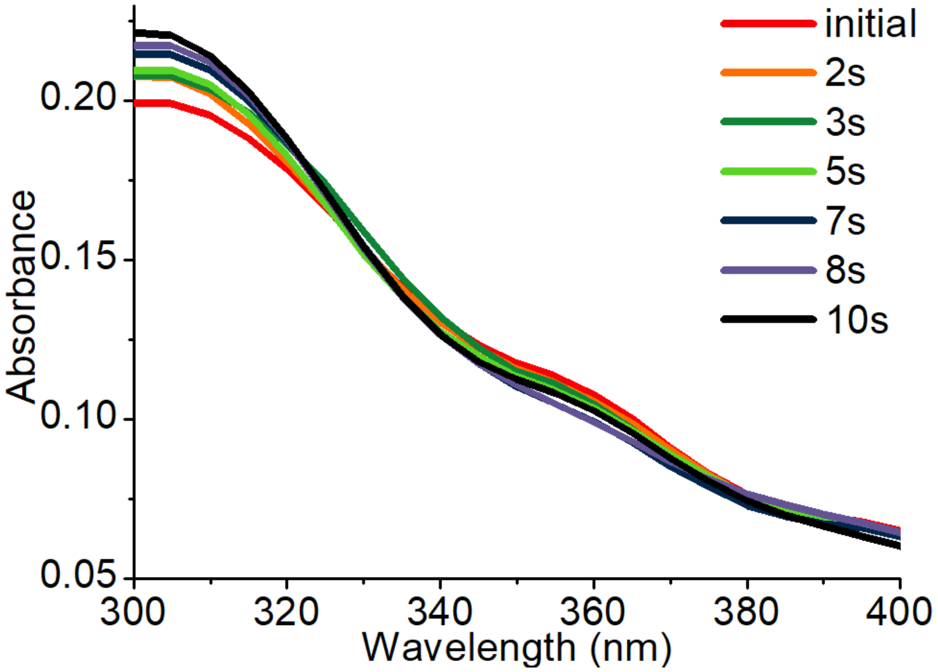

Figure S17. Closer look of the spectra presented in Figure S16 in the isosbestic point region (~325 nm).

Two-photon chemistry

Intensity dependence of the photoisomerizations

DASA 5

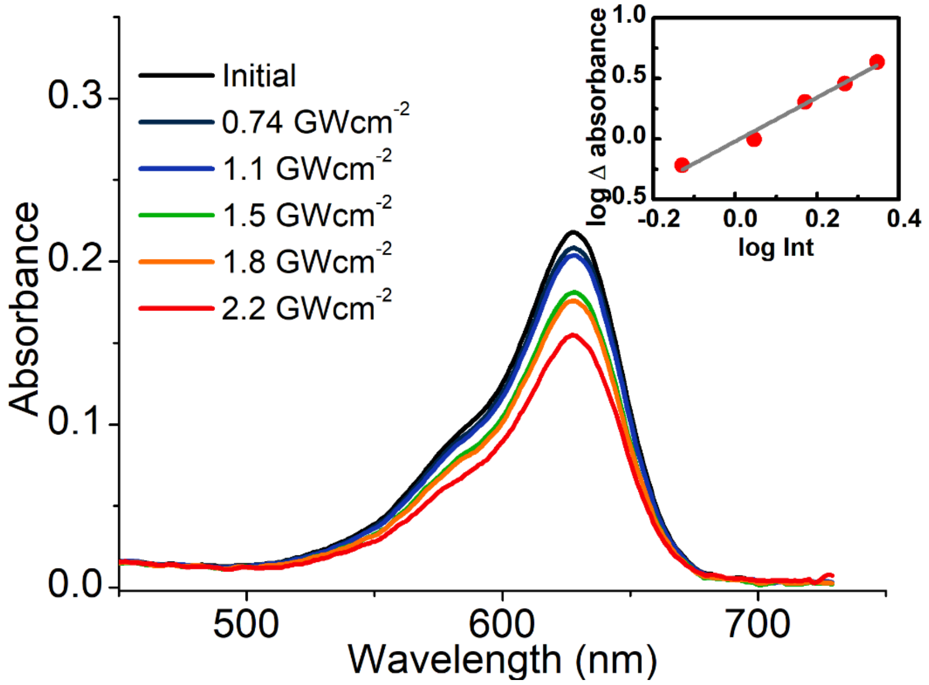

Figure S18. Change in absorption bands for DASA **5** at different 800 nm pulsed beam intensities. Inset: linear fit of absorbance change *vs* pulse peak intensities, slope: 1.8, intercept: -0.019, R^2: 0.98. Sample conditions: irradiation time: 6 min, sample volume:1.5 ml.

DASA 6

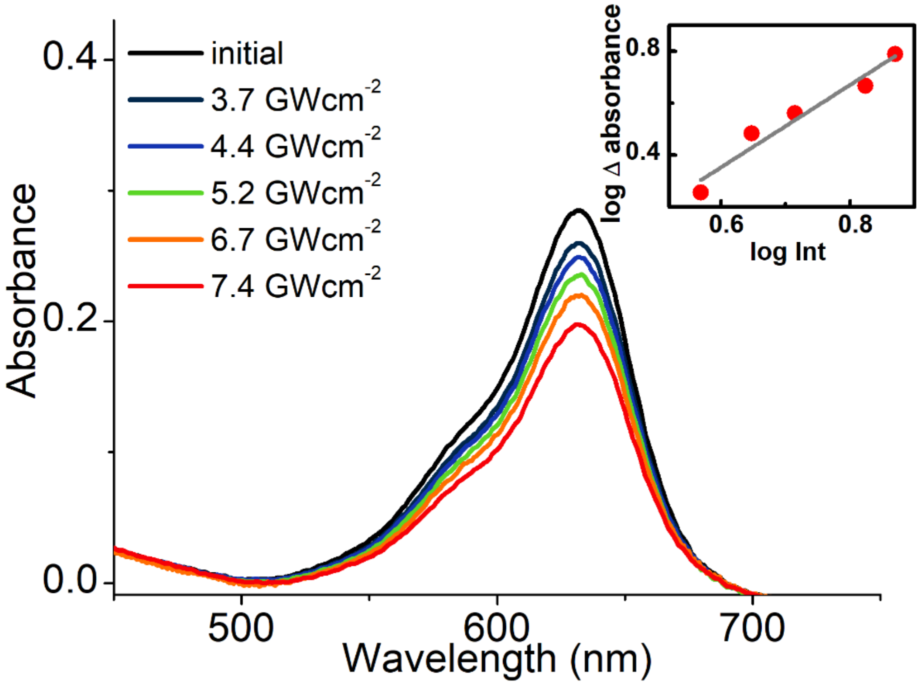

Figure S19. Change in absorption bands for DASA **6** at different 800 nm pulsed beam intensities. Inset: linear fit of absorbance change *vs* pulse peak intensities, slope: 1.6, intercept: -0.06, R^2: 0.93. Sample conditions: irradiation time: 3 min, sample volume:1.5 ml.

Spectral evolutions during 800 nm pulsed irradiations

DASA 3

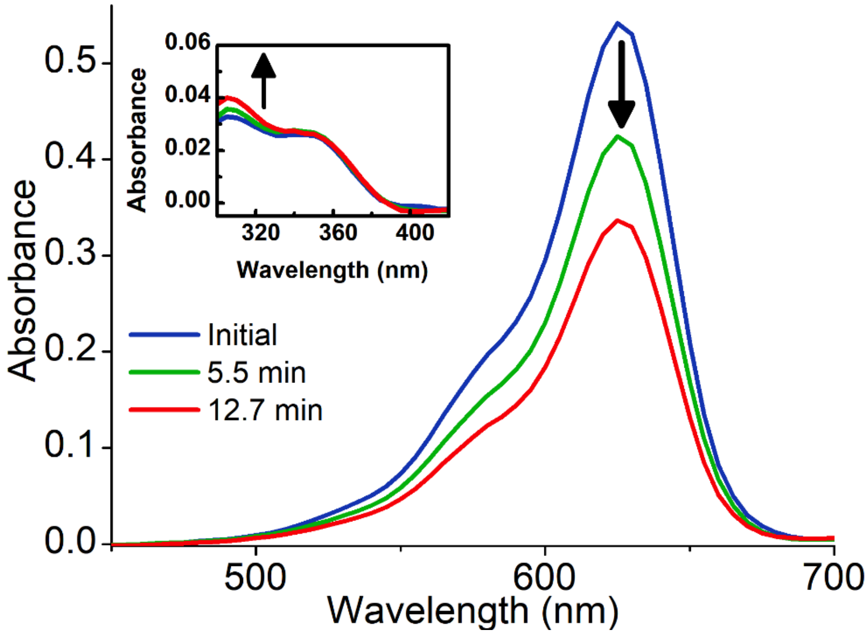

Figure S20. Absorption spectra of DASA **3** during biphotonic 800 nm excitation. The inset shows the isosbestic point in the 325 nm region.

DASA 4

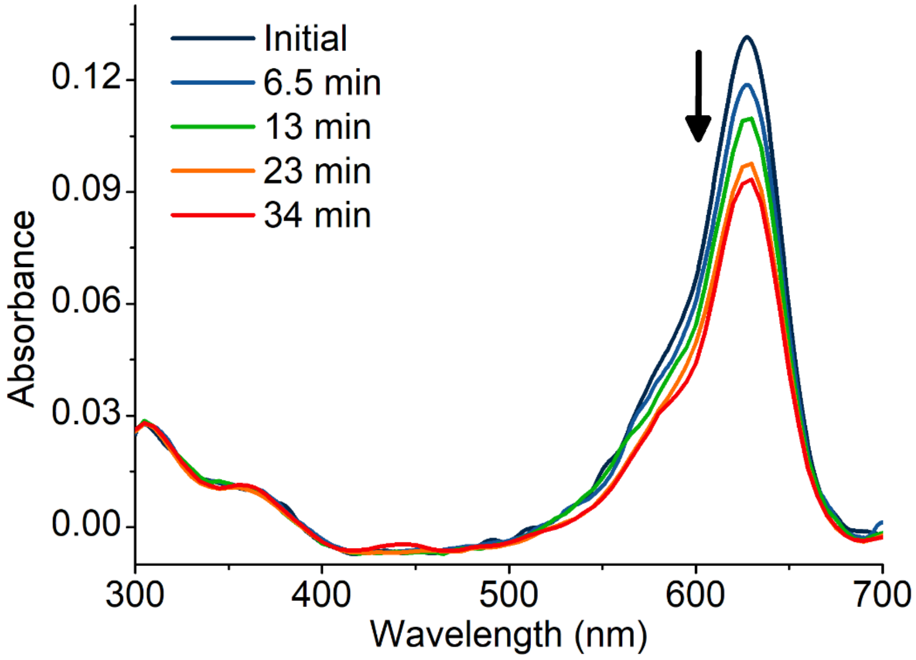

Figure S21. Absorption spectra of DASA **4** during biphotonic 800 nm excitation. For this DASA, the isosbestic region corresponds to wavelengths below 400 nm.

DASA 6

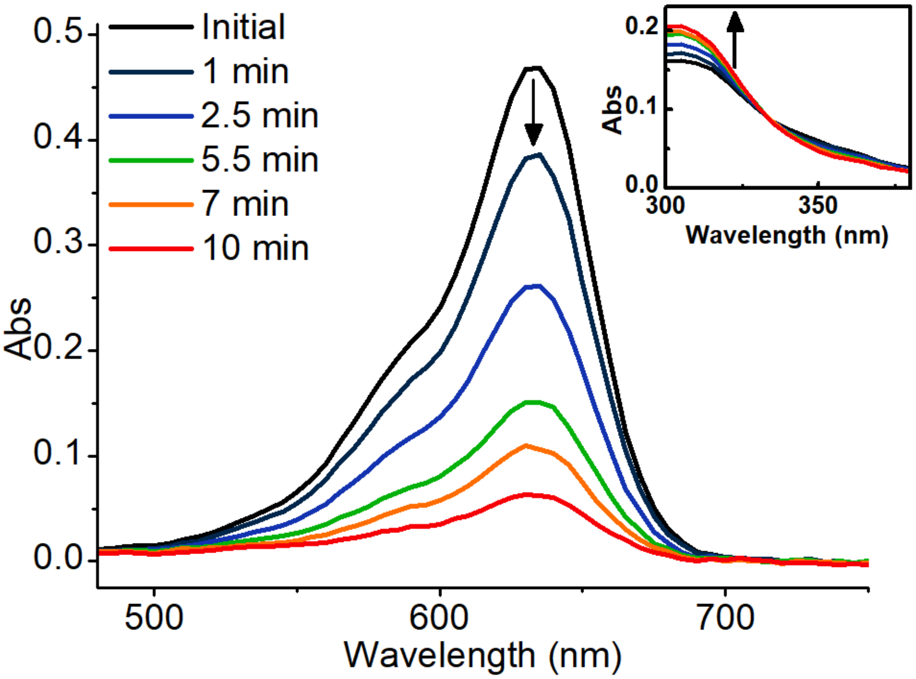

Figure S22. Absorption spectra of DASA **6** during biphotonic 800 nm excitation. The inset shows the isosbestic point in the 325 nm region.

Thermal equilibrations after 800 nm pulsed irradiations

DASA 4

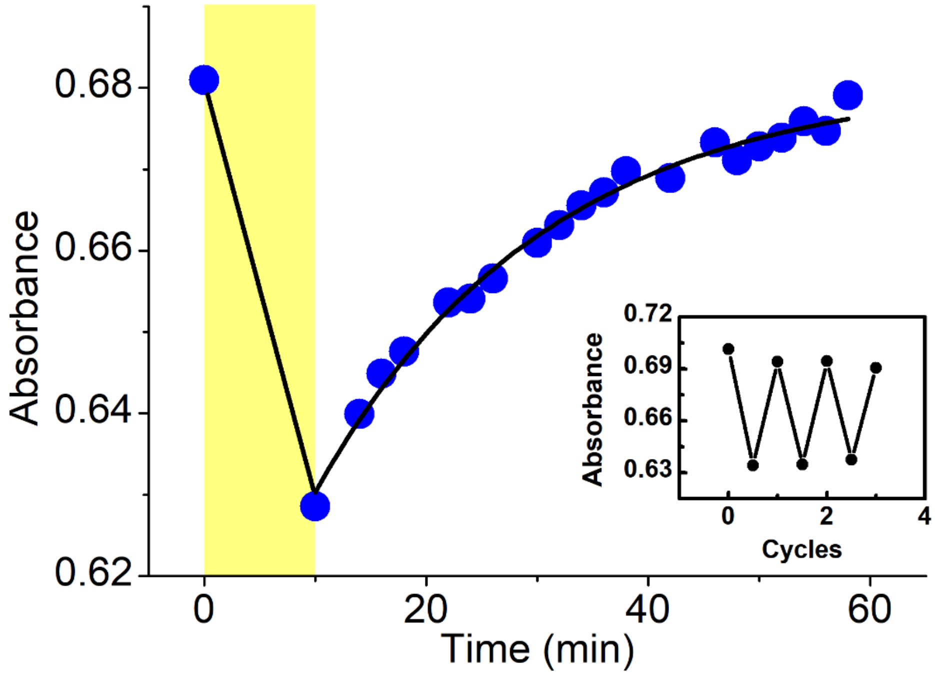

Figure S23. Biphotonic bleaching of DASA **4** measured at 629 nm and thermal return in chlorobenzene. The thermal return was fitted to an exponential model $A = A_o e^{-kt}$: A_o=-0.08, k= 0.0442 min^{-1}, R^2= 0.98 ; yellow zone: irradiation period. Inset: fatigue sequence of thermal returns.

DASA 6

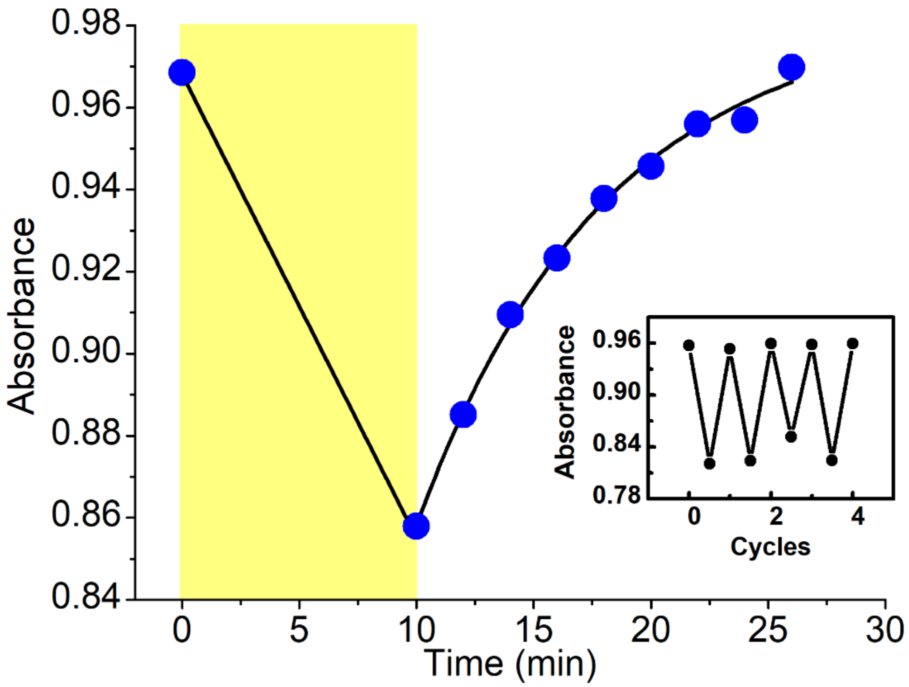

Figure S24. Biphotonic bleaching of DASA **6** measured at 629 nm, and thermal return in chlorobenzene. The thermal return was fitted to an exponential model $A = A_o e^{-kt}$: A_o=-0.43, k= 0.043 min^{-1}, R^2= 0.9 ; yellow zone: irradiation period. Inset: fatigue sequence of thermal returns.

Two-photon absorption cross section measurements

Data obtained from the biphotonic intensity dependence was fitted according to the following equation to measure the TPA-cross section σ_2 as described in the main text:

$$ln\left(\frac{N_g}{N_o}\right) = -\frac{1}{2}\sigma_2\phi^2\tau$$

DASA 3

Table S3. Data for the calculation of σ_2 of DASA **3.** The last column shows the σ_2 value considering the specific average power in the row.

Average pulse train power (W)	Initial absorbance at maximum	Δ Abs	$-0.5\varphi^2\tau$ x10^{-50}	$\ln(N_g/N_o)$	σ_2 / GM
0.06	0.361	0.0187	-1.06E-07	-8.50E-06	80.2
0.08	0.383	0.0290	-1.88E-07	-1.24E-05	65.9
0.1	0.389	0.0515	-2.95E-07	-2.17E-05	73.7
0.12	0.394	0.0653	-4.24E-07	-2.72E-05	64.0
0.14	0.413	0.0925	-5.77E-07	-3.67E-05	63.6

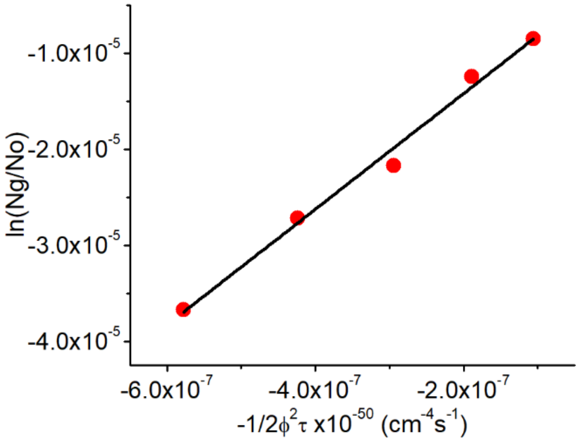

Figure S25. Excitation intensity dependence of the photoconversion of DASA **3**. Slope: 60.2 GM, intercept: -2x10^{-6}, R^2=0.98 Sample conditions: Volume: 1.5 ml, Irradiation time: 300 s.

DASA 4

Table S4. Data for the calculation of σ_2 of DASA **4.** The last column shows the σ_2 value considering the specific average power in the row

Average pulse train power (W)	Initial absorbance at maximum	Δ Abs	$-0.5\varphi^2\tau$ x10^{-50}	$\ln(N_g/N_o)$	σ^2 / GM
0.03	0.611	0.0233	-2.65E-08	-1.25E-05	470.3
0.04	0.606	0.0367	-4.71E-08	-1.98E-05	419.6
0.05	0.604	0.0460	-7.36E-08	-2.48E-05	337.2
0.06	0.613	0.0807	-1.06E-07	-4.30E-05	405.2
0.07	0.609	0.0978	-1.44E-07	-5.25E-05	363.4

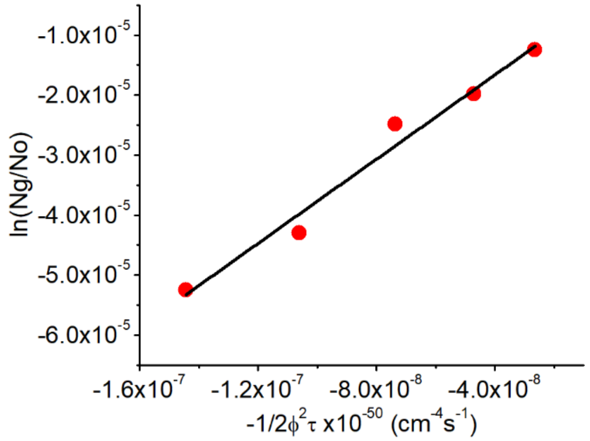

Figure S26. Excitation intensity dependence of the photoconversion of DASA **4**. Slope: 351.2 GM, intercept: -3x10^{-6}. R^2=0.97 Sample conditions: Volume: 1.5 mL, Irradiation time: 300 s.

DASA 5

Table S5. Data for the calculation of σ_2 of DASA **5.** The last column shows the σ_2 value considering the specific average power in the row

Average pulse train power (W)	Initial absorbance at maximum	Δ Abs	$-0.5\varphi^2\tau$ x10^{-50}	$\ln(N_g/N_o)$	σ_2 / GM
0.02	0.217	0.0078	-1.18E-08	-8.70E-06	737.9
0.03	0.217	0.0135	-2.65E-08	-1.49E-05	561.5
0.04	0.216	0.0282	-4.71E-08	-3.12E-05	663.5
0.05	0.224	0.0408	-7.36E-08	-4.37E-05	593.7
0.06	0.219	0.0607	-1.06E-07	-6.65E-05	626.8

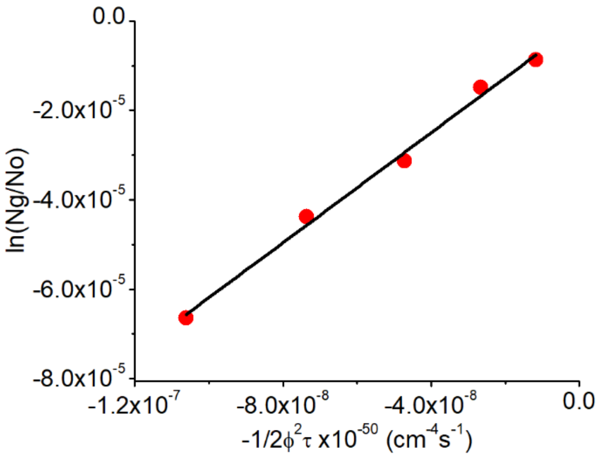

Figure S27. Excitation power dependence of the photoconversion of DASA **5**. Slope: 614.7 GM, intercept: -4x10^{-7}. R^2=0.99. Sample conditions: Volume: 1.5 ml , Irradiation time: 360 s.

DASA 6

Table S6. Data for the calculation of σ_2 of DASA **6.** The last column shows the σ_2 value considering the specific average power in the row

Average pulse train energy (W)	Initial absorbance at maximum	Δ abs	$-0.5\varphi^2\tau$ x10^{-50}	$\ln(N_g/N_o)$	σ^2 / GM
0.1	0.326	0.0245	-2.94E-07	-5.01E-05	170.2
0.12	0.328	0.0381	-4.24E-07	-7.75E-05	182.8
0.14	0.325	0.0482	-5.77E-07	-9.90E-05	171.3
0.18	0.327	0.0657	-9.54E-07	-1.34E-04	140.2
0.2	0.332	0.0899	-1.18E-06	-1.80E-04	153.0

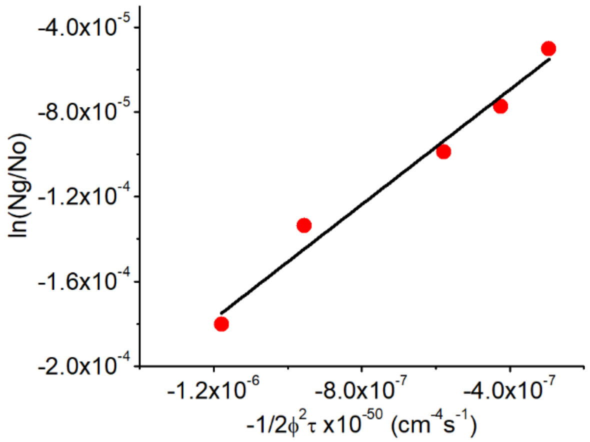

Figure S28. Excitation power dependence of the photoconversion of DASA **6**. Slope: 135.3 GM, intercept: -2x10^{-5}. R^2=0.97. Sample conditions: Volume: 3 mL, Irradiation time: 180 s.

Computational results

Table S7: Value of the range separation parameter μ obtained by imposing the Janak's Theorem on the OT-CAM-B3LYP functional ($\alpha = 0.0$, $\beta = 1.0$) using the def2-SVP basis. The data correspond to the structure associated with the global minimum.

System	μ
**1**	0.083
**2**	0.074
**3**	0.069
**4**	0.069
**5**	0.066
**6**	0.063

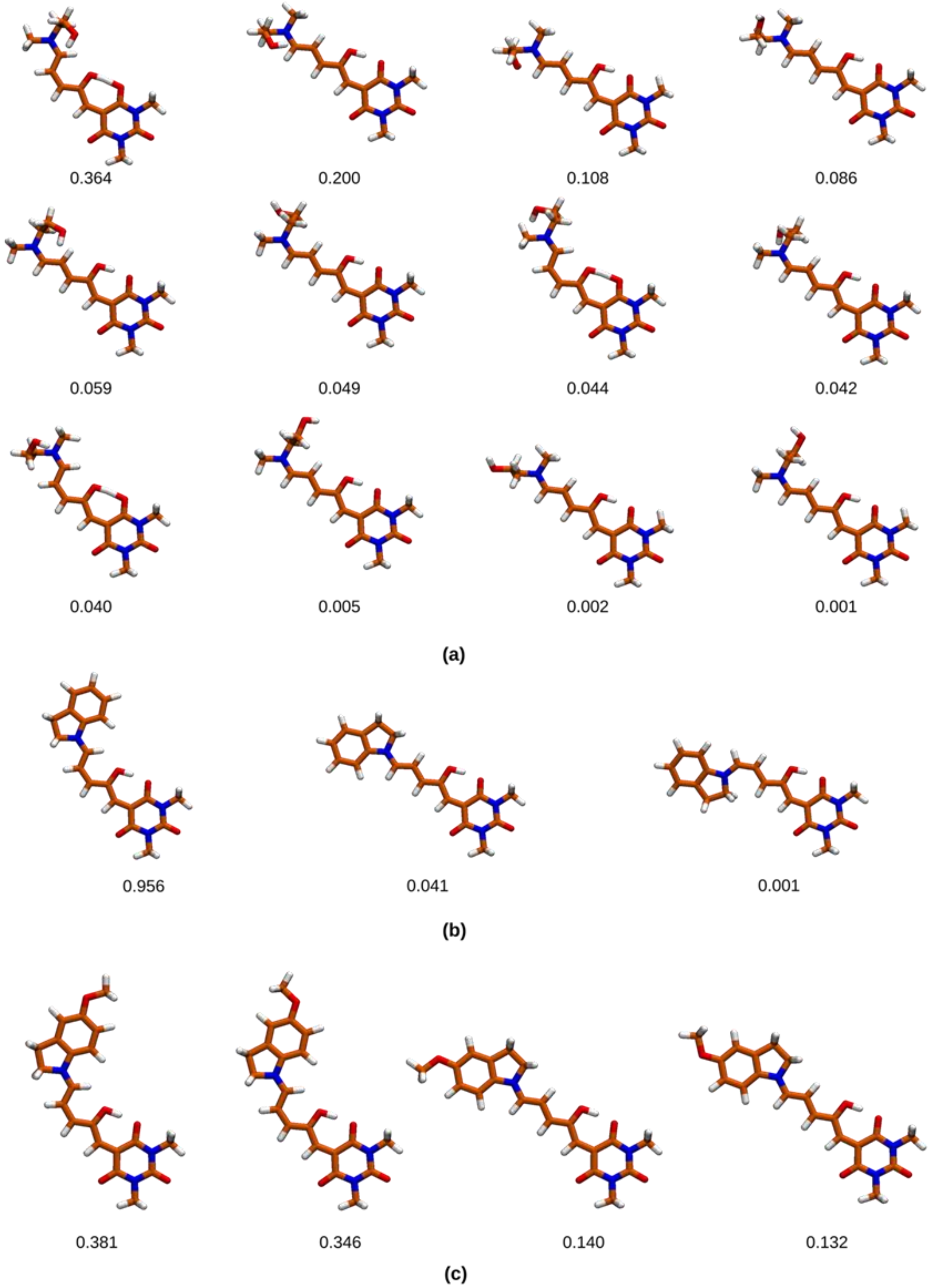

Figure S29. Most relevant conformations for the molecules **1** (a), **2** (b) and **3** (c). The numbers below the structures correspond to the Boltzmann factors evaluated at 298 K.

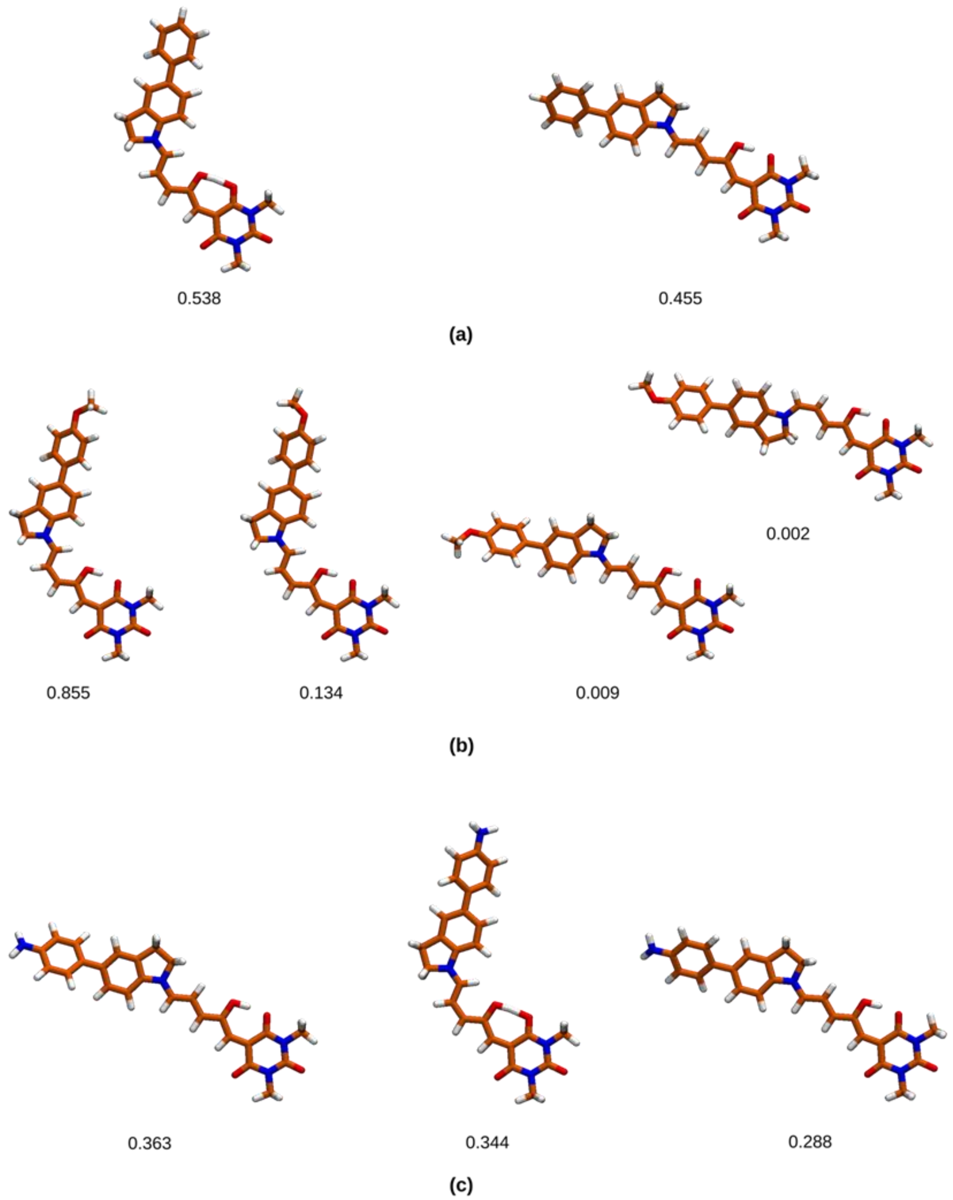

Figure 30. Most relevant conformations for the molecules **4** (a), **6** (b) and **5** (c). The numbers below the structures correspond to the Boltzmann factors evaluated at 298 K.

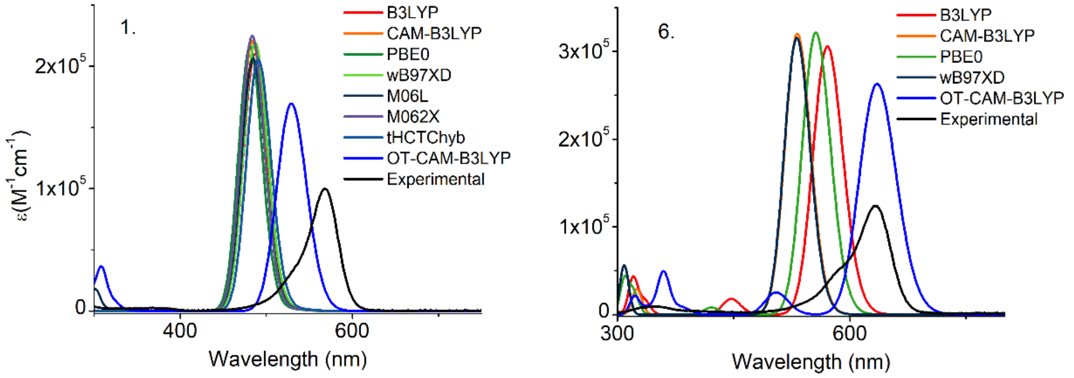

Figure S31. Electronic absorption spectra for molecular systems **1** and **6**, simulated using different density functional models and the def2-SVP basis. As can be seen, the experimental spectra are best predicted by the OT-CAM-B3LYP functional.

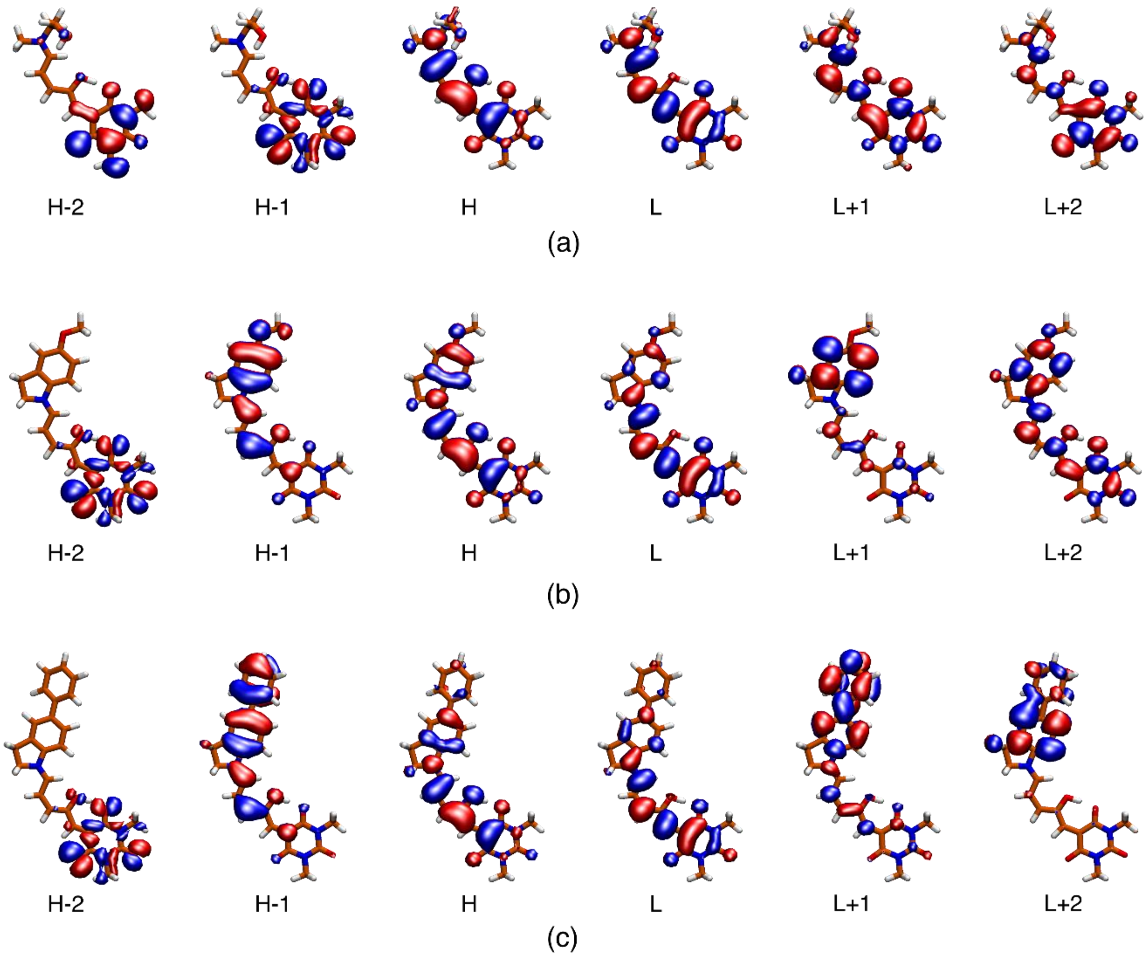

Figure S32. Isosurfaces (0.04) of the most important molecular orbitals involved in the description of the excited singlet electronic states for systems **1**(a), **3**(b) and **4**(c).

Table S8: Vertical transition energies (eV) and oscillator strengths for the low-energy singlet excited states of the most stable geometries for molecular systems **1** to **6**. The results were obtained from OT-CAM-B3LYP/def2-SVP calculations. Figures S28 and S29 show the relevant conformations as well as the corresponding weights for the Boltzmann averaging.

Systems		Conformation											
Molecule	Nature	1	2	3	4	5	6	7	8	9	10	11	12
**1**	$1^1\pi\pi^*$	2.37 (1.07)	2.32 (1.17)	2.34 (1.17)	2.33 (1.17)	2.32 (1.15)	2.33 (1.16)	2.39 (1.08)	2.34 (1.15)	2.40 (1.10)	2.33 (1.15)	2.33 (1.18)	2.34 (1.16)
	$1^1n\pi^*$	2.95 (0.00)	2.92 (0.00)	2.96 (0.00)	2.95 (0.00)	2.93 (0.00)	2.95 (0.00)	2.97 (0.00)	2.94 (0.00)	2.98 (0.00)	2.94 (0.00)	2.93 (0.00)	2.94 (0.00)
	$2^1\pi\pi^*$	3.56 (0.00)	3.54 (0.00)	3.55 (0.00)	3.57 (0.00)	3.55 (0.00)	3.57 (0.00)	3.58 (0.00)	3.56 (0.00)	3.59 (0.00)	3.56 (0.00)	3.52 (0.00)	3.53 (0.00)
	$2^1n\pi^*$	3.59 (0.00)	3.58 (0.00)	3.63 (0.00)	3.60 (0.00)	3.58 (0.00)	3.61 (0.00)	3.62 (0.00)	3.60 (0.00)	3.63 (0.00)	3.60 (0.00)	3.61 (0.00)	3.61 (0.00)
	$3^1\pi\pi^*$	3.76 (0.03)	3.84 (0.13)	3.97 (0.09)	3.94 (0.08)	3.67 (0.04)	3.85 (0.12)	3.87 (0.03)	3.83 (0.10)	3.87 (0.03)	3.82 (0.10)	3.87 (0.15)	3.84 (0.11)
**2**	$1^1\pi\pi^*$	2.25 (1.31)	2.22 (1.56)	2.19 (1.40)									
	$1^1n\pi^*$	2.91 (0.00)	2.89 (0.00)	2.87 (0.00)									
	$2^1\pi\pi^*$	3.38 (0.01)	3.37 (0.02)	3.34 (0.01)									
	$3^1\pi\pi^*$	3.48 (0.04)	3.46 (0.01)	3.45 (0.00)									
	$4^1\pi\pi^*$	3.57 (0.00)	3.56 (0.00)	3.54 (0.00)									
**3**	$1^1\pi\pi^*$	2.11 (1.29)	2.11 (1.34)	2.08 (1.61)	2.08 (1.64)								
	$1^1n\pi^*$	2.85 (0.00)	2.85 (0.00)	2.81 (0.00)	2.82 (0.00)								
	$2^1\pi\pi^*$	3.00 (0.03)	3.03 (0.03)	3.00 (0.01)	3.02 (0.02)								
	$3^1\pi\pi^*$	3.24 (0.07)	3.19 (0.05)	3.21 (0.02)	3.16 (0.01)								
	$4^1\pi\pi^*$	3.45 (0.00)	3.45 (0.00)	3.42 (0.00)	3.43 (0.00)								
**4**	$1^1\pi\pi^*$	2.08 (1.46)	2.06 (1.85)										
	$1^1n\pi^*$	2.81 (0.00)	2.77 (0.00)										
	$2^1\pi\pi^*$	2.99 (0.01)	3.00 (0.01)										
	$3^1\pi\pi^*$	3.24 (0.03)	3.22 (0.03)										
	$4^1\pi\pi^*$	3.33 (0.31)	3.31 (0.16)										
5	$1^1\pi\pi^*$	2.14 (1.48)	2.14 (1.50)	2.12 (1.88)	2.09 (1.70)								
	$2^1\pi\pi^*$	2.85 (0.07)	2.86 (0.07)	2.85 (0.00)	2.82 (0.01)								
	$1^1n\pi^*$	2.87 (0.00)	2.87 (0.00)	2.87 (0.01)	2.83 (0.00)								
	$3^1\pi\pi^*$	3.30 (0.02)	3.30 (0.03)	3.28 (0.01)	3.28 (0.00)								
	$2^1n\pi^*$	3.40 (0.15)	3.41 (0.13)	3.41 (0.11)	3.39 (0.09)								
6	$1^1\pi\pi^*$	1.95 (1.80)	1.96 (1.34)	1.95 (1.80)									
	$2^1\pi\pi^*$	2.45 (0.12)	2.46 (0.23)	2.45 (0.13)									
	$1^1n\pi^*$	2.77 (0.00)	2.80 (0.00)	2.76 (0.00)									
	$3^1\pi\pi^*$	3.15 (0.01)	3.17 (0.03)	3.15 (0.01)									
	$4^1\pi\pi^*$	3.26 (0.04)	3.26 (0.06)	3.26 (0.04)									

Table S9: TPA cross sections (σ_2, GM) for the low-energy singlet excited states of the molecular systems **1** to **6**, obtained from OT-CAM-B3LYP/def2-SVP calculations. Figures S28 and S29 show the relevant conformations as well as the corresponding weights for the Boltzmann averaging. The calculations for σ_2 were obtained from quadratic response calculations from the Dalton program

Systems		Conformation											
Molecule	Nature	1	2	3	4	5	6	7	8	9	10	11	12
**1**	$1^1\pi\pi^*$	5.78	6.10	3.05	3.08	6.85	4.72	4.63	5.38	4.87	6.45	6.45	6.02
	$1^1n\pi^*$	0.02	0.01	0.01	0.01	0.01	0.01	0.01	0.01	0.01	0.01	0.01	0.01
	$2^1\pi\pi^*$	4.56	6.97	6.88	6.93	5.11	6.49	4.77	6.01	5.27	5.48	6.49	5.37
	$2^1n\pi^*$	1.39	0.12	0.01	0.04	0.02	0.01	0.19	0.00	0.02	0.03	0.03	0.02
	$3^1\pi\pi^*$	46.80	422.00	4740.00	5110.00	41.90	417.00	216.00	165.00	138.00	149.00	504.00	192.00
**2**	$1^1\pi\pi^*$	24.70	28.20	21.30									
	$1^1n\pi^*$	0.01	0.01	0.02									
	$2^1\pi\pi^*$	431.00	711.00	529.00									
	$3^1\pi\pi^*$	53.20	19.80	27.10									
	$4^1\pi\pi^*$	8.08	8.60	9.80									
**3**	$1^1\pi\pi^*$	51.50	58.40	54.80	60.80								
	$1^1n\pi^*$	0.02	0.01	0.02	0.02								
	$2^1\pi\pi^*$	861.00	934.00	1490.00	1490.00								
	$3^1\pi\pi^*$	17.10	32.90	7.75	19.50								
	$4^1\pi\pi^*$	17.70	18.50	10.80	11.80								
**4**	$1^1\pi\pi^*$	25.60	23.40										
	$1^1n\pi^*$	0.01	0.01										
	$2^1\pi\pi^*$	3080.00	4960.00										
	$3^1\pi\pi^*$	263.00	459.00										
	$4^1\pi\pi^*$	319.00	1000.00										
5	$1^1\pi\pi^*$	133.00	135.00	151.00	156.00								
	$2^1\pi\pi^*$	3080.00	3180.00	5090.00	4560.00								
	$1^1n\pi^*$	0.02	0.13	0.14	5.61								
	$3^1\pi\pi^*$	54.30	32.20	49.80	75.30								
	$2^1n\pi^*$	2.78	0.86	8.14	308.00								
6	$1^1\pi\pi^*$	654.00	560.00	671.00									
	$2^1\pi\pi^*$	4750.00	2790.00	4720.00									
	$1^1n\pi^*$	0.02	0.01	0.02									
	$3^1\pi\pi^*$	270.00	297.00	258.00									
	$4^1\pi\pi^*$	4260.00	1790.00	4250.00									

NMR Characterization of all the compounds

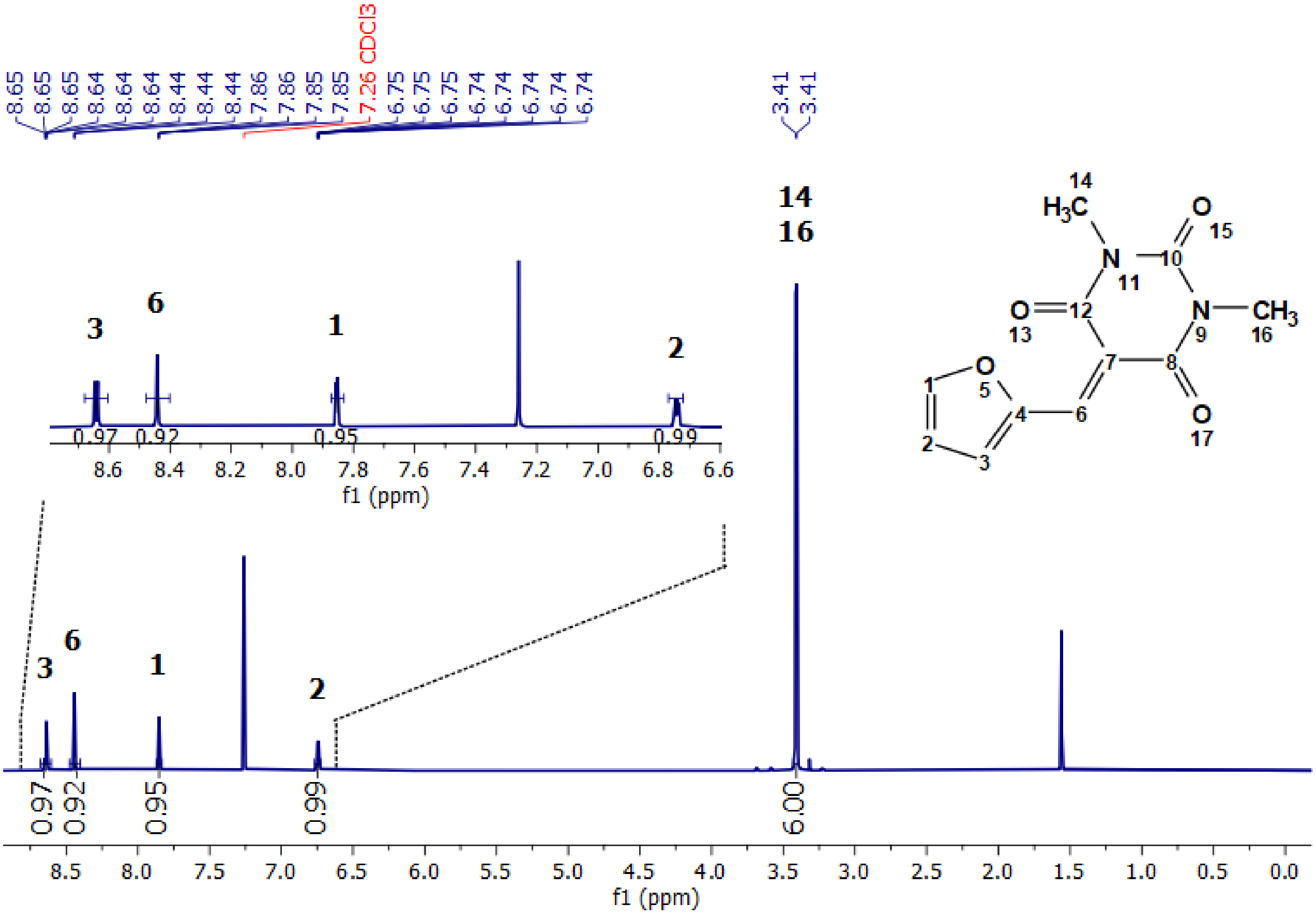

Figure S33. ^{1}H NMR spectrum of activated furane (400 MHz, $CDCl_3$, 298 K).

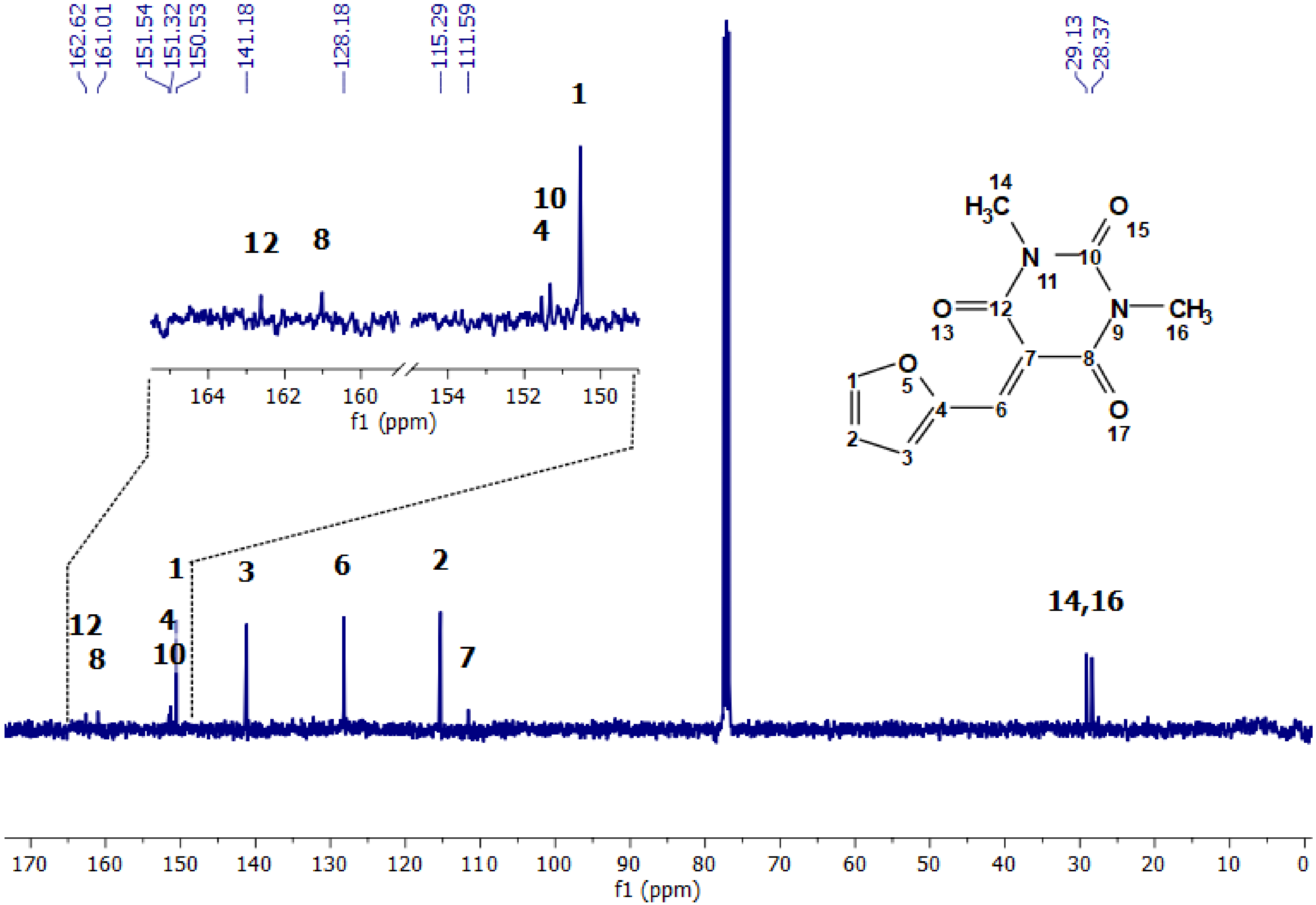

Figure S34. ^{13}C NMR spectrum of activated furane (101 MHz, $CDCl_3$, 298 K).

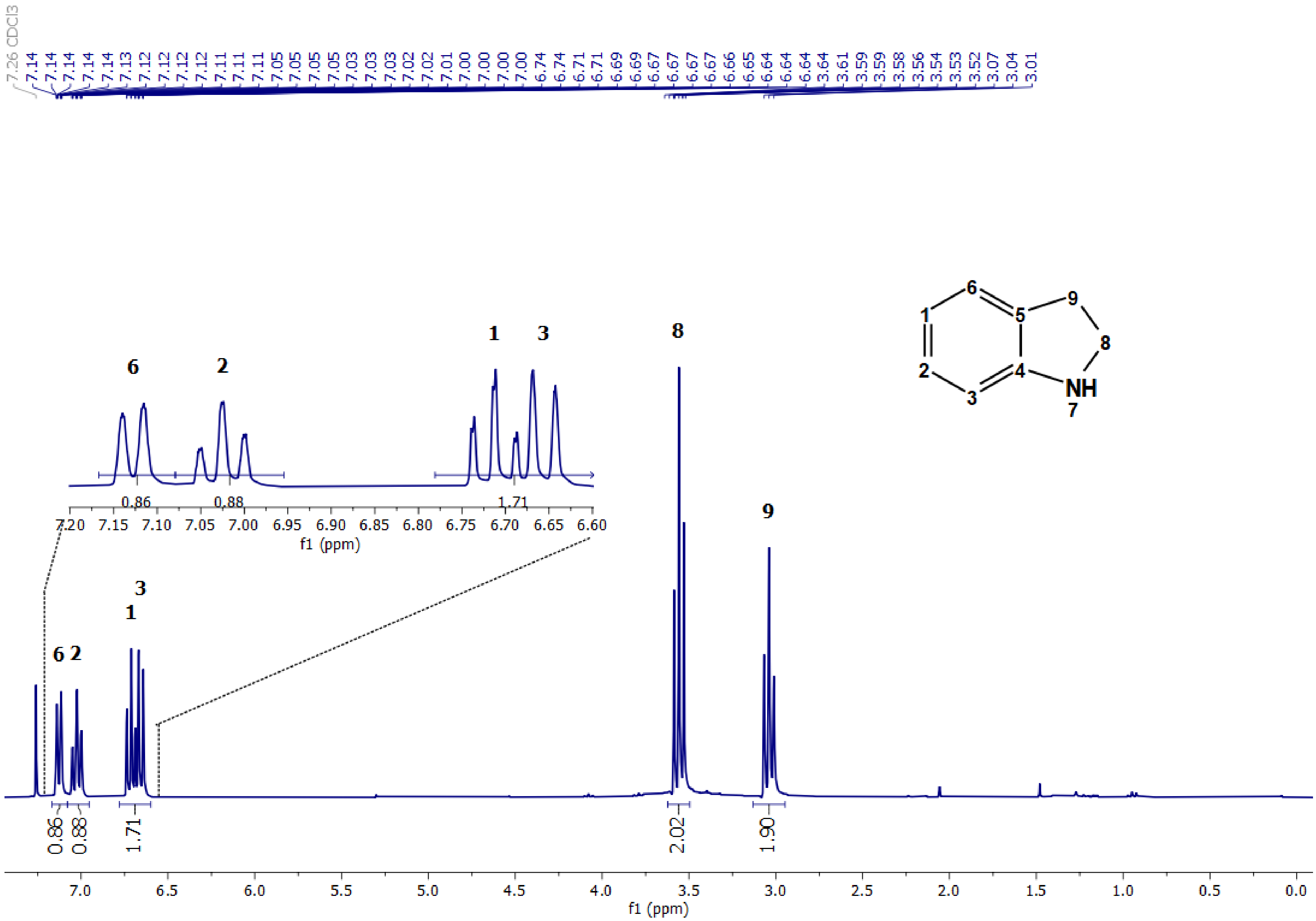

Figure S35. ^{1}H NMR spectrum of indoline (301 MHz, $CDCl_3$, 298 K).

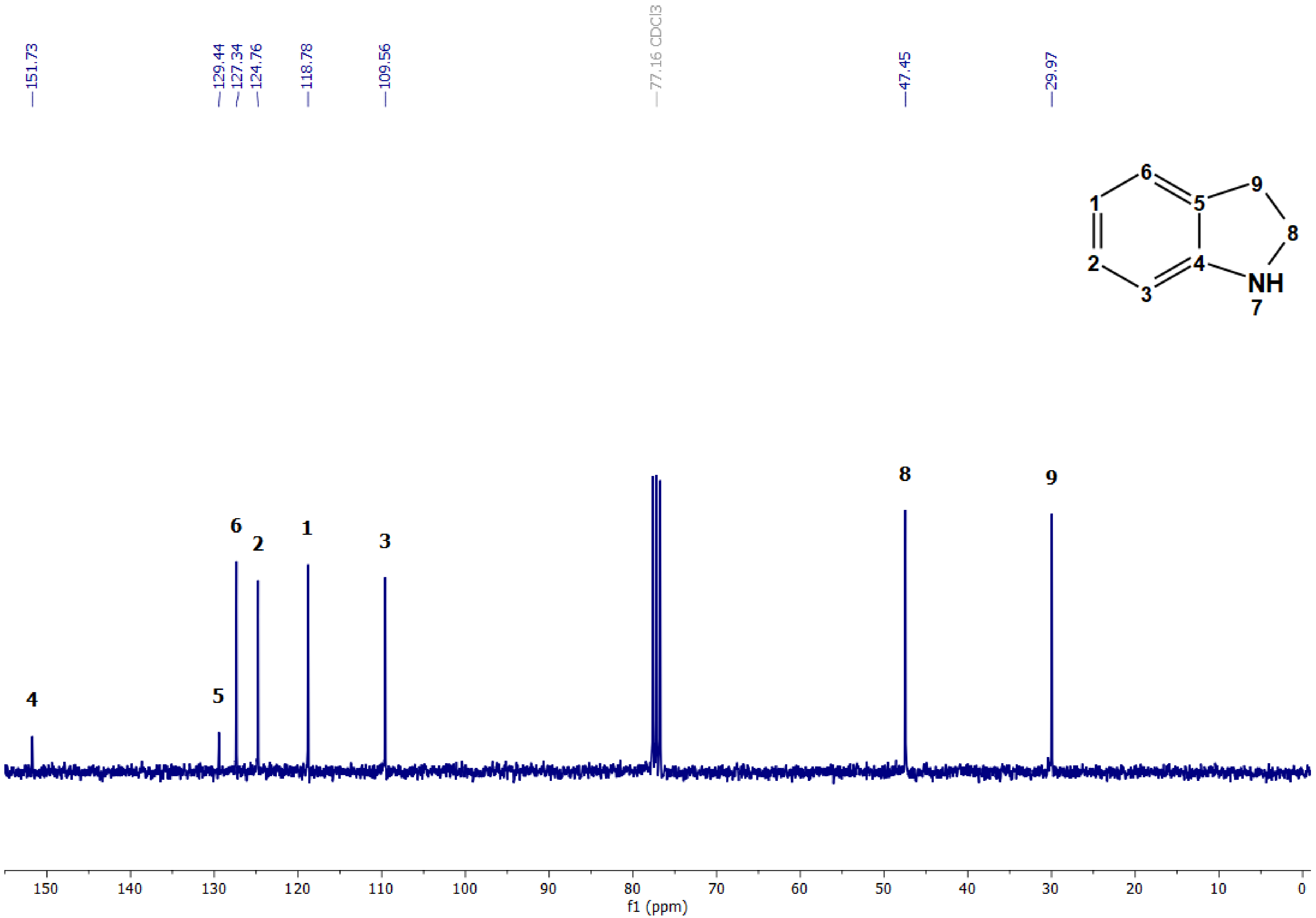

Figure S36. ^{13}C NMR spectrum of indoline (76 MHz, $CDCl_3$, 298 K).

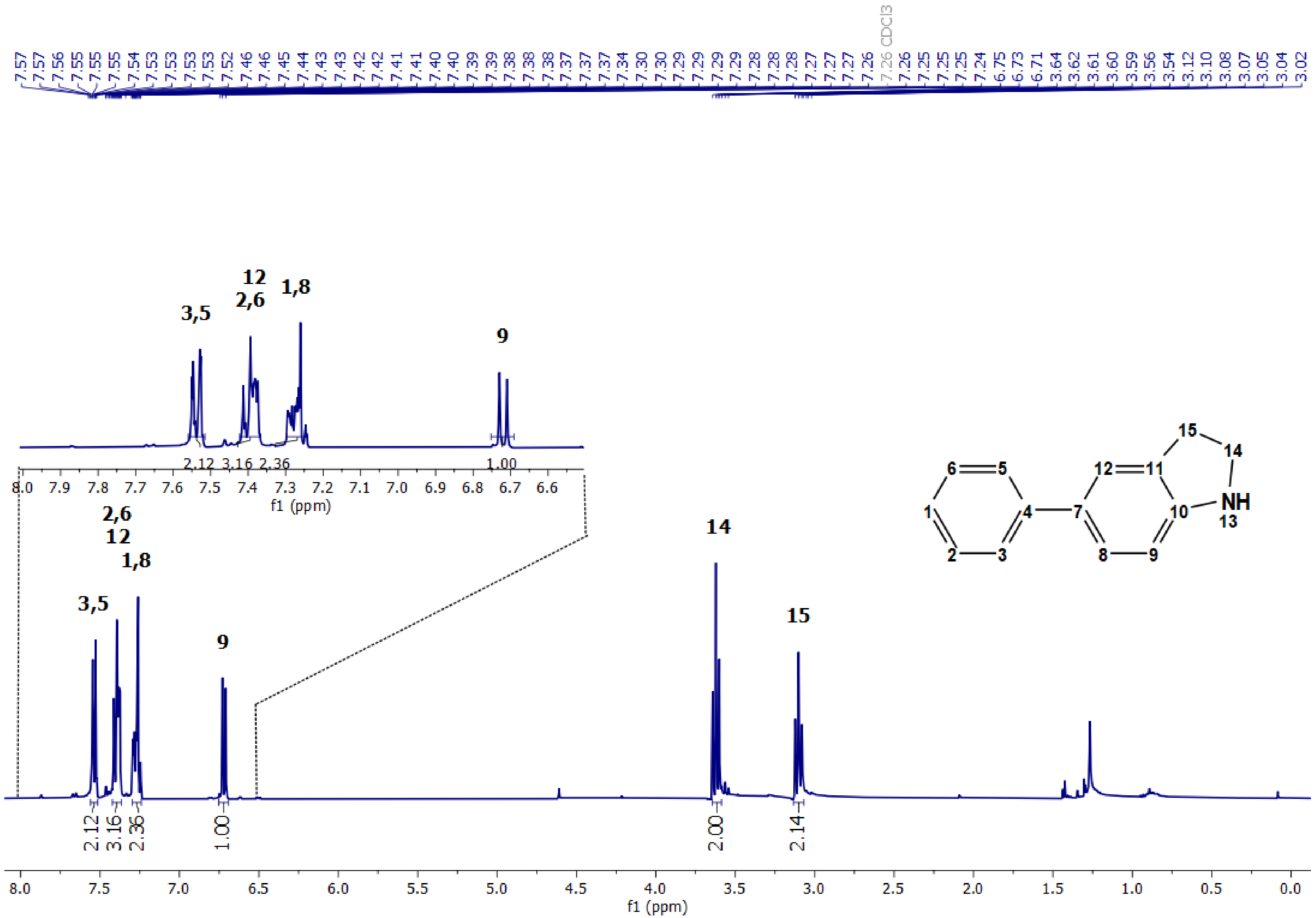

Figure S37. ^{1}H NMR spectrum of 5-phenylindoline (400 MHz, $CDCl_3$, 298 K).

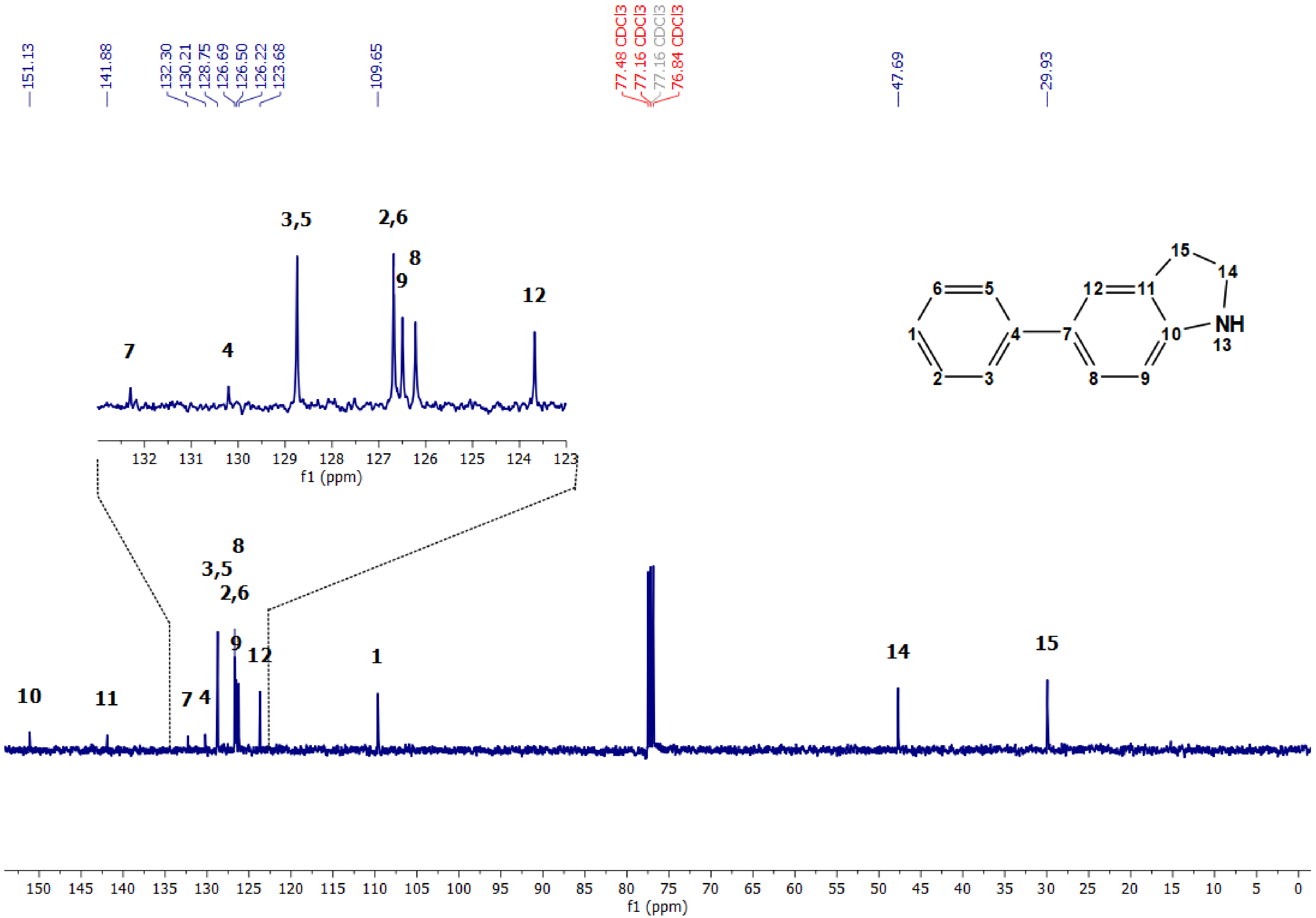

Figure S38. ^{13}C NMR spectrum of 5-phenylindoline (101 MHz, $CDCl_3$, 298 K).

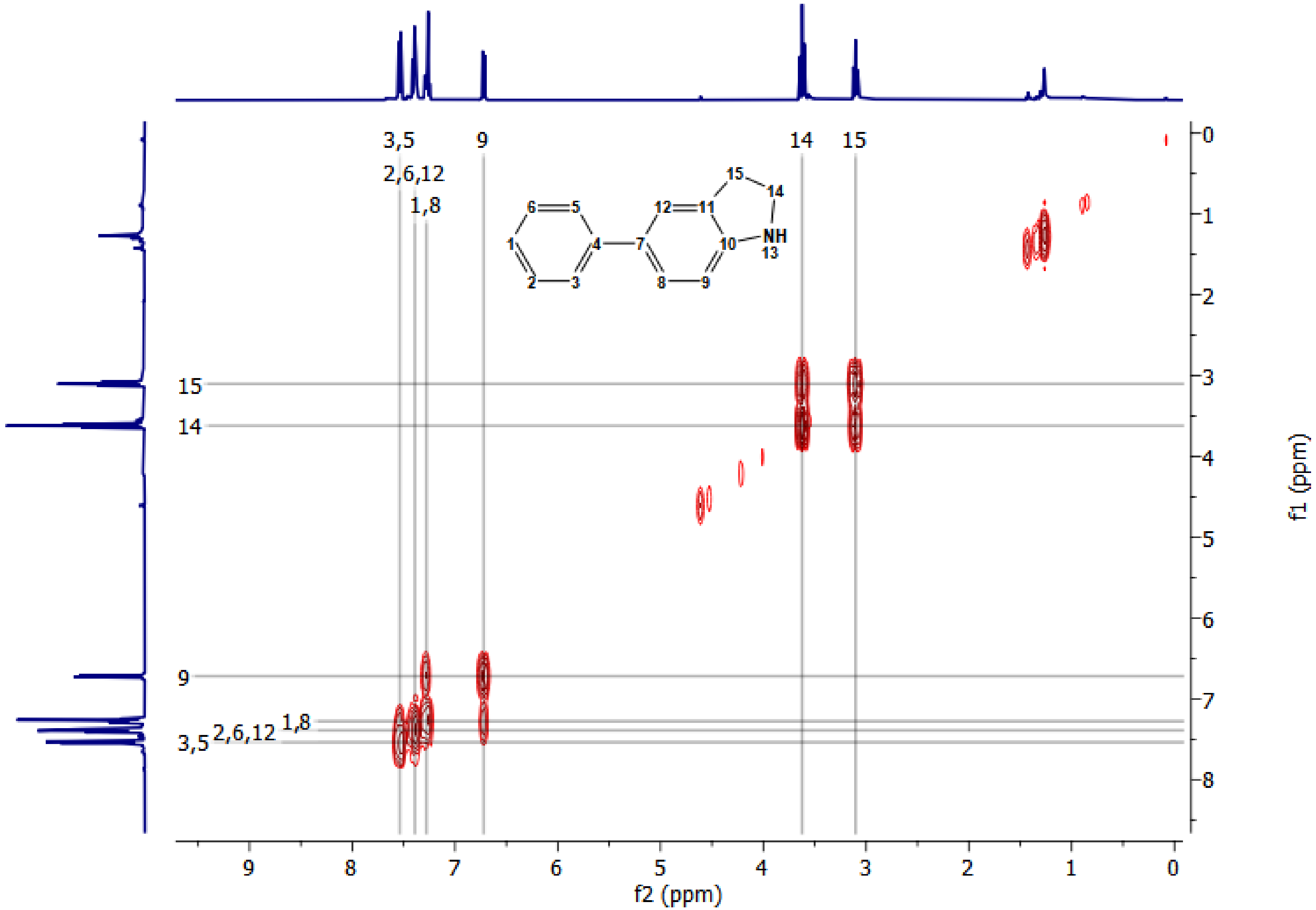

Figure S39. COSY spectrum of 5-phenylindoline (400 MHz, $CDCl_3$, 298 K).

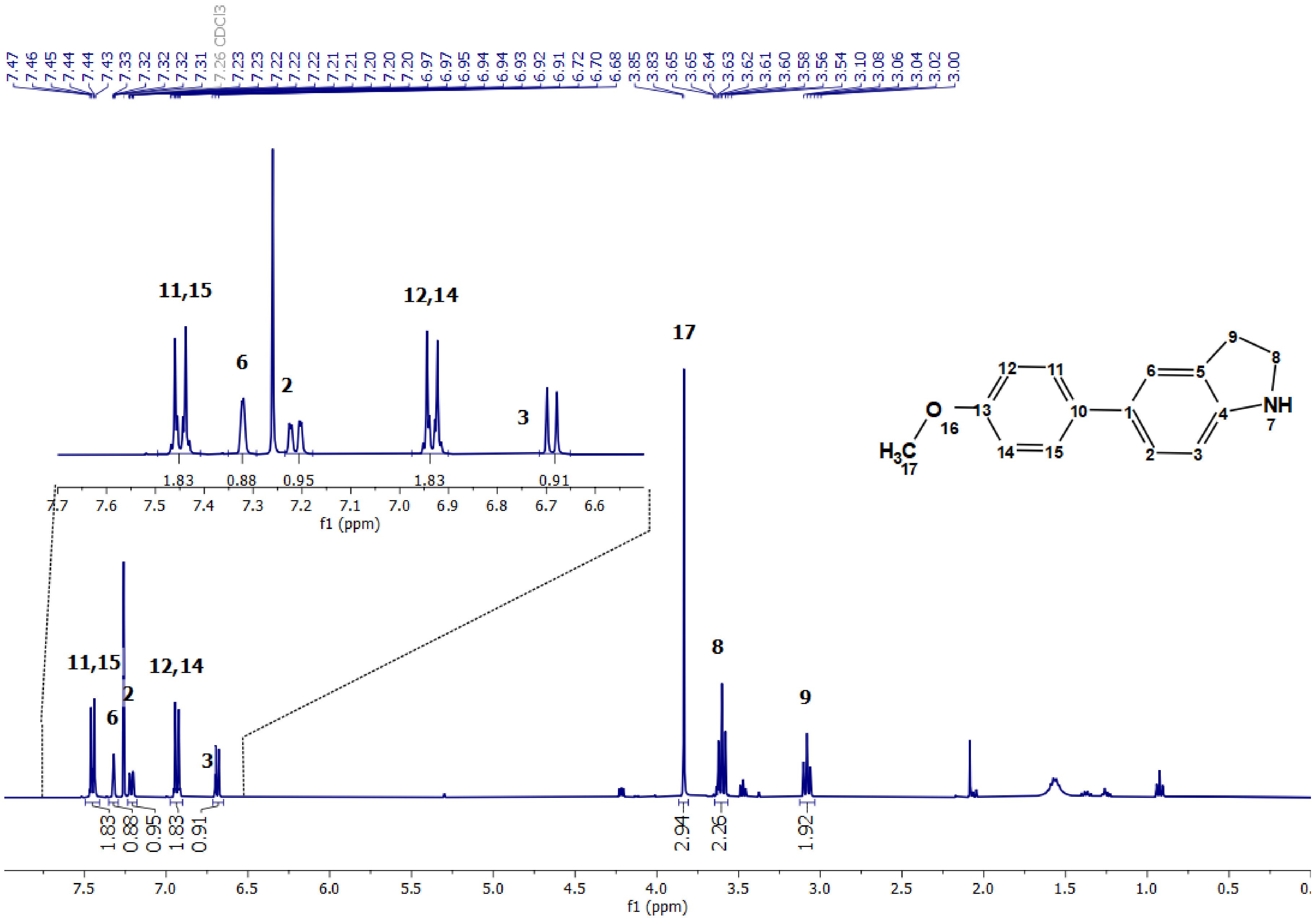

Figure S40. ^{1}H NMR spectrum of 5-(4-methoxyphenyl)indoline (400 MHz, $CDCl_3$, 298 K).

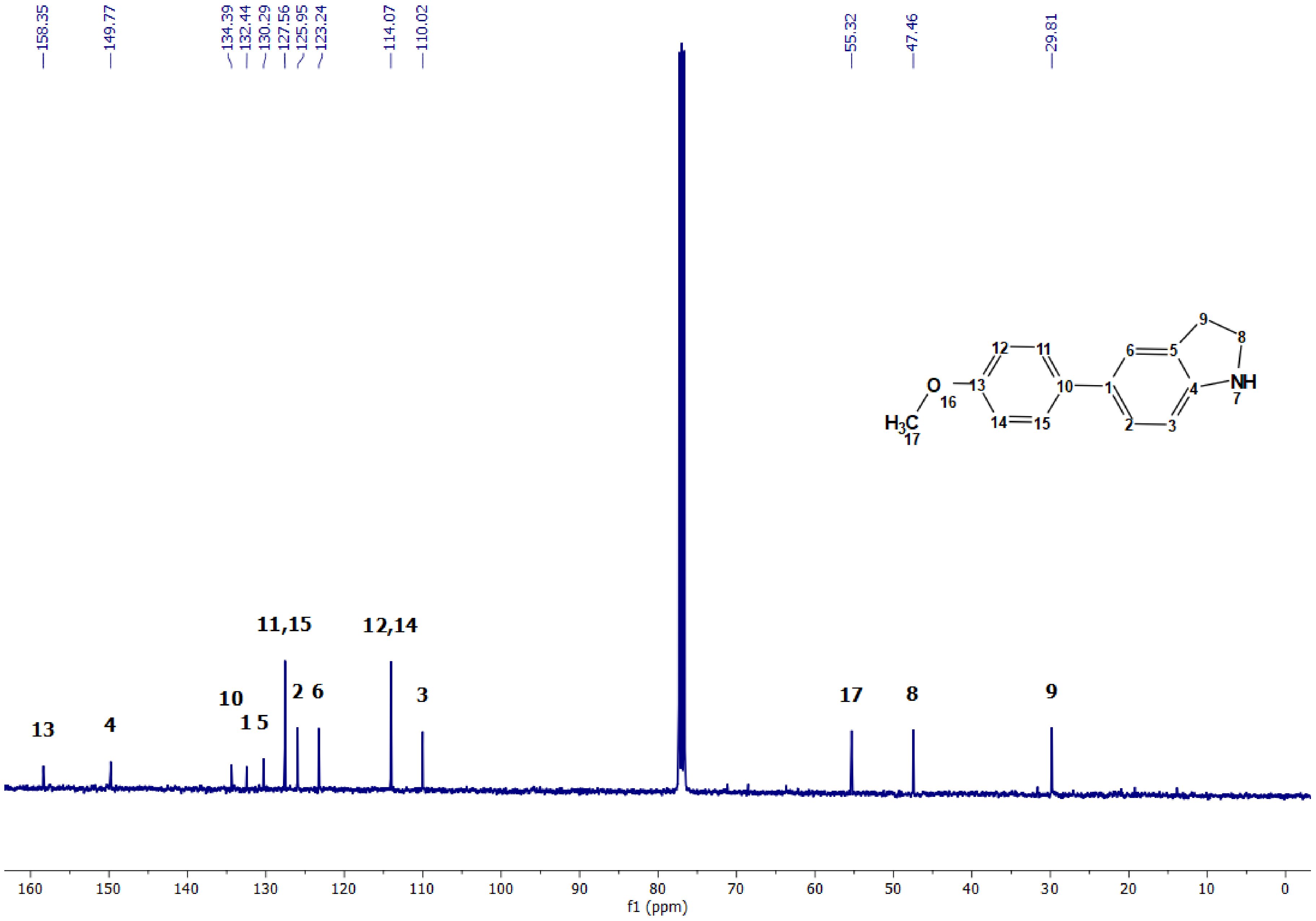

Figure S41. ^{13}C NMR spectrum of 5-(4-methoxyphenyl)indoline (101 MHz, $CDCl_3$, 298 K).

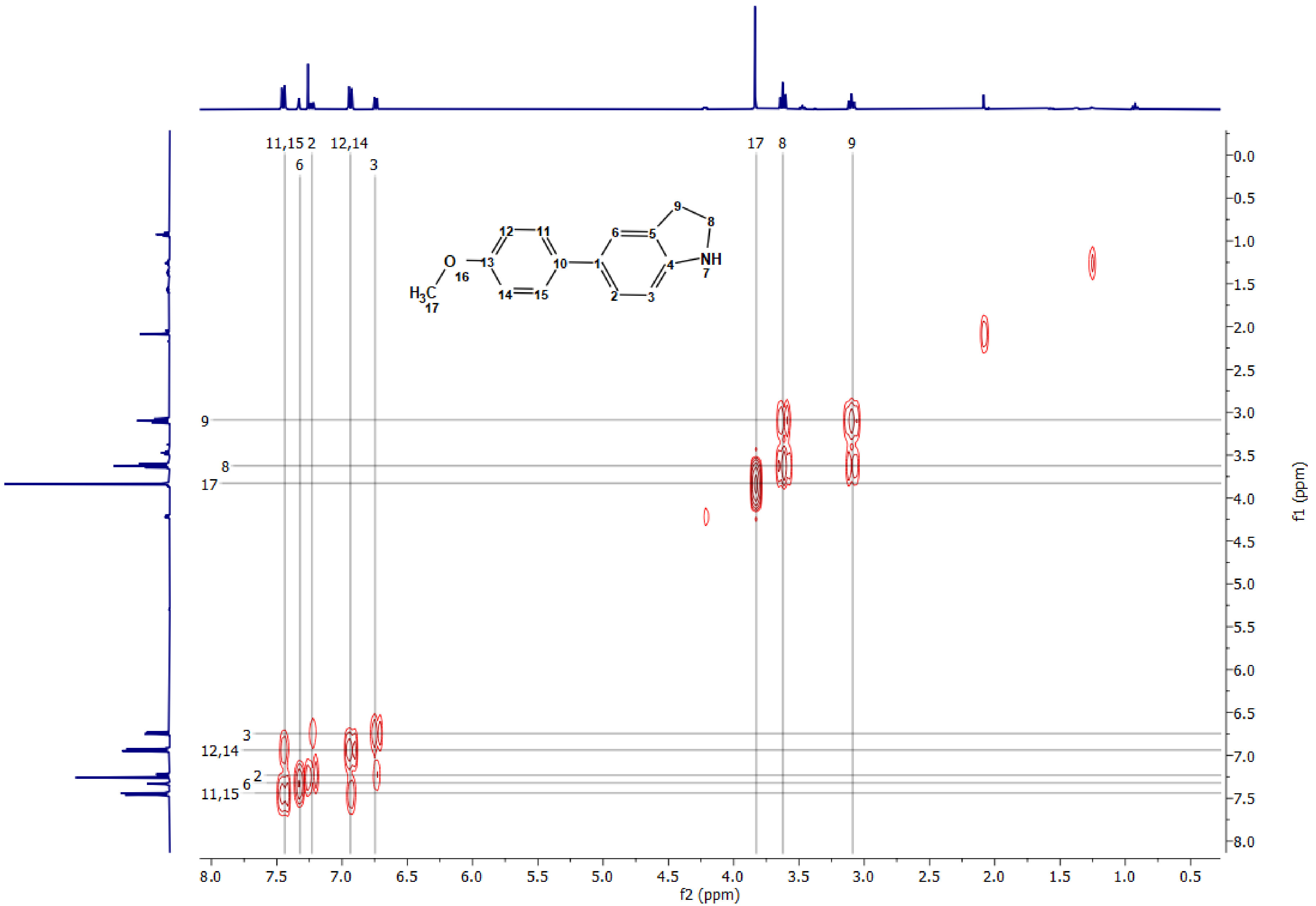

Figure S42. COSY NMR spectrum of 5-(4-methoxyphenyl)indoline (400 MHz, $CDCl_3$, 298 K).

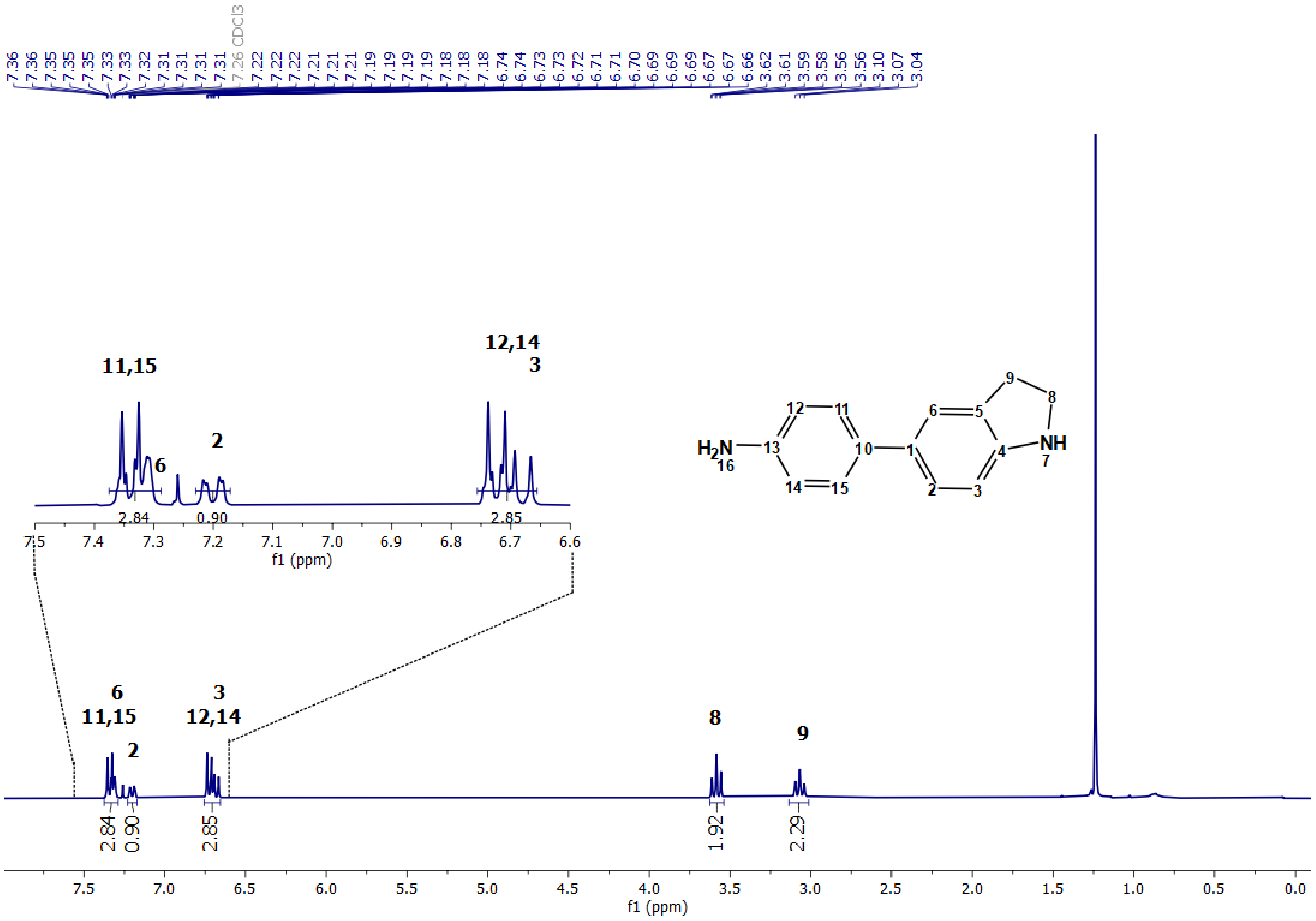

Figure S43. ^{1}H NMR spectrum of 4-(indolin-5-yl)aniline (300 MHz, $CDCl_3$, 295.5 K).

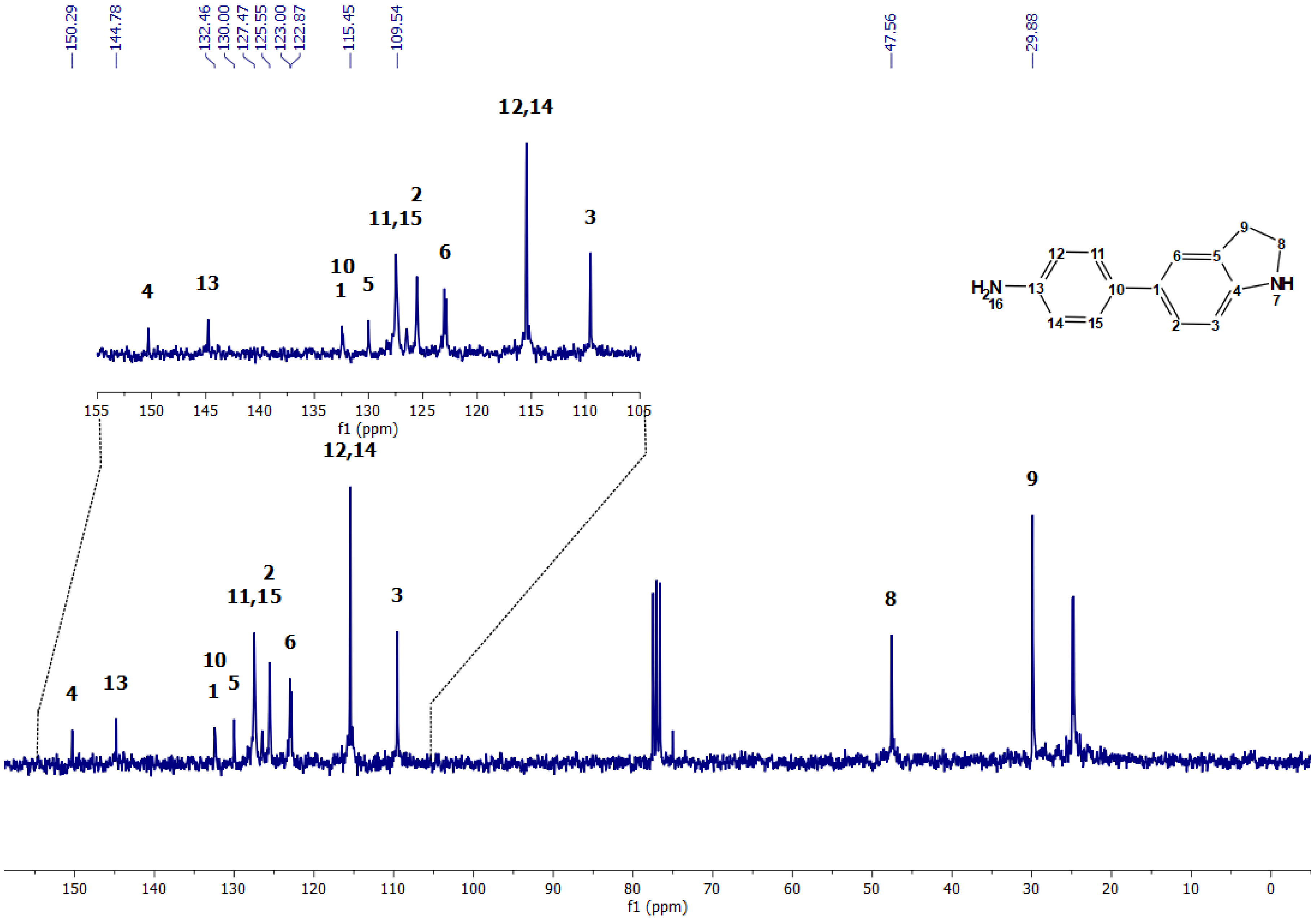

Figure S44. ^{13}C NMR spectrum of 4-(indolin-5-yl)aniline (75 MHz, $CDCl_3$, 295.5 K).

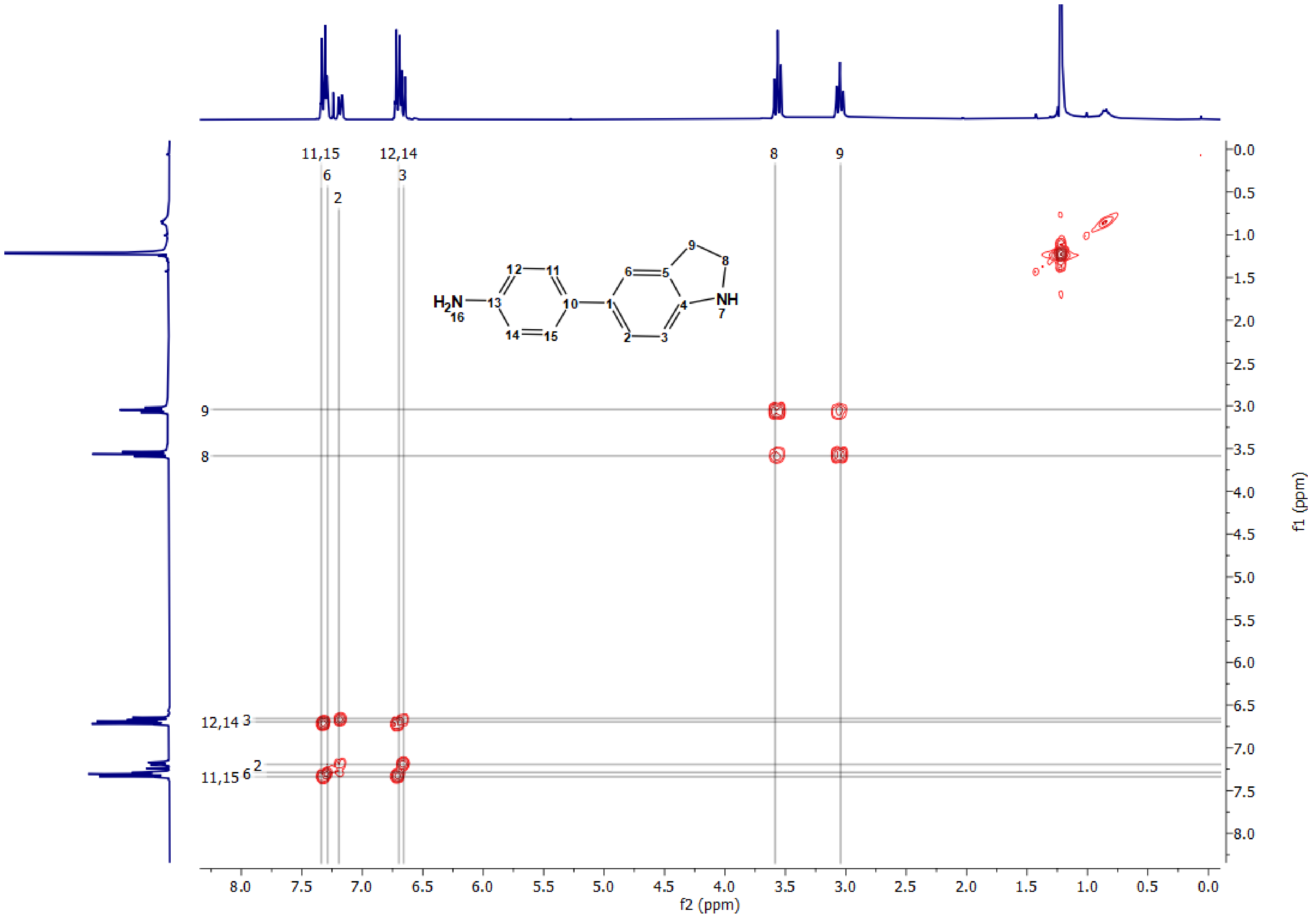

Figure S45. COSY NMR spectrum of 4-(indolin-5-yl)aniline (300 MHz, $CDCl_3$, 295.5 K).

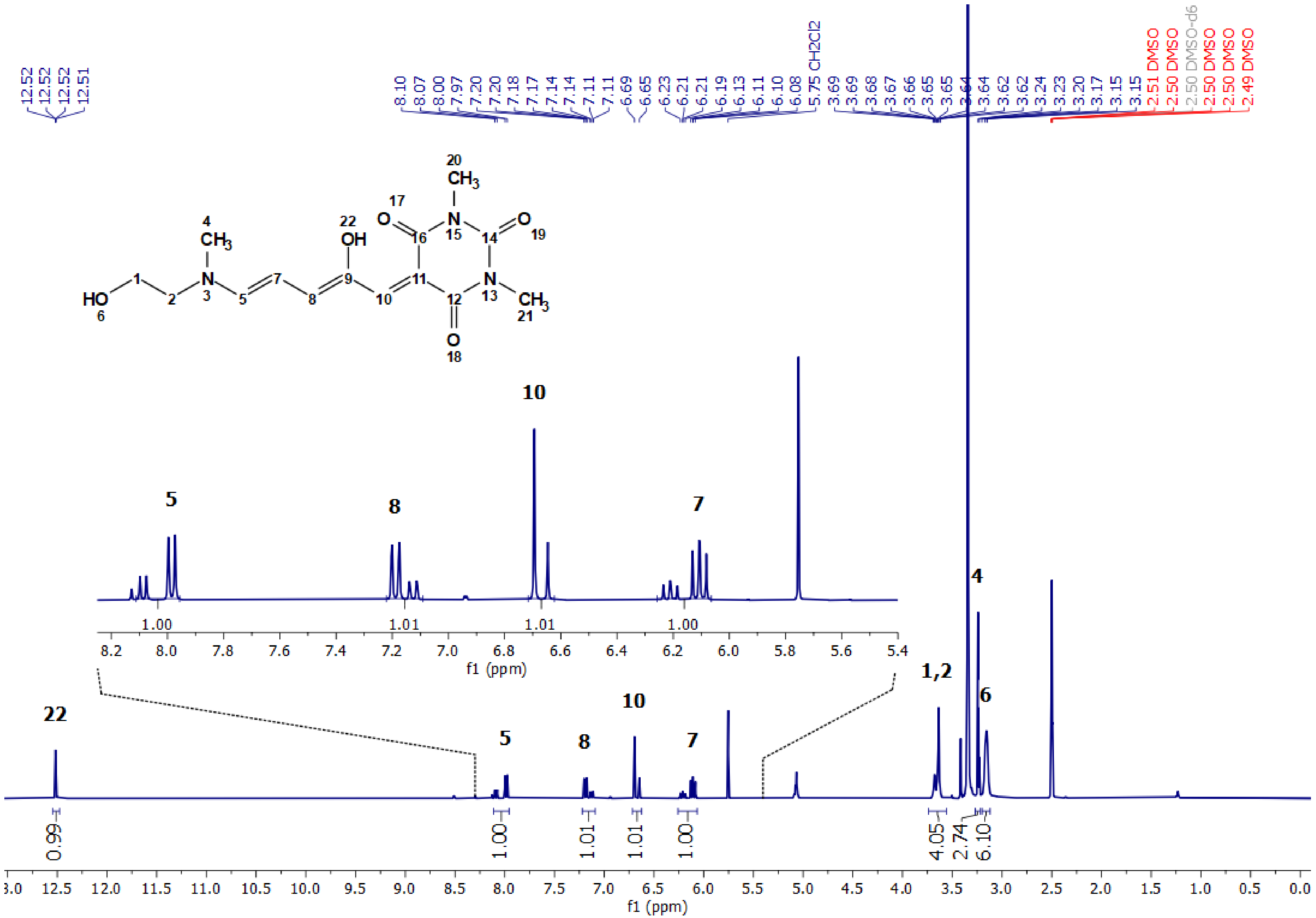

Figure S46. ^{1}H NMR spectrum of DASA **1** (500 MHz, DMSO-d_6, 300 K).

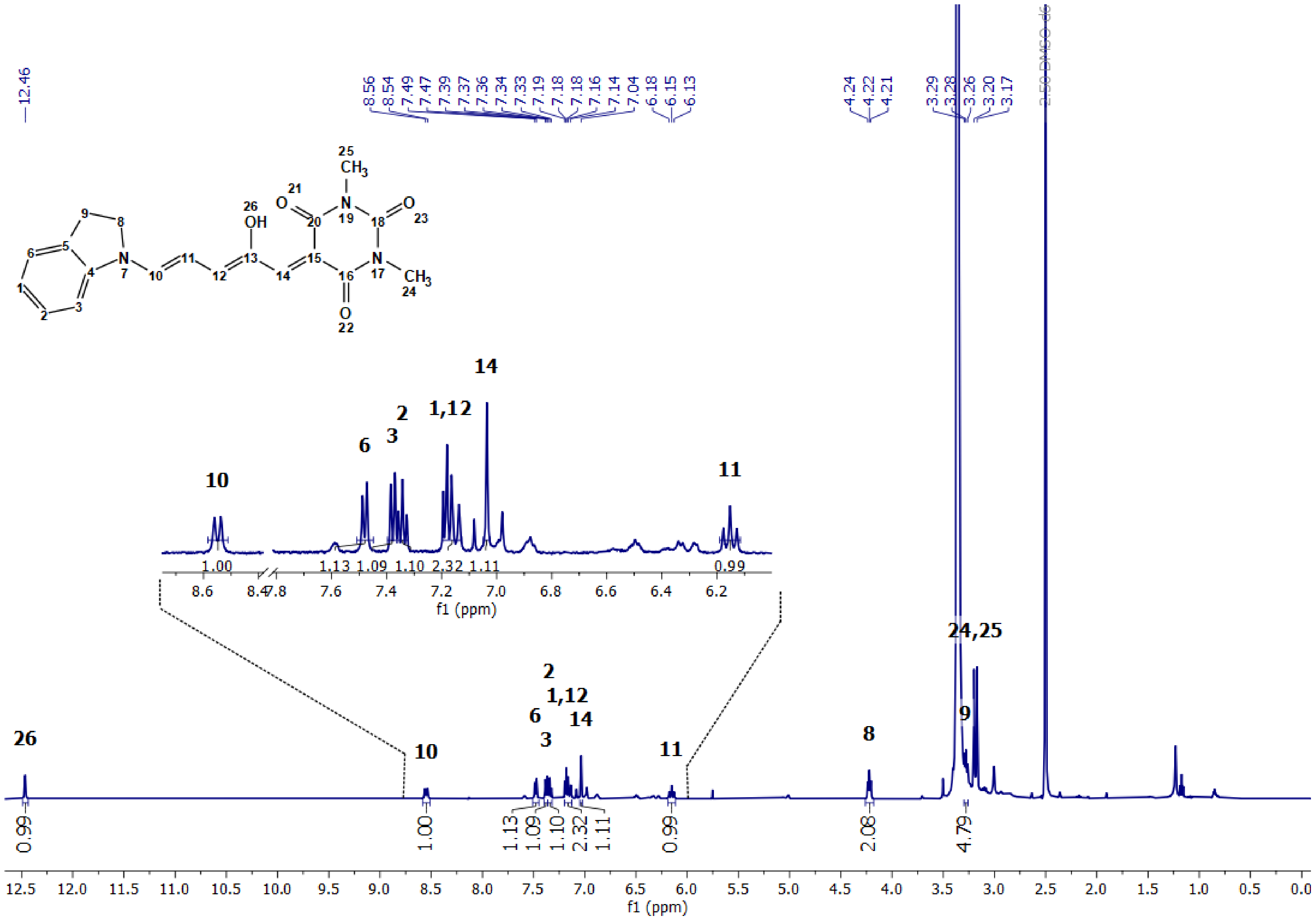

Figure S47. ^{1}H NMR spectrum of DASA **2** (500 MHz, DMSO-d_6, 300 K).

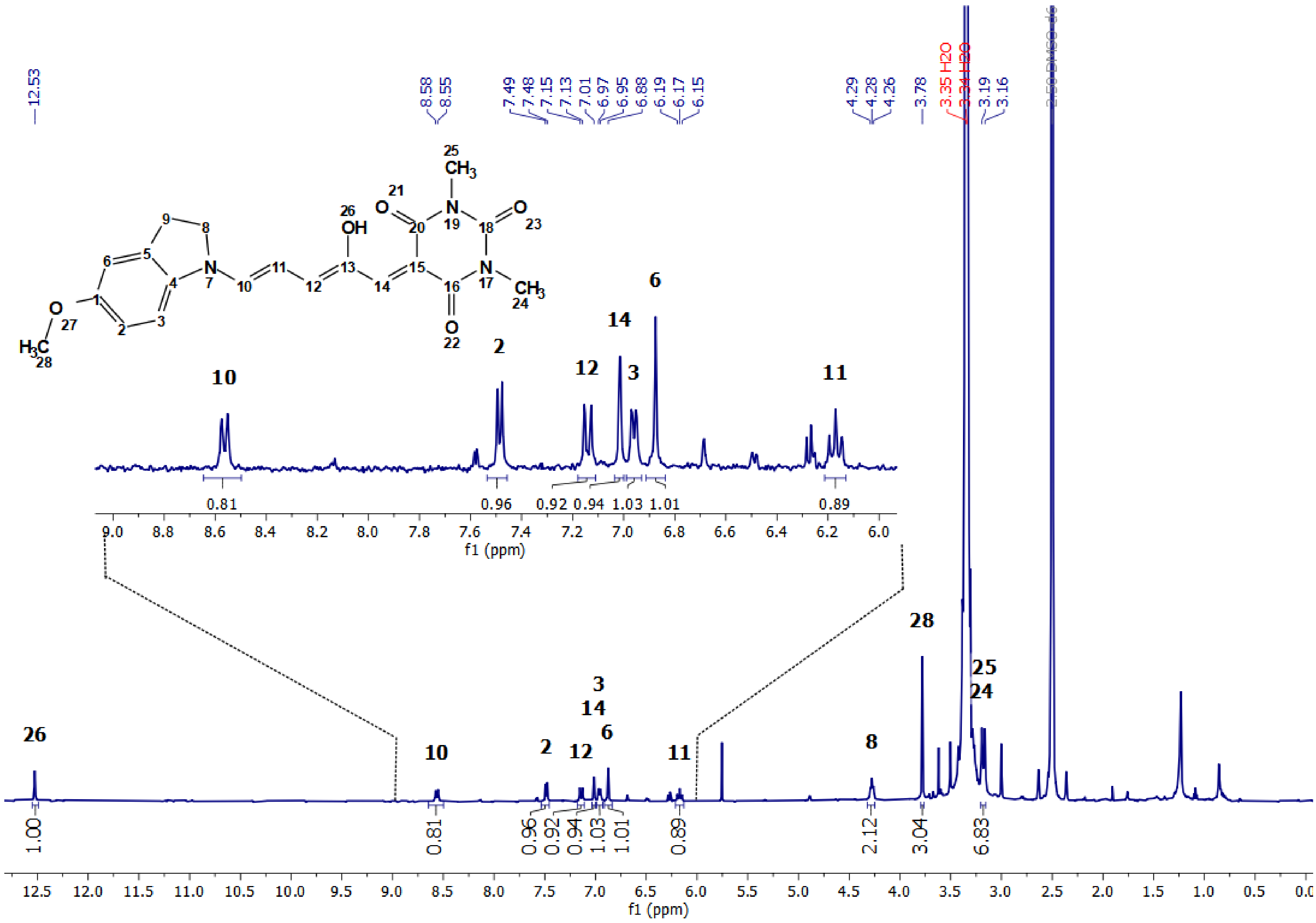

Figure S48. ^{1}H NMR spectrum of DASA **3** (500 MHz, DMSO-d6, 300 K).

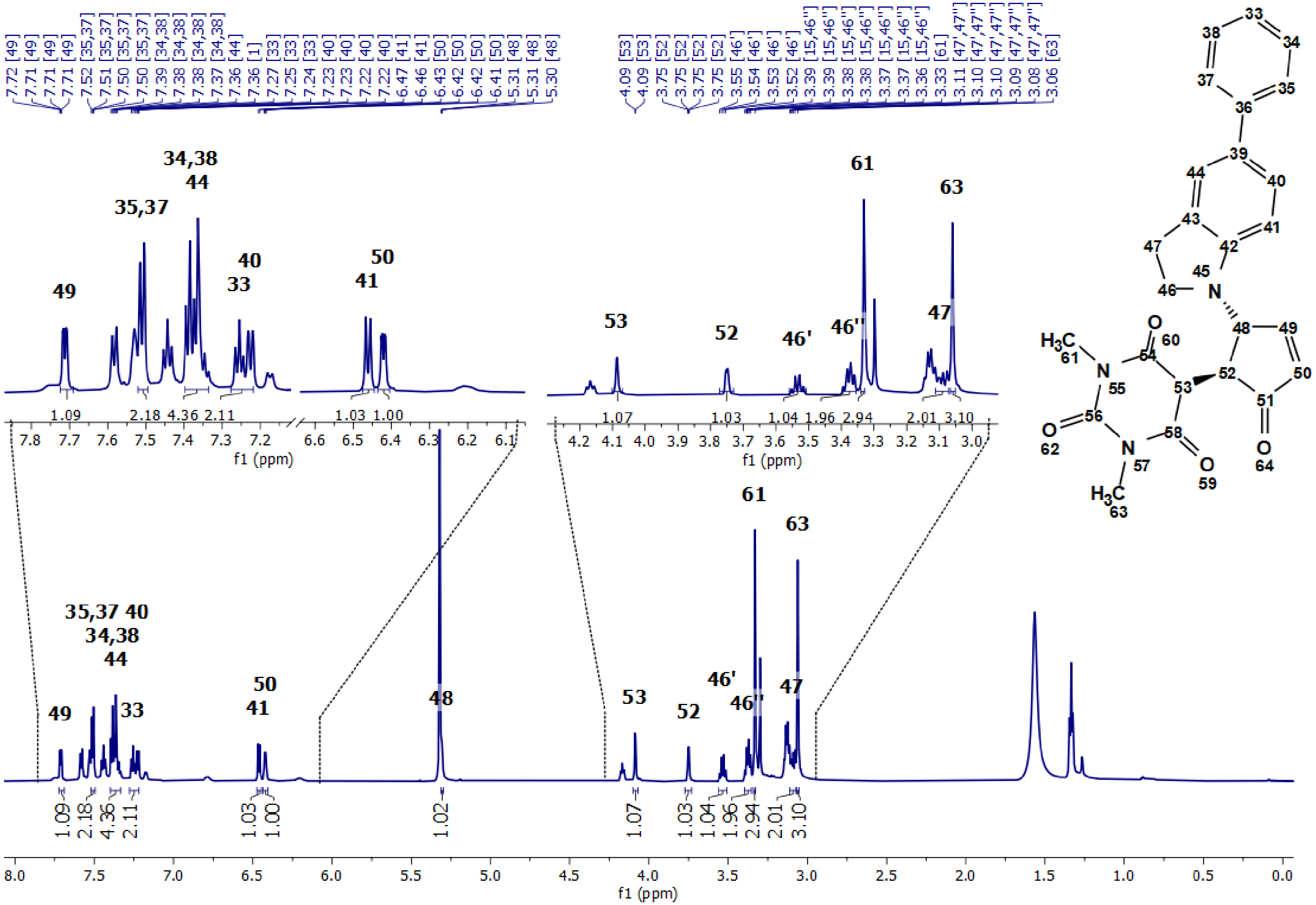

Figure S49. ^{1}H NMR spectrum of DASA **4** in closed form (700 MHz, CD_2Cl_2, 300 K). For the acquisition of ^{1}H and ^{13}C, COSY, HSQC and HMBC NMR data of DASA **4** in the 700 MHz equipment, right before the acquisition of the spectra, the samples were irradiated at the sample handling robot. This increases drastically the population of the cylopentenone isomers which facilitates the assignments. The minor signals correspond to the open isomer.

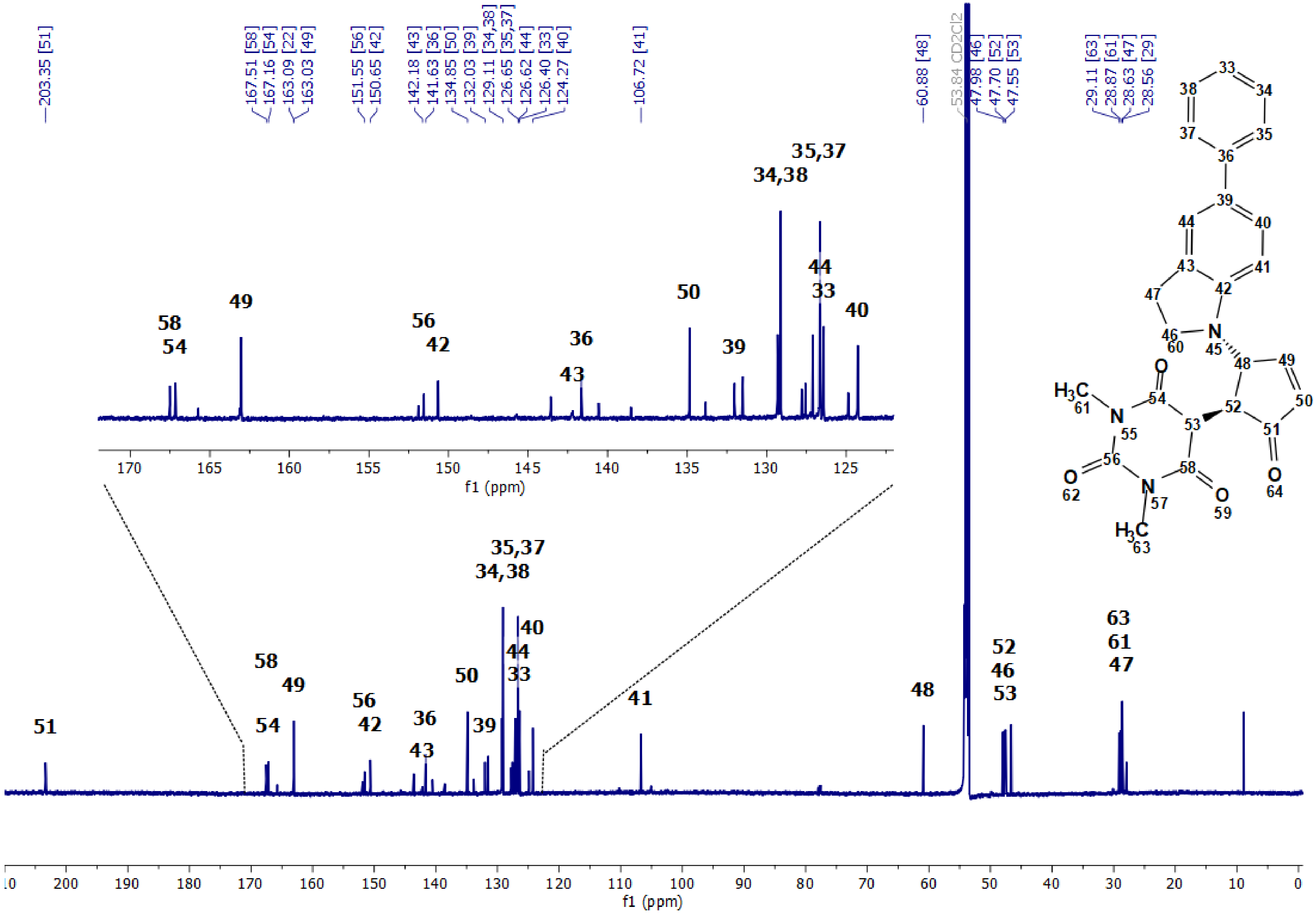

Figure S50. ^{13}C NMR spectrum of DASA **4** in closed form (176 MHz, CD_2Cl_2, 300 K). For the acquisition of ^{1}H, ^{13}C, COSY, HSQC and HMBC NMR data of DASA **4** in the 700 MHz equipment, right before the acquisition of the spectra, the samples were irradiated at the sample handling robot. This increases drastically the population of the cylopentenone isomers which facilitates the assignments. The minor signals correspond to the open isomer.

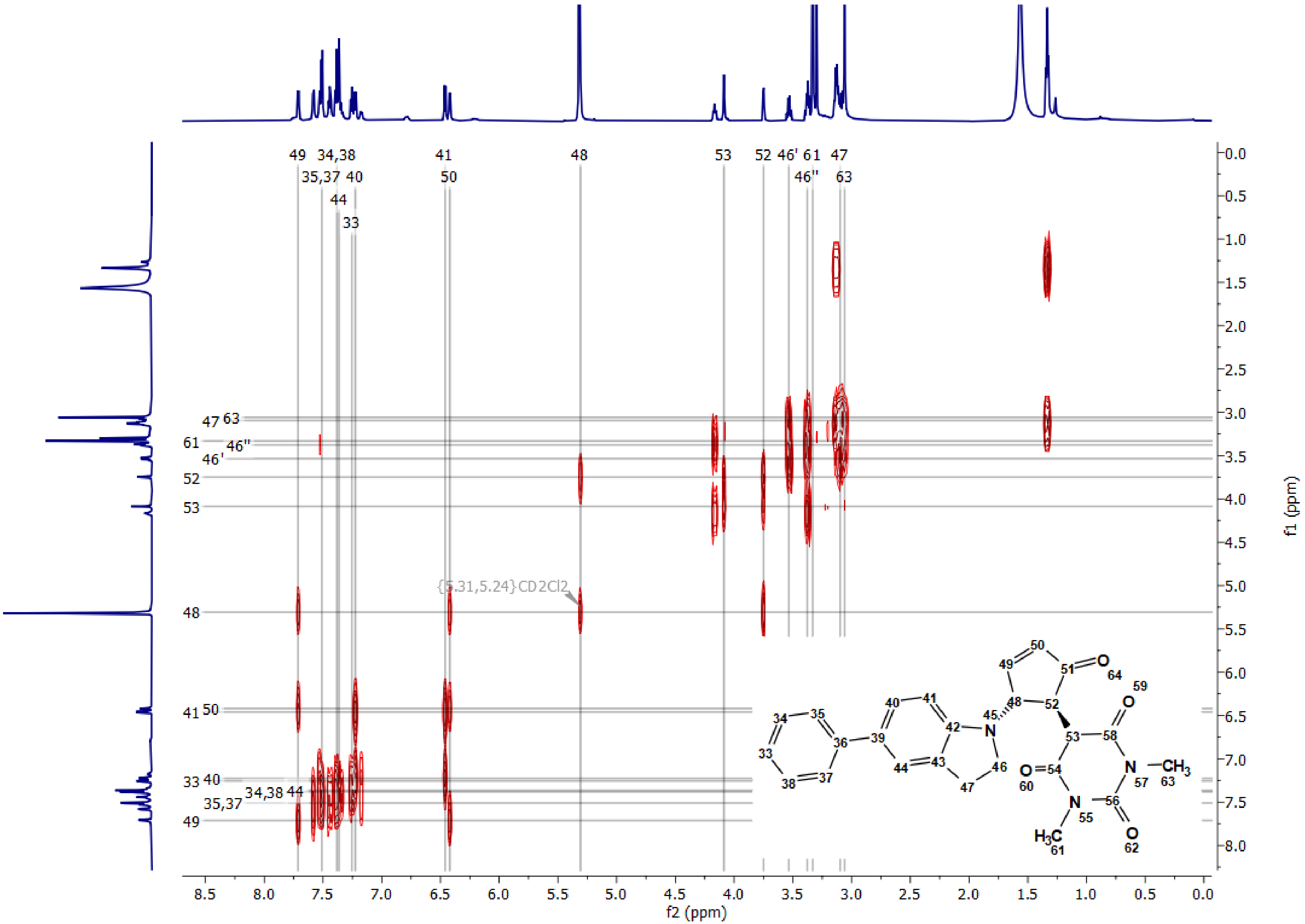

Figure S51. COSY NMR spectrum of DASA **4** in closed form (700 MHz, CD_2Cl_2, 300 K). For the acquisition of ^{1}H, ^{13}C, COSY, HSQC and HMBC NMR data of DASA **4** in the 700 MHz equipment, right before the acquisition of the spectra, the samples were irradiated at the sample handling robot. This increases drastically the population of the cylopentenone isomers which facilitates the assignments. The minor signals correspond to the open isomer.

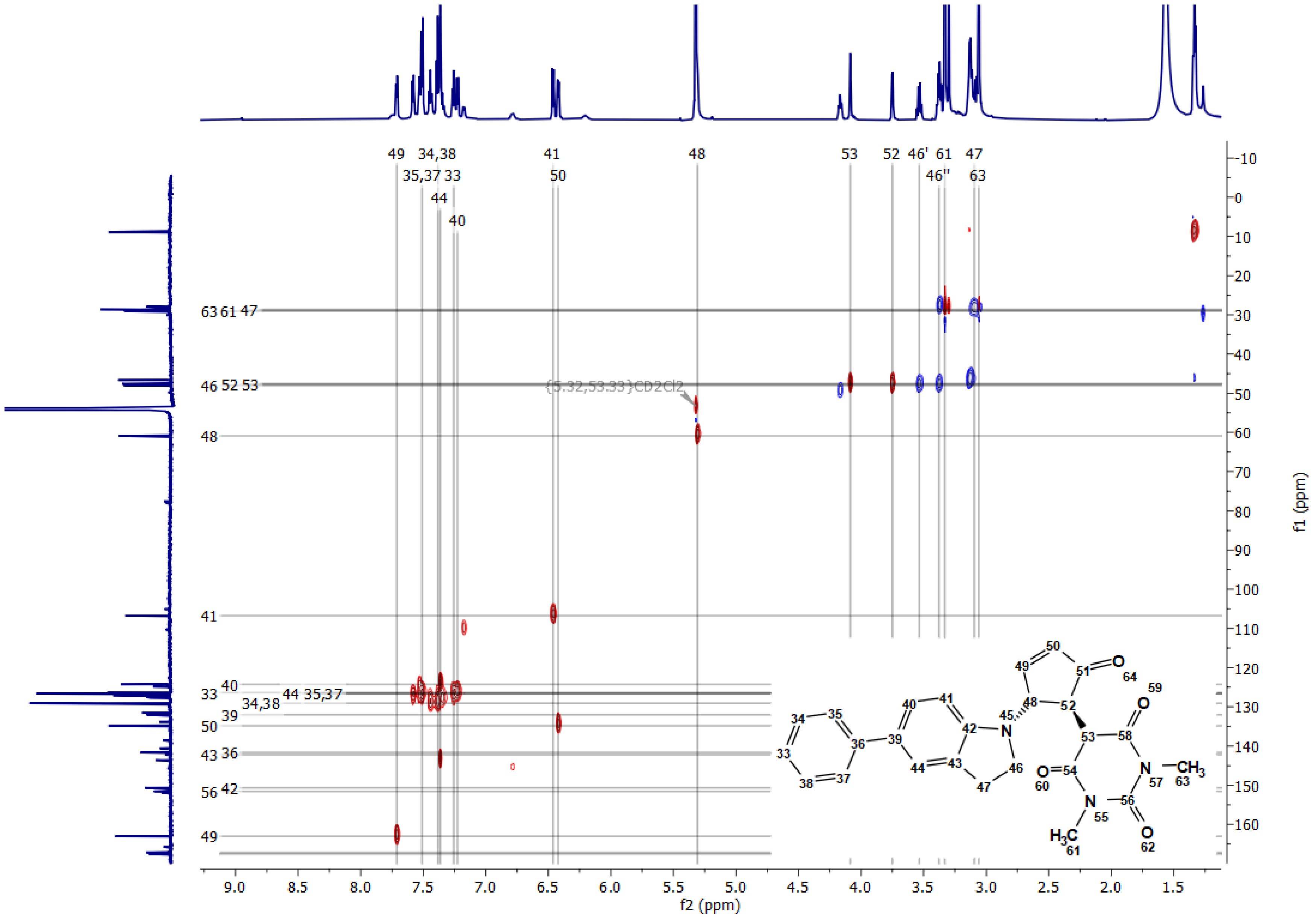

Figure S52. HSQC NMR spectrum of DASA **4** in closed form (700 MHz, CD_2Cl_2, 300 K). For the acquisition of ^{1}H, ^{13}C, COSY, HSQC and HMBC NMR data of DASA **4** in the 700 MHz equipment, right before the acquisition of the spectra, the samples were irradiated at the sample handling robot. This increases drastically the population of the cylopentenone isomers which facilitates the assignments. The minor signals correspond to the open isomer.

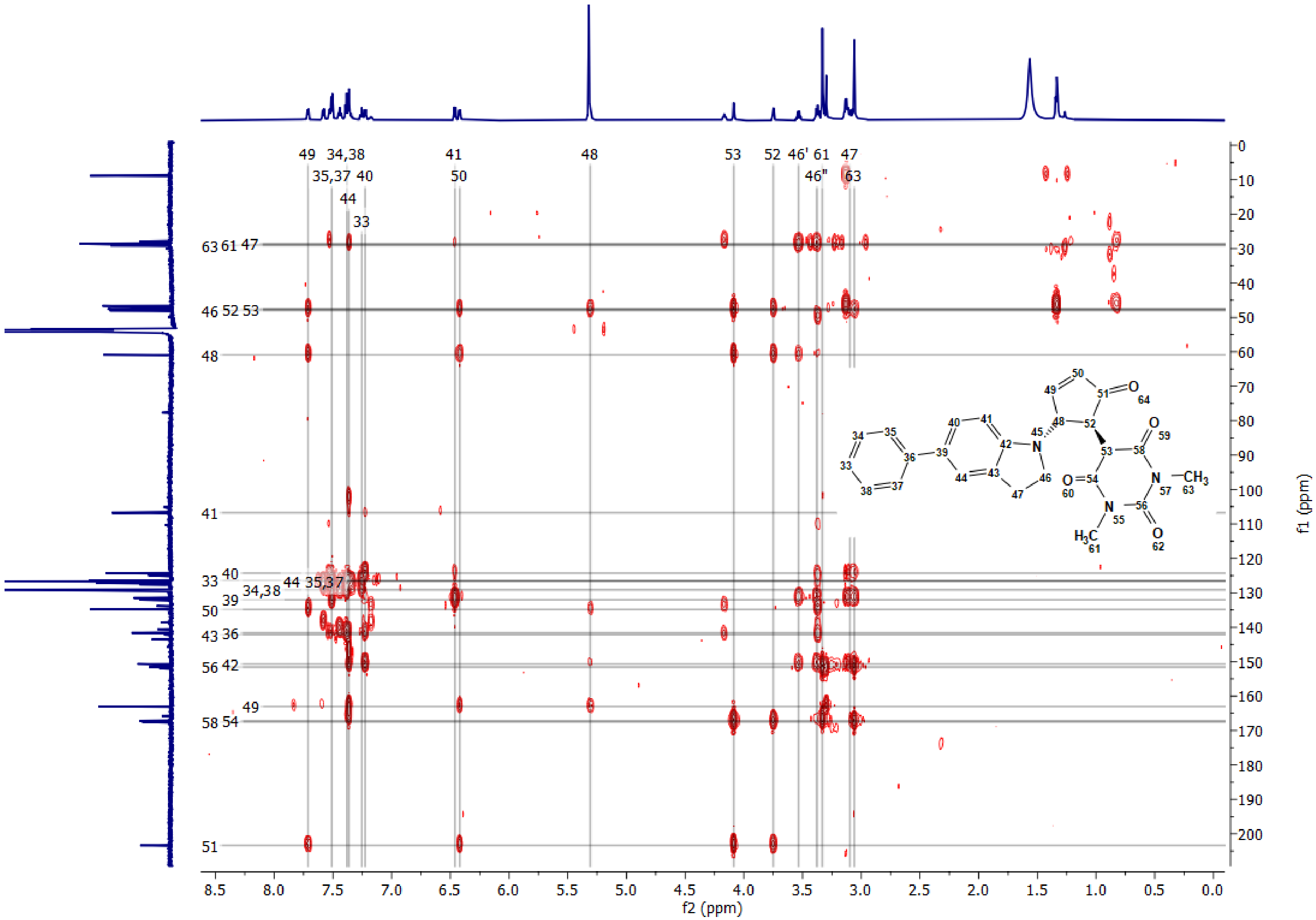

Figure S53. HMBC NMR spectrum of DASA **4** in closed form (700 MHz, CD_2Cl_2, 300 K). For the acquisition of ^1H, ^{13}C, COSY, HSQC and HMBC NMR data of DASA **4** in the 700 MHz equipment, right before the acquisition of the spectra, the samples were irradiated at the sample handling robot. This increases drastically the population of the cylopentenone isomers which facilitates the assignments. The minor signals correspond to the open isomer.

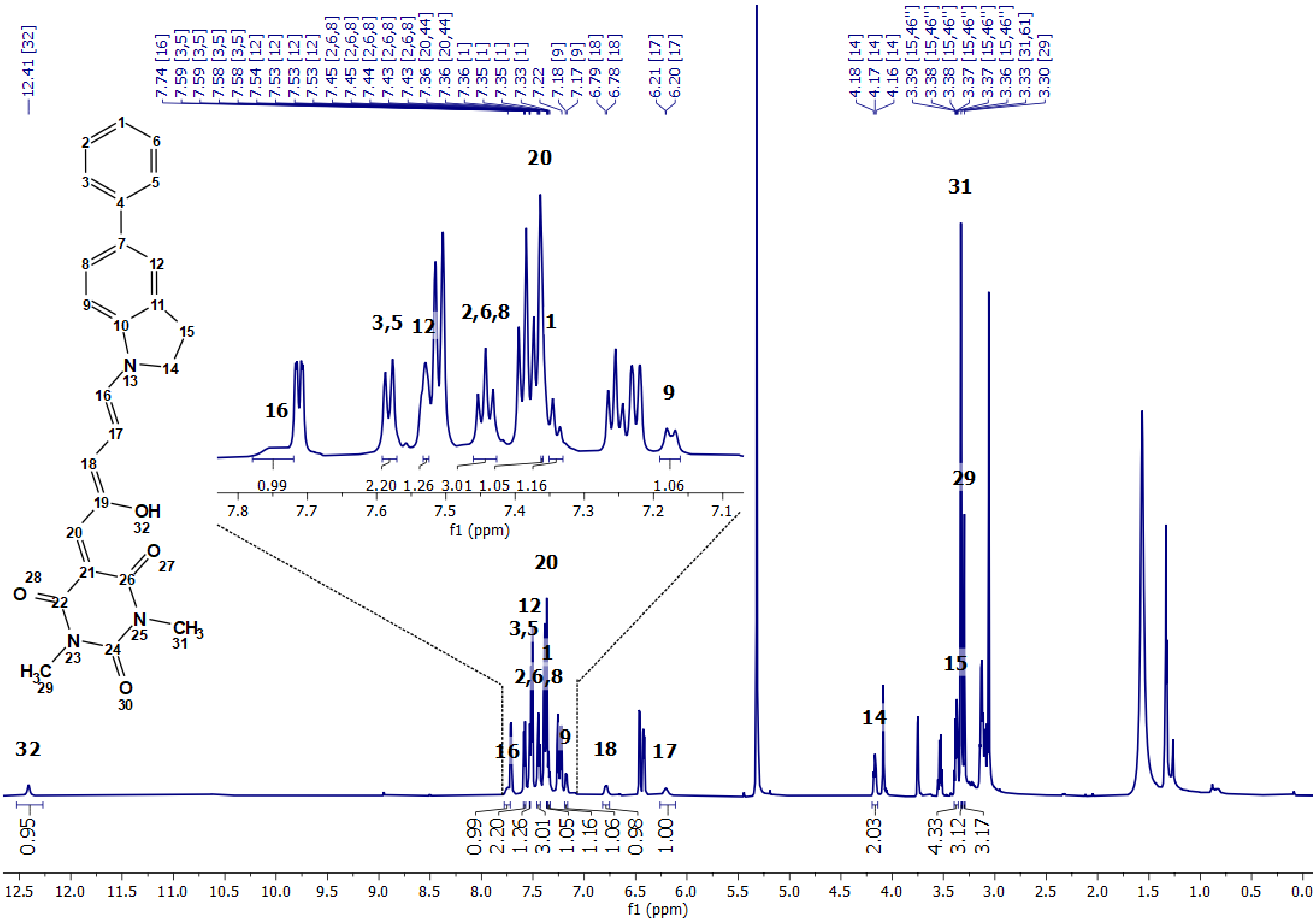

Figure S54. ^{1}H NMR spectrum of DASA **4** in open form (700 MHz, CD_2Cl_2, 300 K). For the acquisition of ^{1}H, ^{13}C, COSY, HSQC and HMBC NMR data of DASA **4** in the 700 MHz equipment, right before the acquisition of the spectra, the samples were irradiated at the sample handling robot. This increases drastically the population of the cylopentenone isomers which facilitates the assignments.

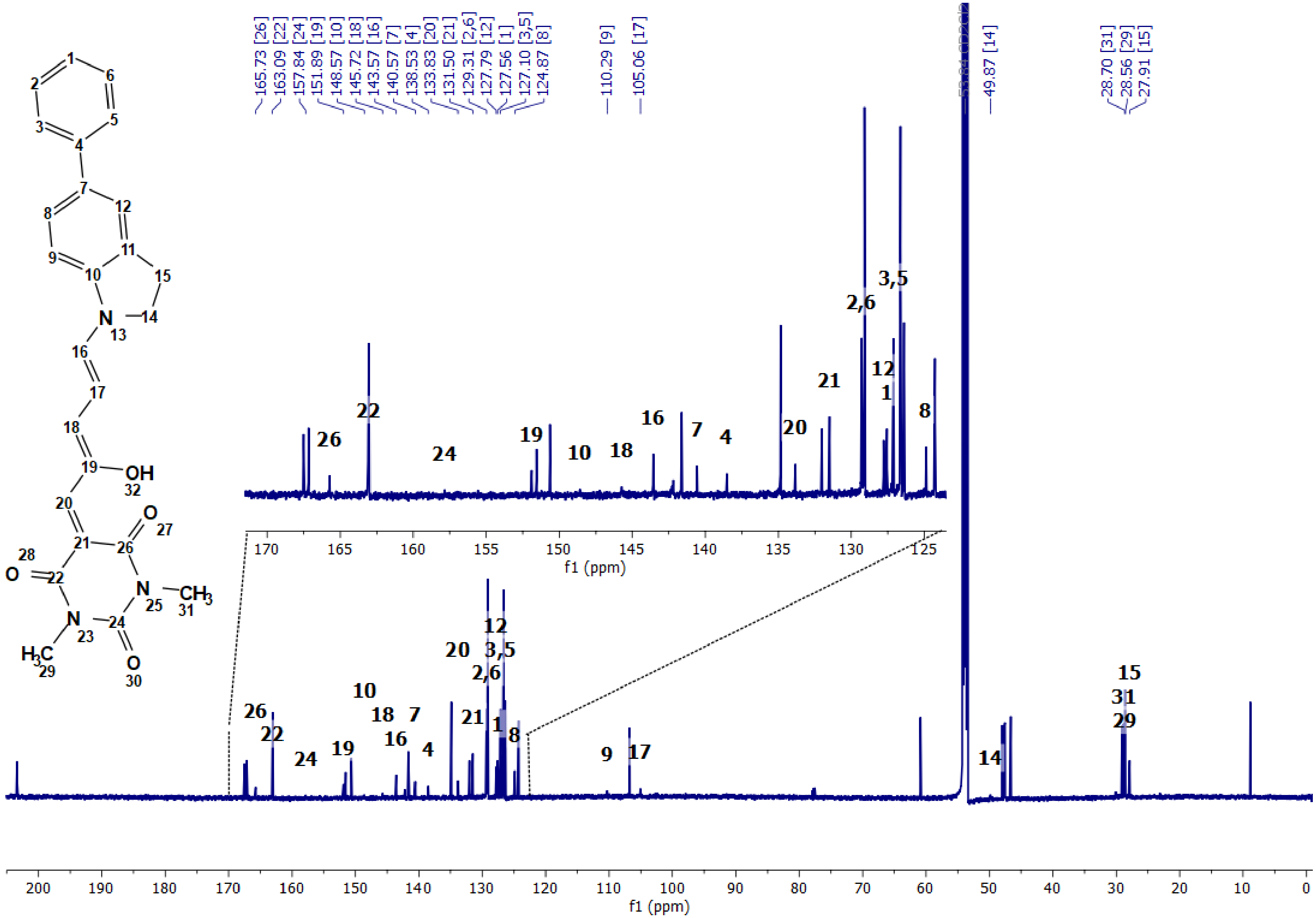

Figure S55. ^{13}C NMR spectrum of DASA **4** in open form (176 MHz, CD_2Cl_2, 300 K). For the acquisition of ^{1}H, ^{13}C, COSY, HSQC and HMBC NMR data of DASA **4** in the 700 MHz equipment, right before the acquisition of the spectra, the samples were irradiated at the sample handling robot. This increases drastically the population of the cylopentenone isomers which facilitates the assignments. The minor signals correspond to the open isomer.

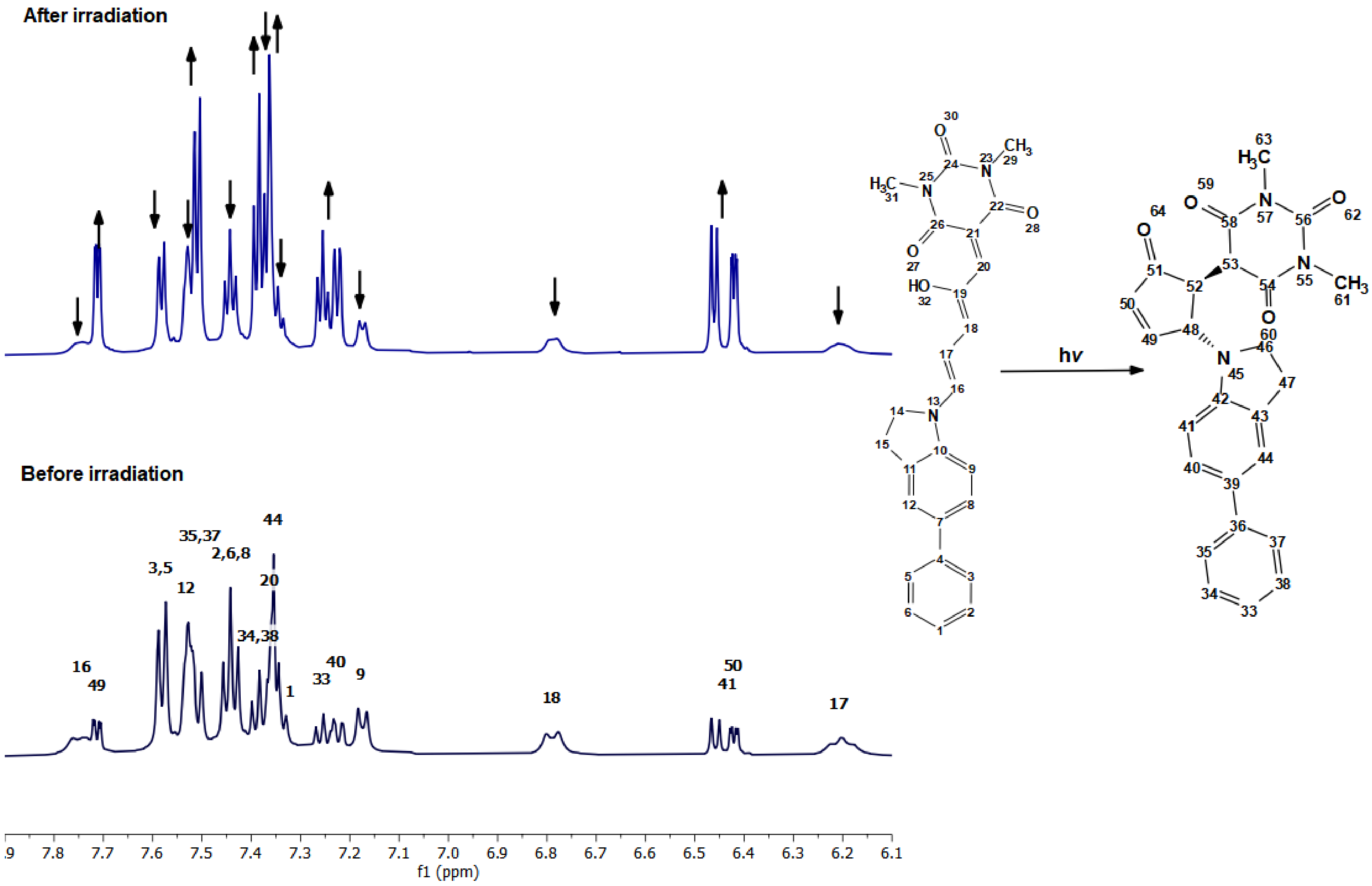

Figure S56. Comparison of ^{1}H NMR spectra of DASA **4** in the aromatic zone. Spectra were acquited in gloom in the 500 MHz equipment (lower, CD_2Cl_2, 300 K) and after irradiation in the 700 MHz equipment (upper, CD_2Cl_2, 300 K). Arrows show either increment and detriment of peaks after irradiation. Right before the acquisition of spectra of 700 MHz, the samples were irradiated at the sample handling robot. This increases drastically the population of the cylopentenone isomers which facilitates the assignments.

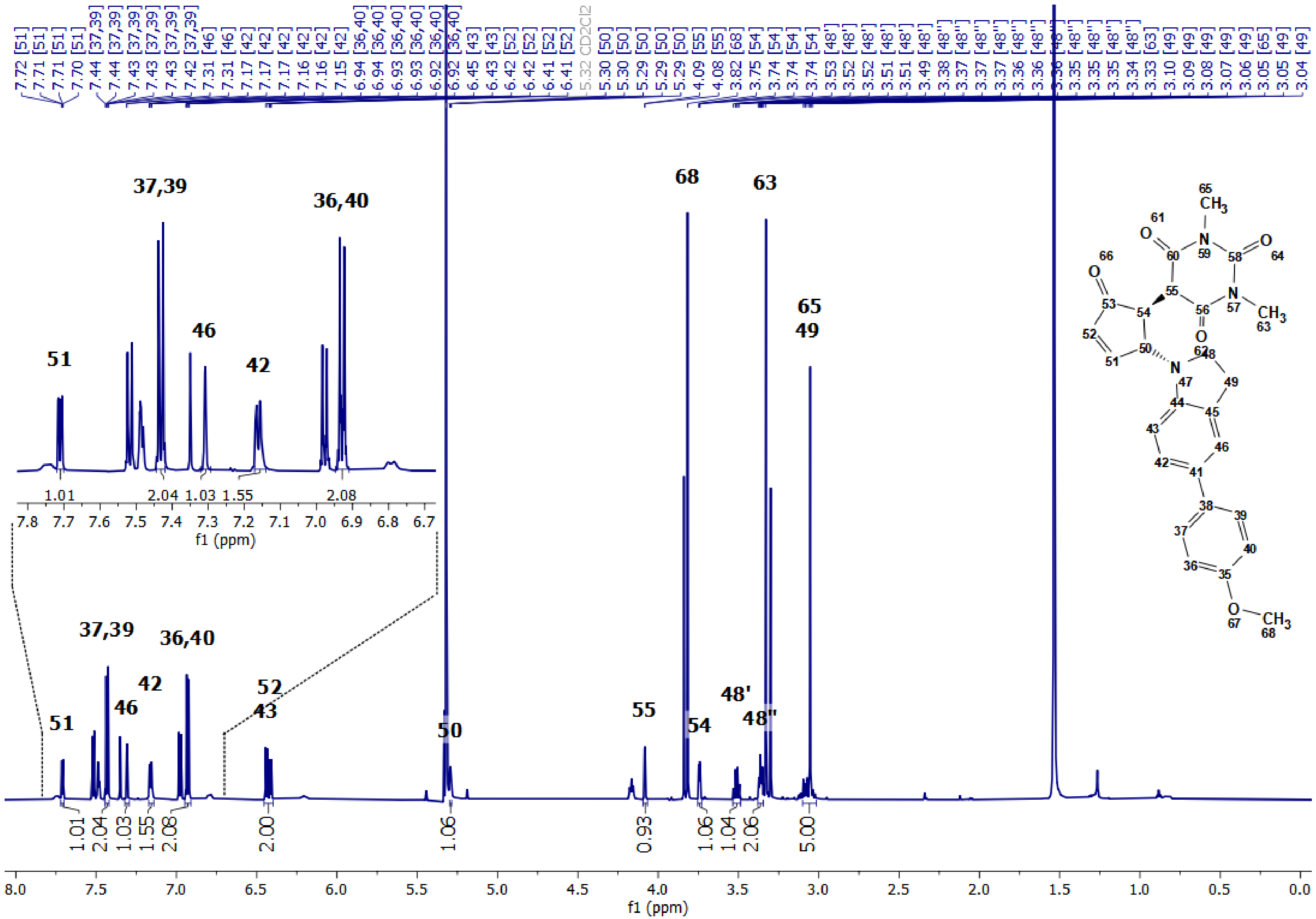

Figure S57. ^{1}H NMR spectrum of DASA **5** in closed form (700 MHz, CD_2Cl_2, 300 K). For the acquisition of ^{1}H and ^{13}C, COSY, HSQC and HMBC NMR data of DASA **5** in the 700 MHz equipment, right before the acquisition of the spectra, the samples were irradiated at the sample handling robot. This increases drastically the population of the cylopentenone isomers which facilitates the assignments. The minor signals correspond to the open isomer.

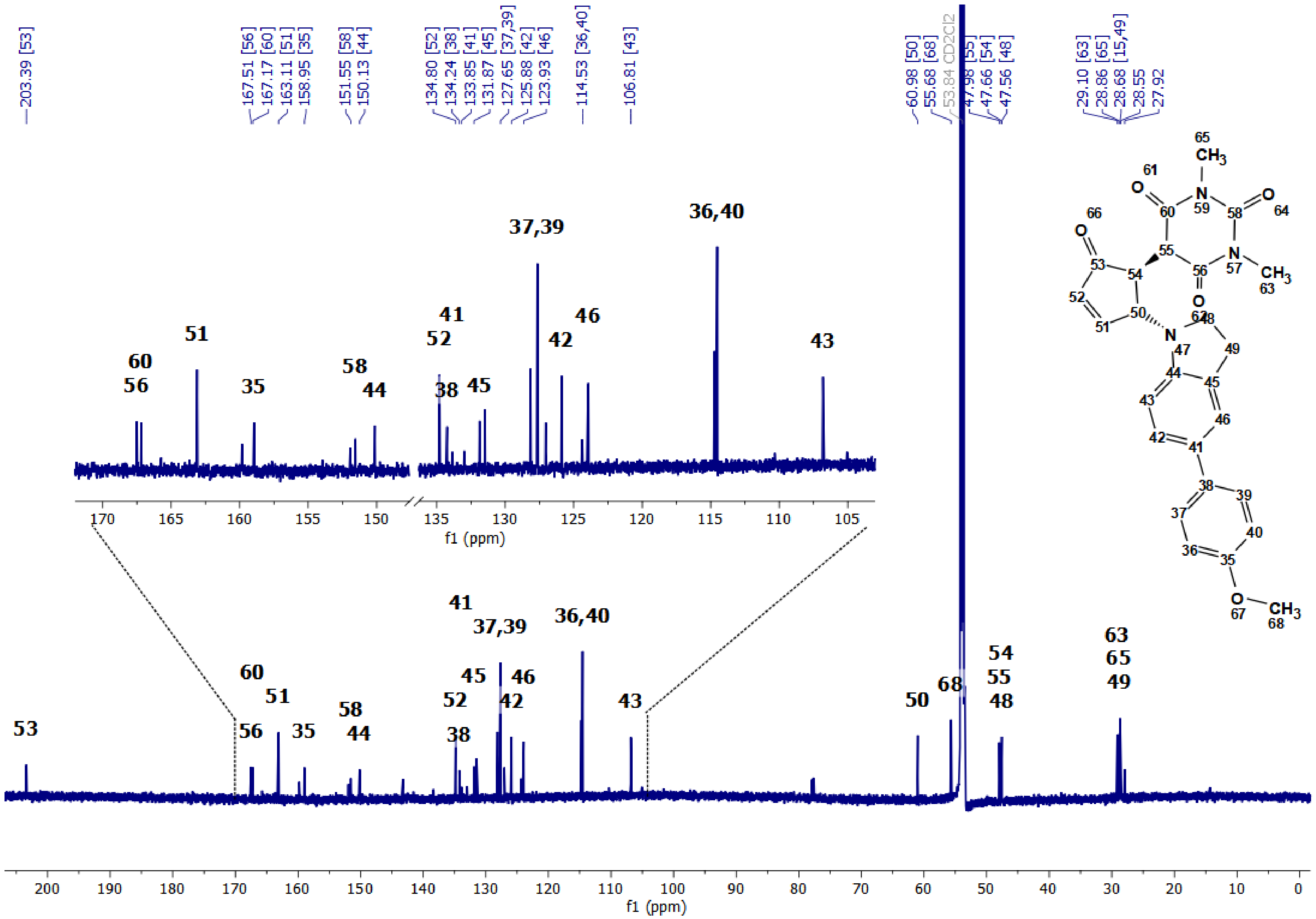

Figure S58. ^{13}C NMR spectrum of DASA **5** in closed form (176 MHz, CD_2Cl_2, 300 K). For the acquisition of ^{1}H, ^{13}C, COSY, HSQC and HMBC NMR data of DASA **5** in the 700 MHz equipment, right before the acquisition of the spectra, the samples were irradiated at the sample handling robot. This increases drastically the population of the cylopentenone isomers which facilitates the assignments. The minor signals correspond to the open isomer.

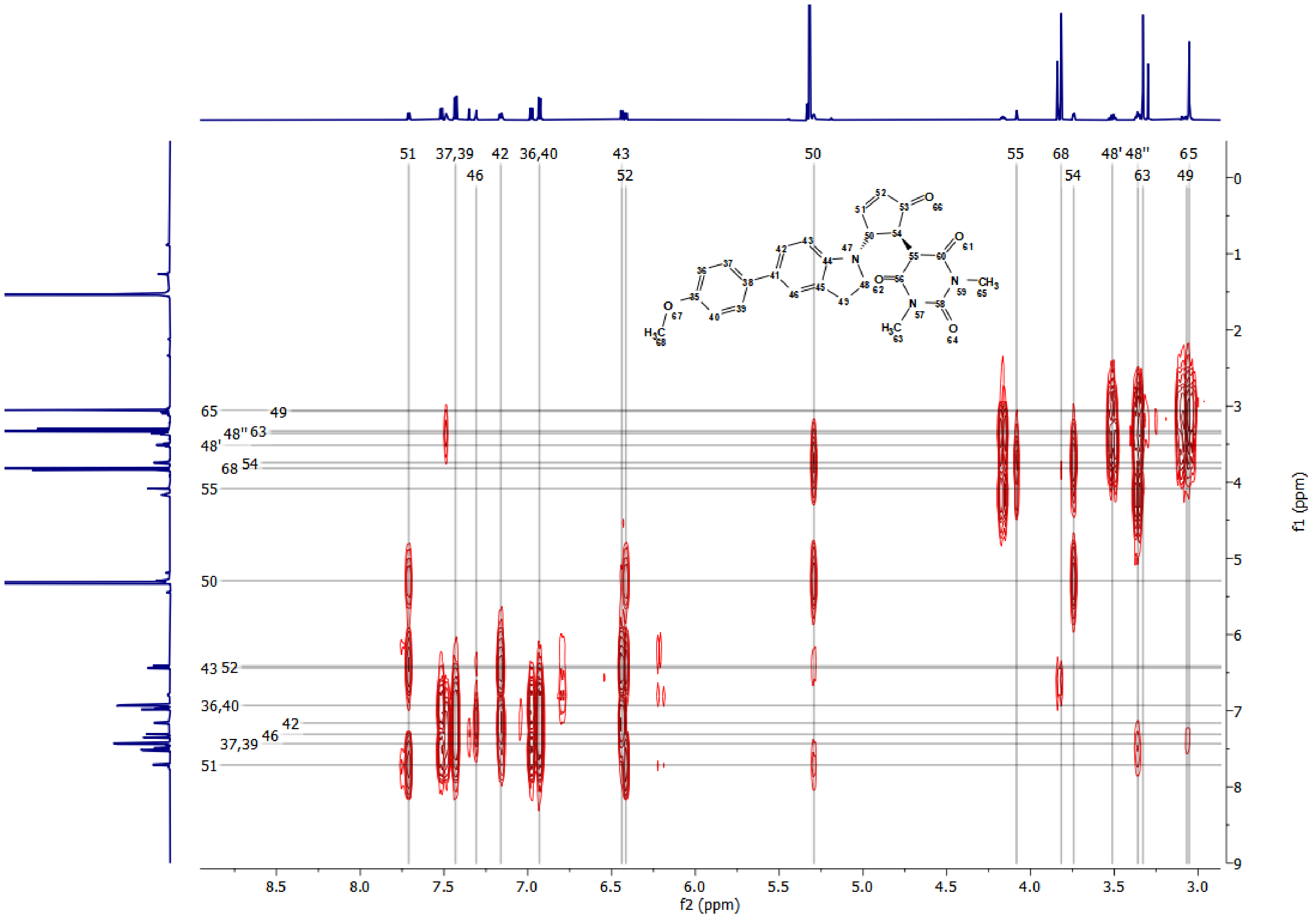

Figure S59. COSY NMR spectrum of DASA **5** in closed form (700 MHz, CD_2Cl_2, 300 K). For the acquisition of ^{1}H, ^{13}C, COSY, HSQC and HMBC NMR data of DASA **5** in the 700 MHz equipment, right before the acquisition of the spectra, the samples were irradiated at the sample handling robot. This increases drastically the population of the cylopentenone isomers which facilitates the assignments. The minor signals correspond to the open isomer.

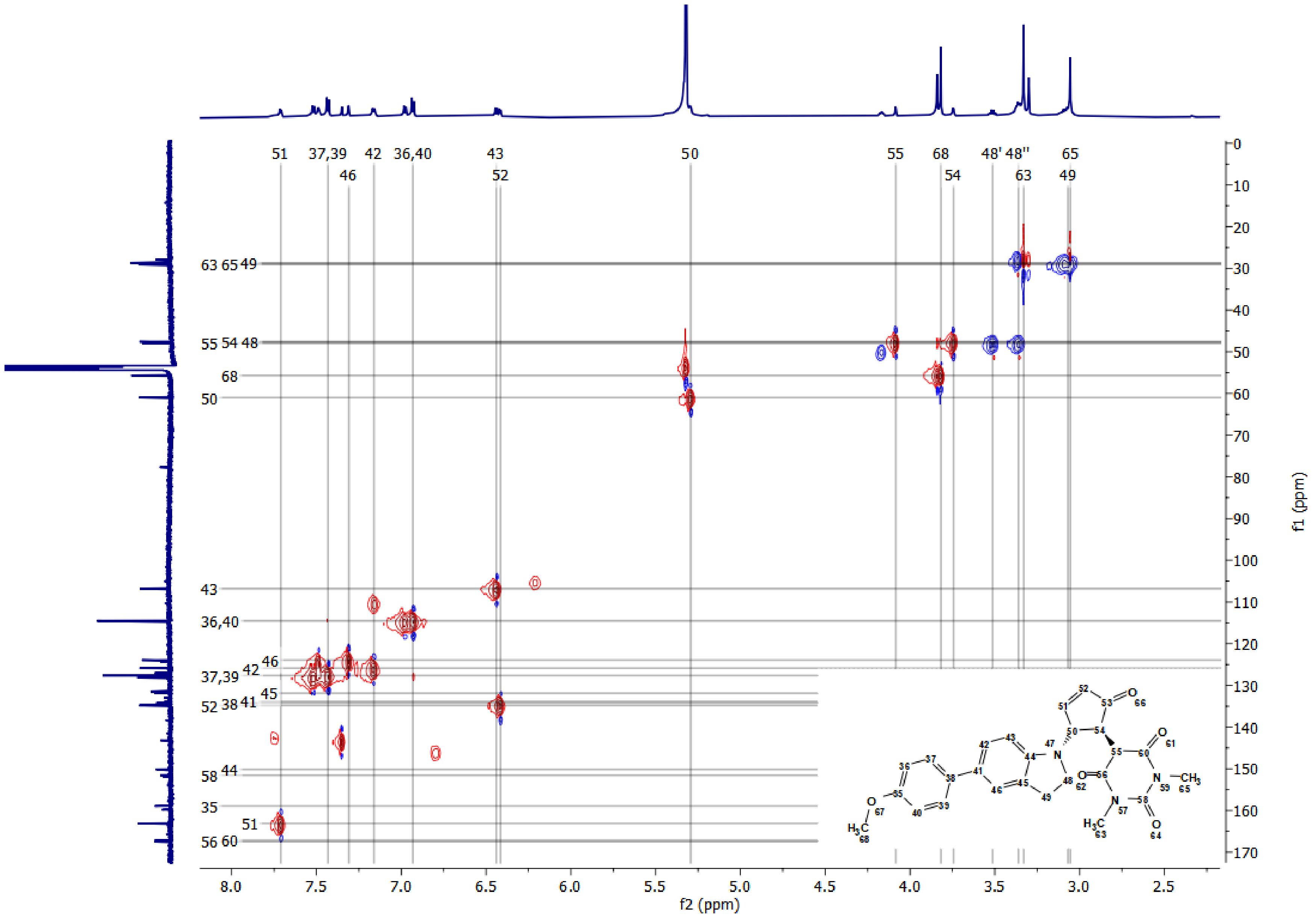

Figure S60. HSQC NMR spectrum of DASA **5** in closed form (700 MHz, CD_2Cl_2, 300 K). For the acquisition of ^1H, ^{13}C, COSY, HSQC and HMBC NMR data of DASA **5** in the 700 MHz equipment, right before the acquisition of the spectra, the samples were irradiated at the sample handling robot. This increases drastically the population of the cylopentenone isomers which facilitates the assignments. The minor signals correspond to the open isomer.

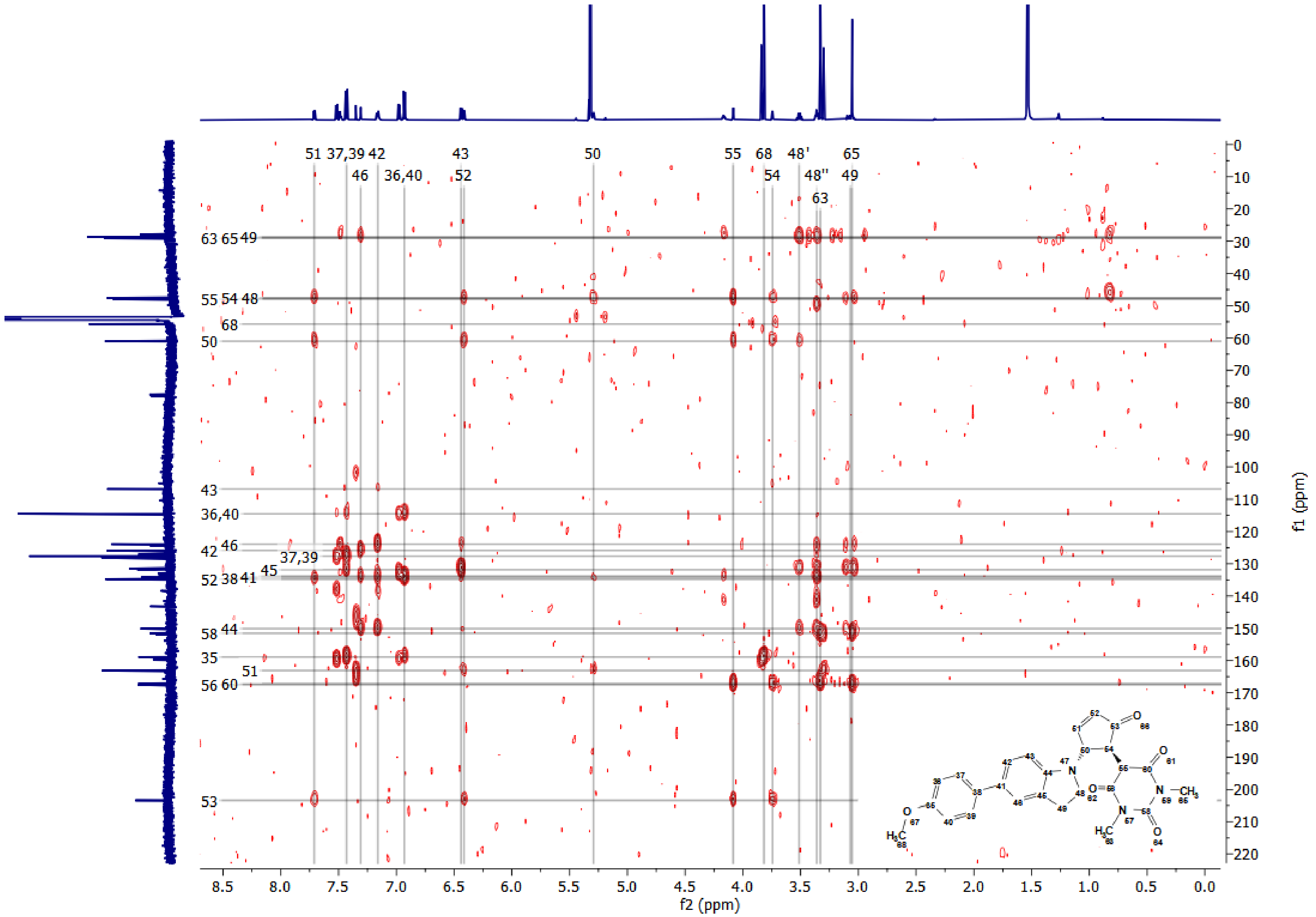

Figure S61. HMBC NMR spectrum of DASA **5** in closed form (700 MHz, CD_2Cl_2, 300 K). For the acquisition of ^{1}H, ^{13}C, COSY, HSQC and HMBC NMR data of DASA **5** in the 700 MHz equipment, right before the acquisition of the spectra, the samples were irradiated at the sample handling robot. This increases drastically the population of the cylopentenone isomers which facilitates the assignments. The minor signals correspond to the open isomer.

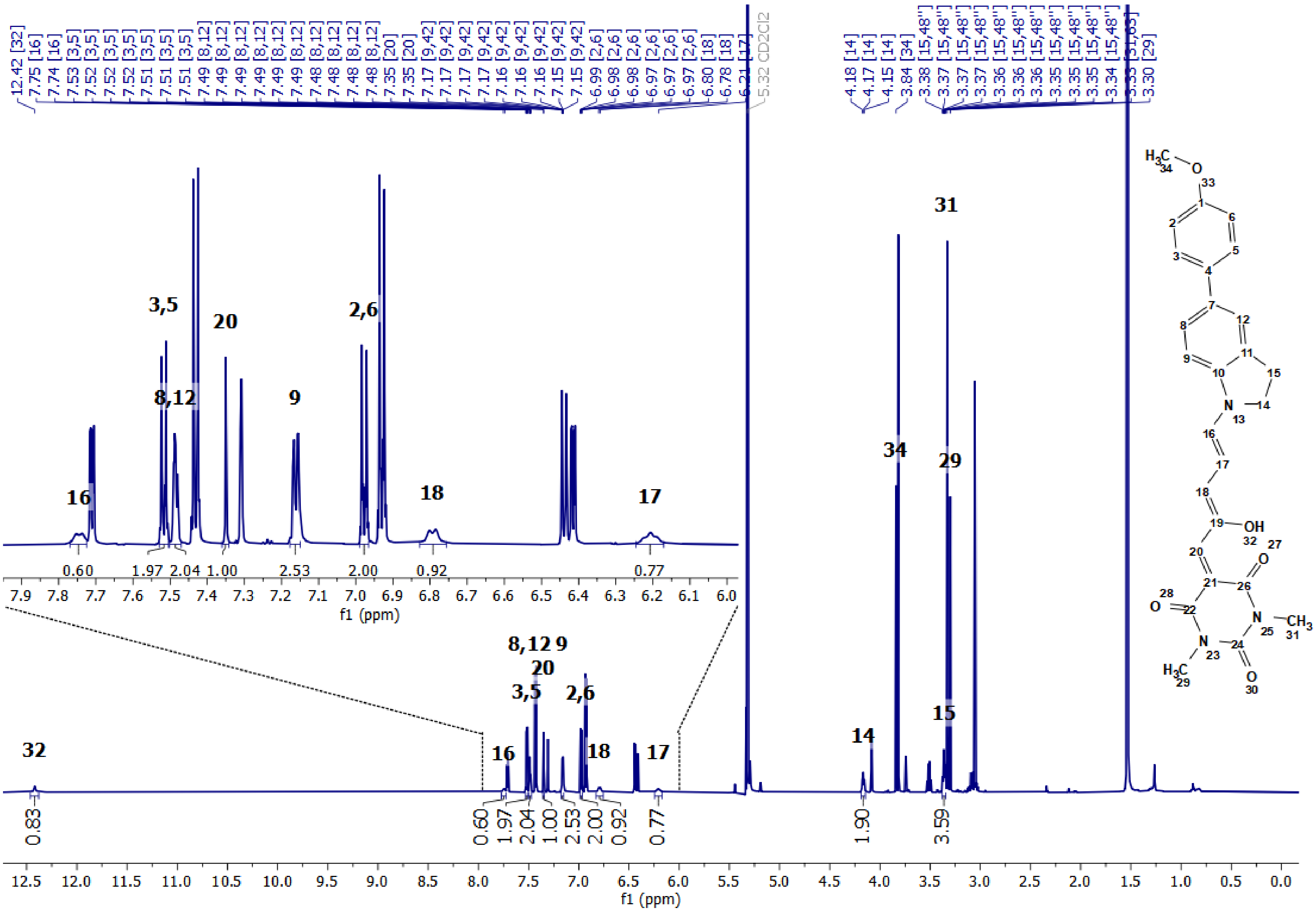

Figure S62. ^{1}H NMR spectrum of DASA **5** in open form (700 MHz, CD_2Cl_2, 300 K). For the acquisition of ^{1}H, ^{13}C, COSY, HSQC and HMBC NMR data of DASA **5** in the 700 MHz equipment, right before the acquisition of the spectra, the samples were irradiated at the sample handling robot. This increases drastically the population of the cylopentenone isomers which facilitates the assignments.

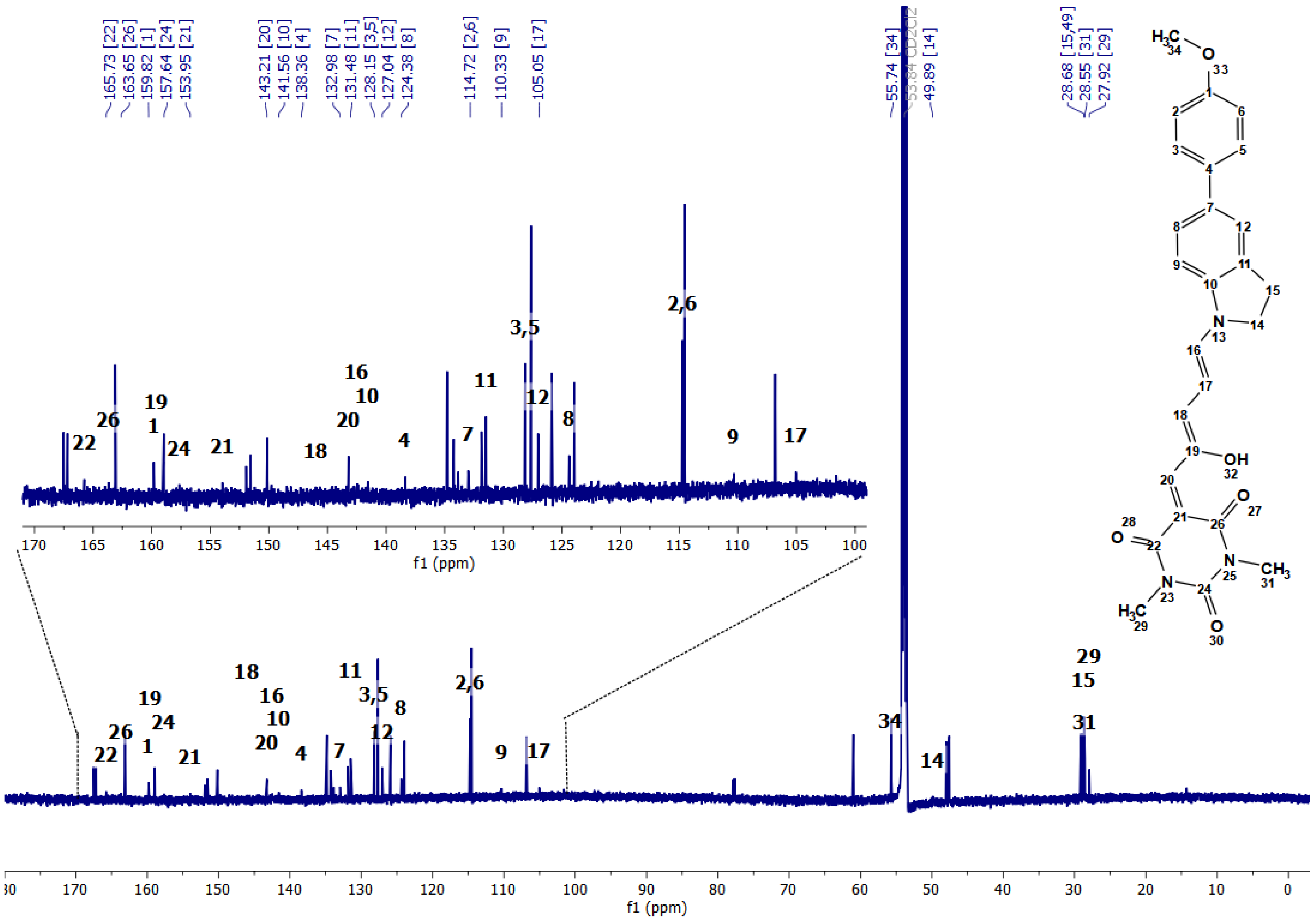

Figure S63. ^{13}C NMR spectrum of DASA **5** in open form (176 MHz, CD_2Cl_2, 300 K). For the acquisition of ^{1}H, ^{13}C, COSY, HSQC and HMBC NMR data of DASA **5** in the 700 MHz equipment, right before the acquisition of the spectra, the samples were irradiated at the sample handling robot. This increases drastically the population of the cylopentenone isomers which facilitates the assignments. The minor signals correspond to the open isomer.

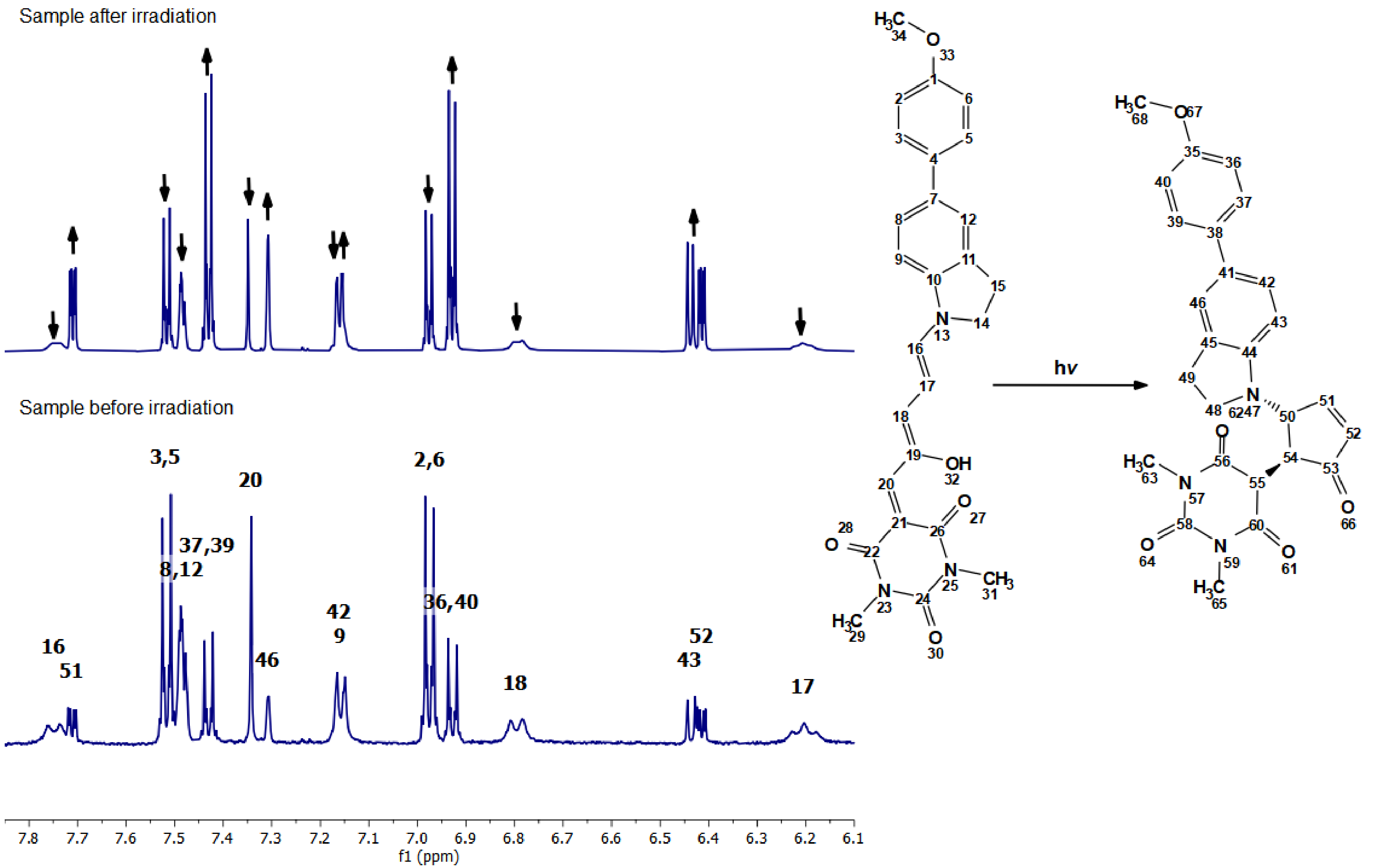

Figure S64. Comparison of ^{1}H NMR spectra of DASA **5** in the aromatic zone. Spectra were acquited in gloom in the 500 MHz equipment (lower, CD_2Cl_2, 300 K) and after irradiation in the 700 MHz equipment (upper, CD_2Cl_2, 300 K). Arrows show either increment and detriment of peaks after irradiation. Right before the acquisition of spectra of 700 MHz, the samples were irradiated at the sample handling robot. This increases drastically the population of the cylopentenone isomers which facilitates the assignments.

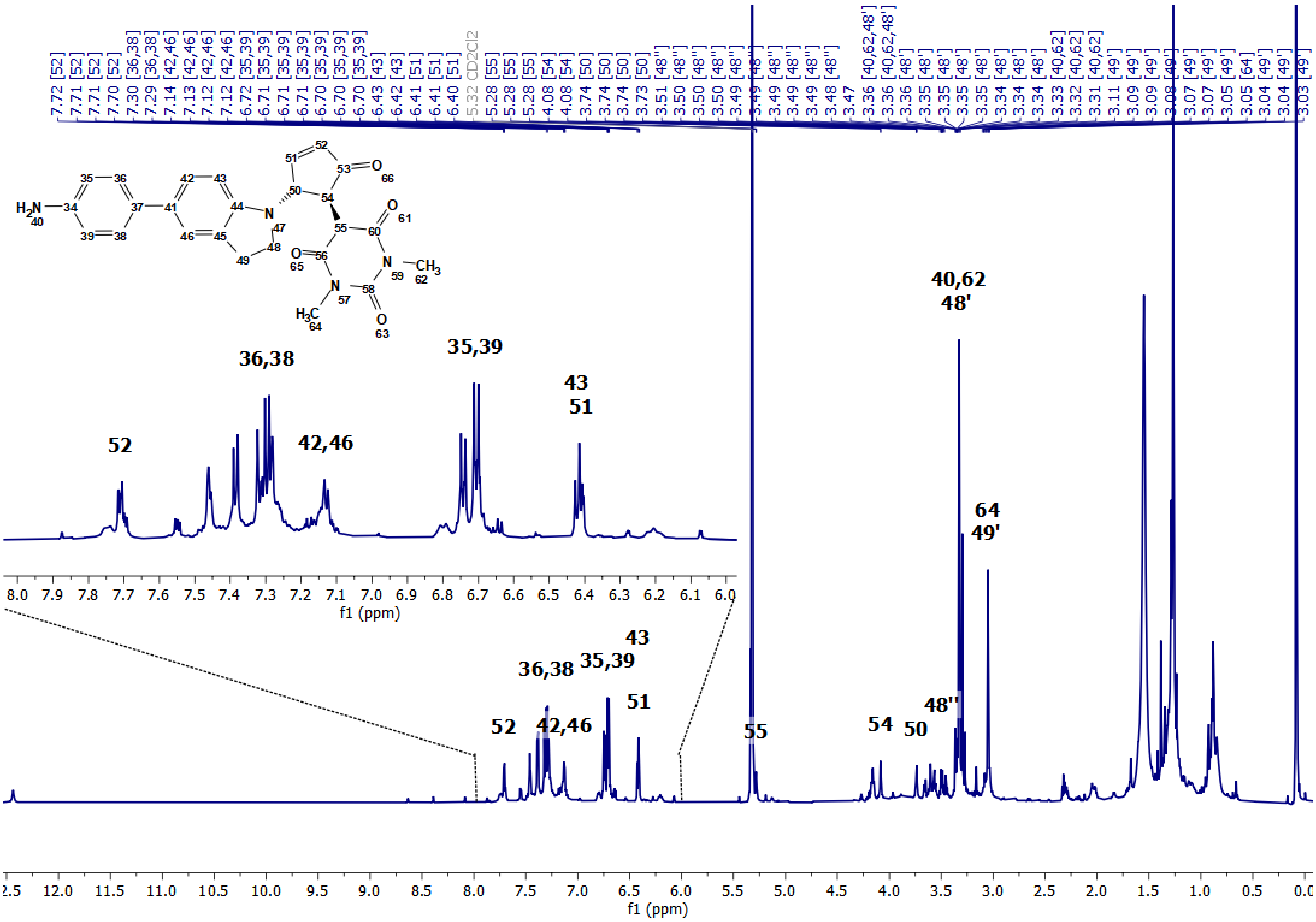

Figure S65. ^{1}H NMR spectrum of DASA **6** in closed form (700 MHz, CD_2Cl_2, 300 K). For the acquisition of ^{1}H and ^{13}C, COSY, HSQC and HMBC NMR data of DASA **6** in the 700 MHz equipment, right before the acquisition of the spectra, the samples were irradiated at the sample handling robot. This increases drastically the population of the cylopentenone isomers which facilitates the assignments. The minor signals correspond to the open isomer.

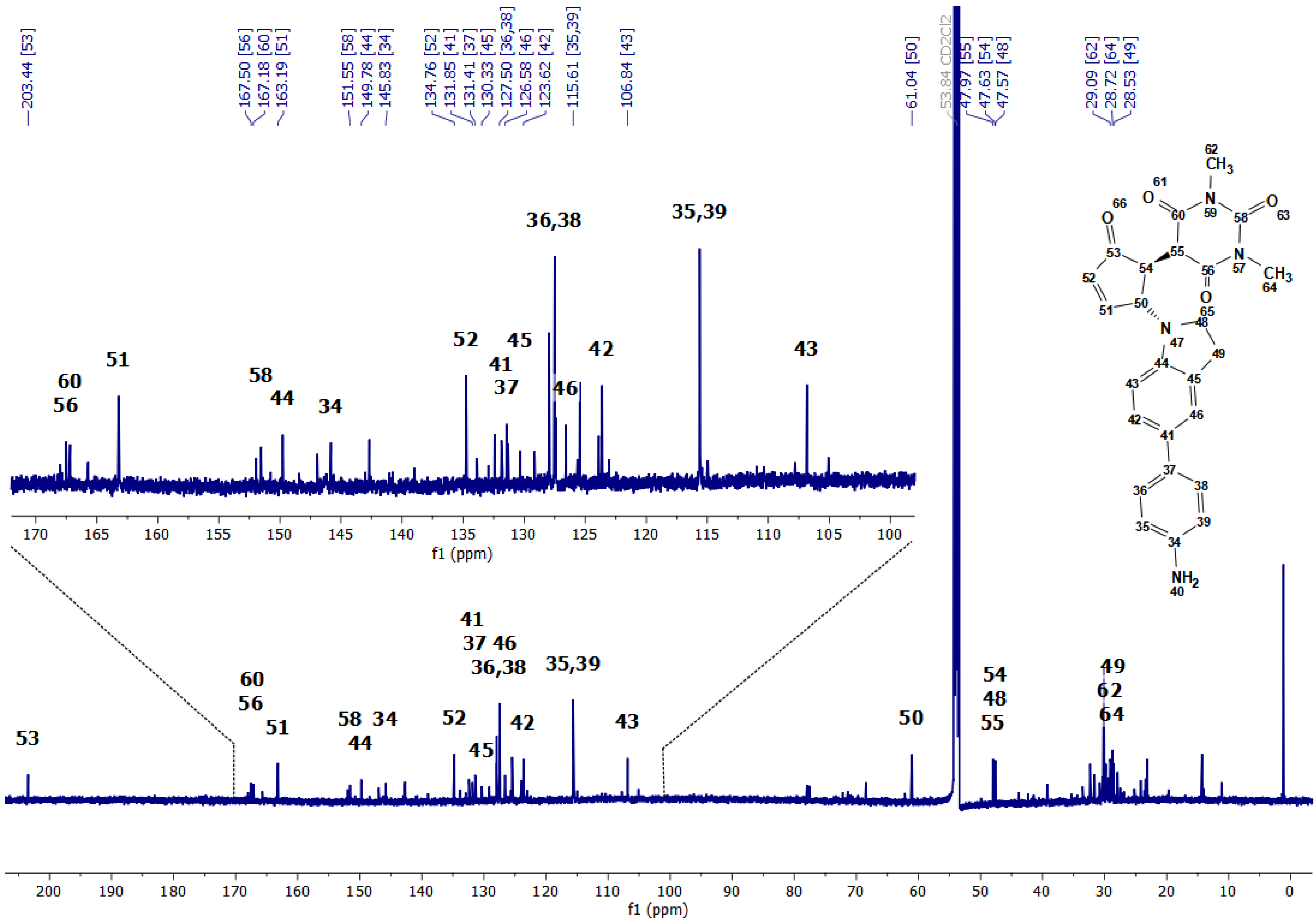

Figure S66. ^{13}C NMR spectrum of DASA **6** in closed form (176 MHz, CD_2Cl_2, 300 K). For the acquisition of ^{1}H, ^{13}C, COSY, HSQC and HMBC NMR data of DASA **6** in the 700 MHz equipment, right before the acquisition of the spectra, the samples were irradiated at the sample handling robot. This increases drastically the population of the cylopentenone isomers which facilitates the assignments. The minor signals correspond to the open isomer.

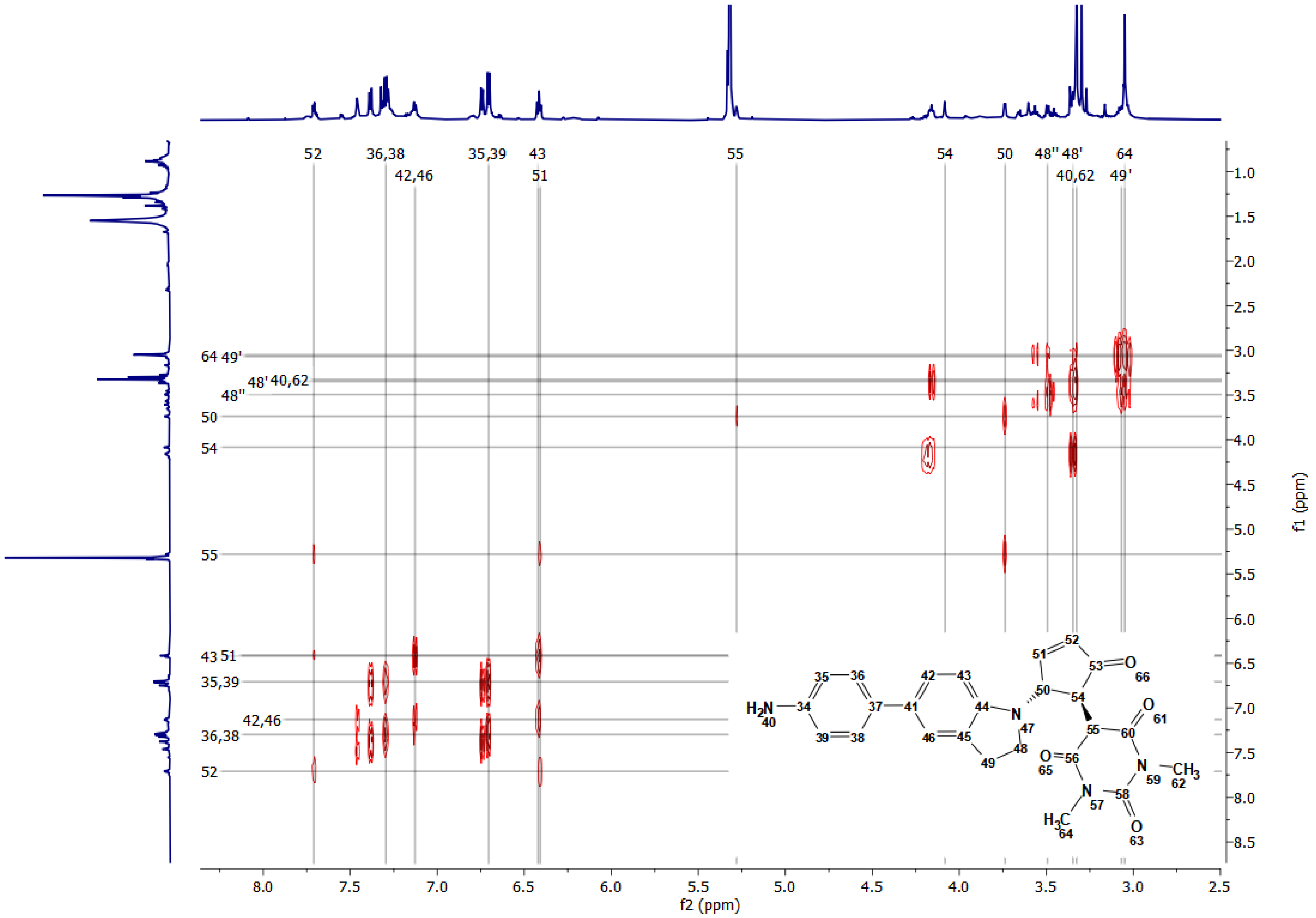

Figure S67. COSY NMR spectrum of DASA **6** in closed form (700 MHz, CD_2Cl_2, 300 K). For the acquisition of ^{1}H, ^{13}C, COSY, HSQC and HMBC NMR data of DASA **6** in the 700 MHz equipment, right before the acquisition of the spectra, the samples were irradiated at the sample handling robot. This increases drastically the population of the cylopentenone isomers which facilitates the assignments. The minor signals correspond to the open isomer.

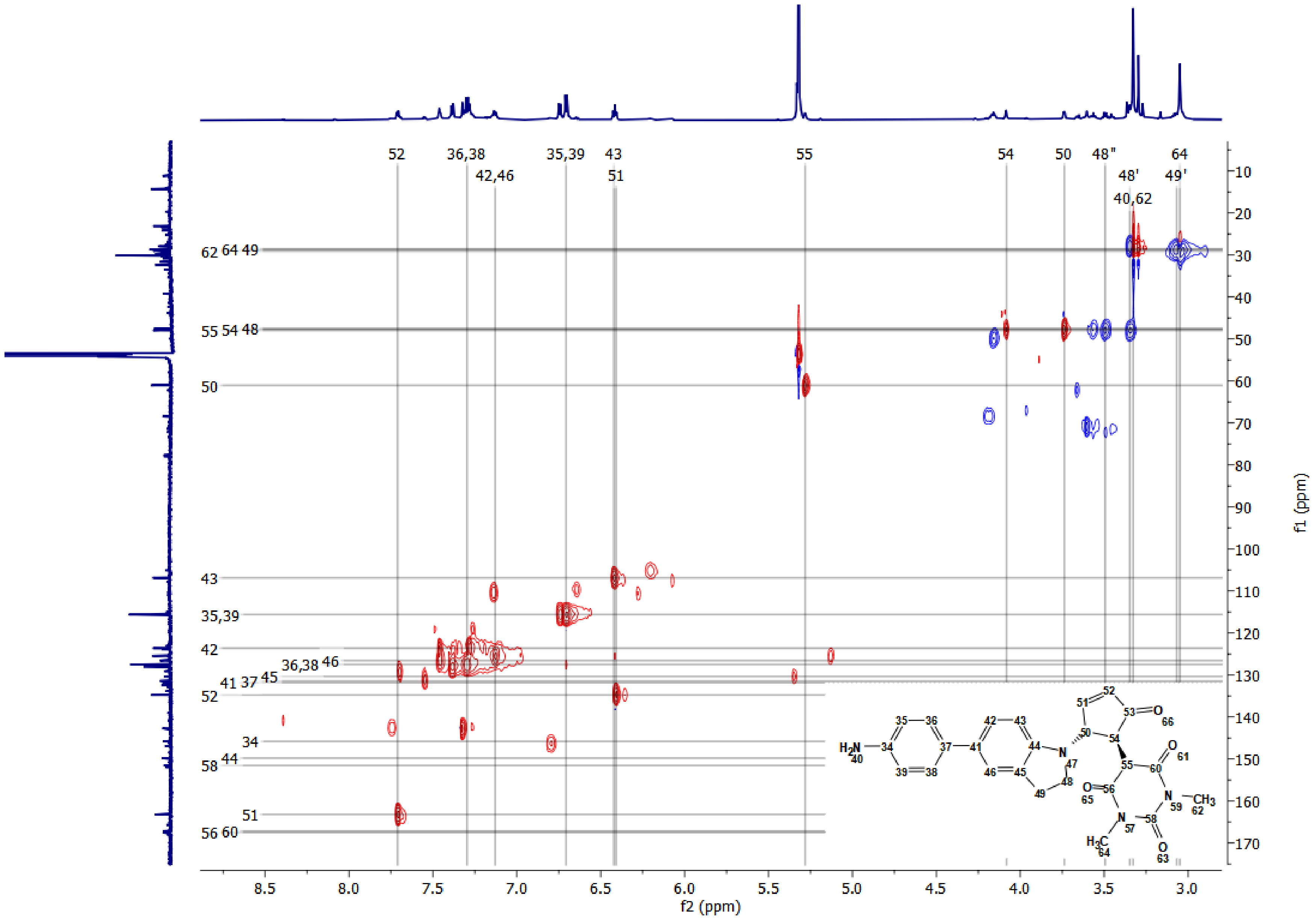

Figure S68. HSQC NMR spectrum of DASA **6** in closed form (700 MHz, CD_2Cl_2, 300 K). For the acquisition of ^{1}H, ^{13}C, COSY, HSQC and HMBC NMR data of DASA **6** in the 700 MHz equipment, right before the acquisition of the spectra, the samples were irradiated at the sample handling robot. This increases drastically the population of the cylopentenone isomers which facilitates the assignments. The minor signals correspond to the open isomer.

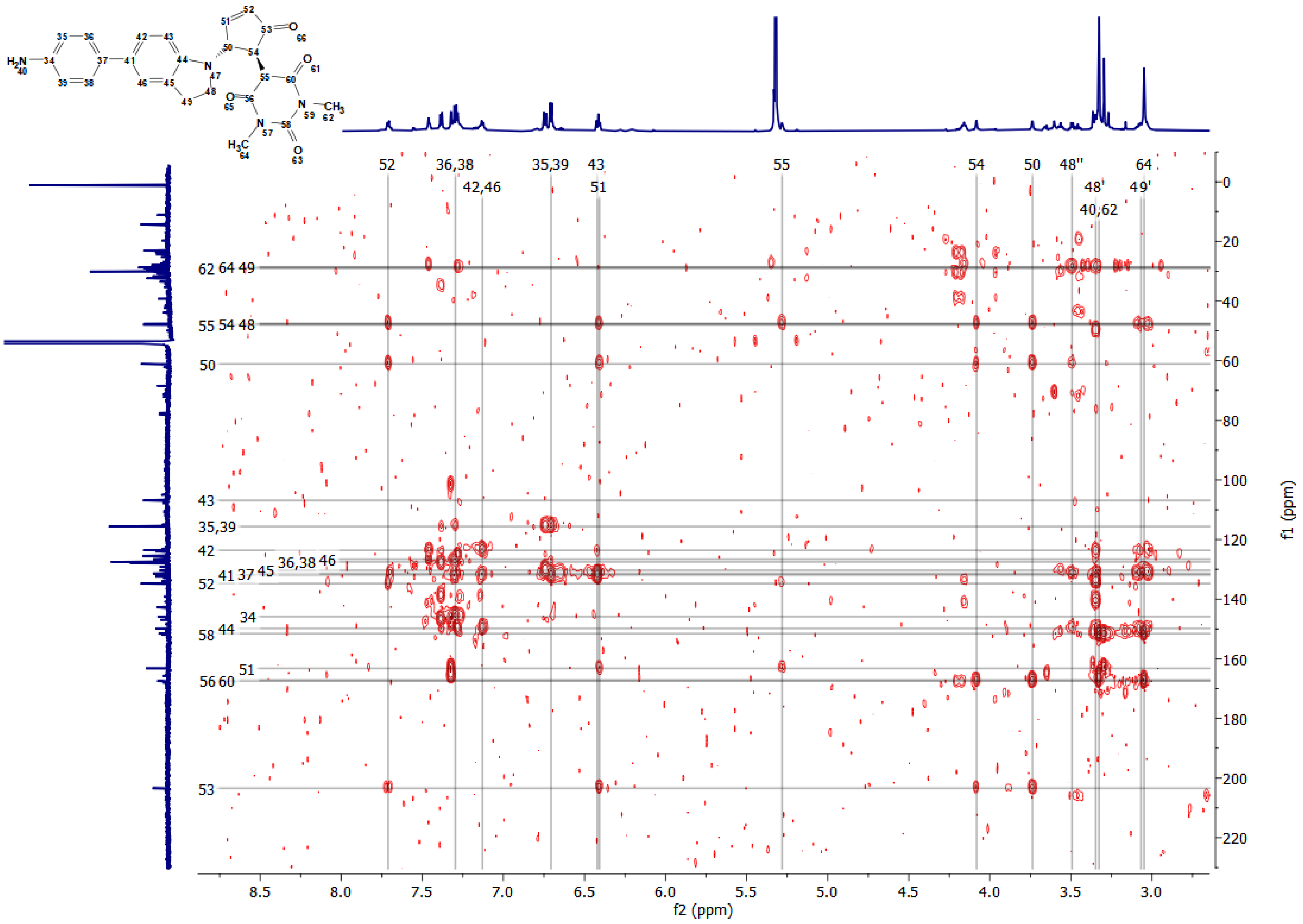

Figure S69. HMBC NMR spectrum of DASA **6** in closed form (700 MHz, CD_2Cl_2, 300 K). For the acquisition of ^{1}H, ^{13}C, COSY, HSQC and HMBC NMR data of DASA **6** in the 700 MHz equipment, right before the acquisition of the spectra, the samples were irradiated at the sample handling robot. This increases drastically the population of the cylopentenone isomers which facilitates the assignments. The minor signals correspond to the open isomer.

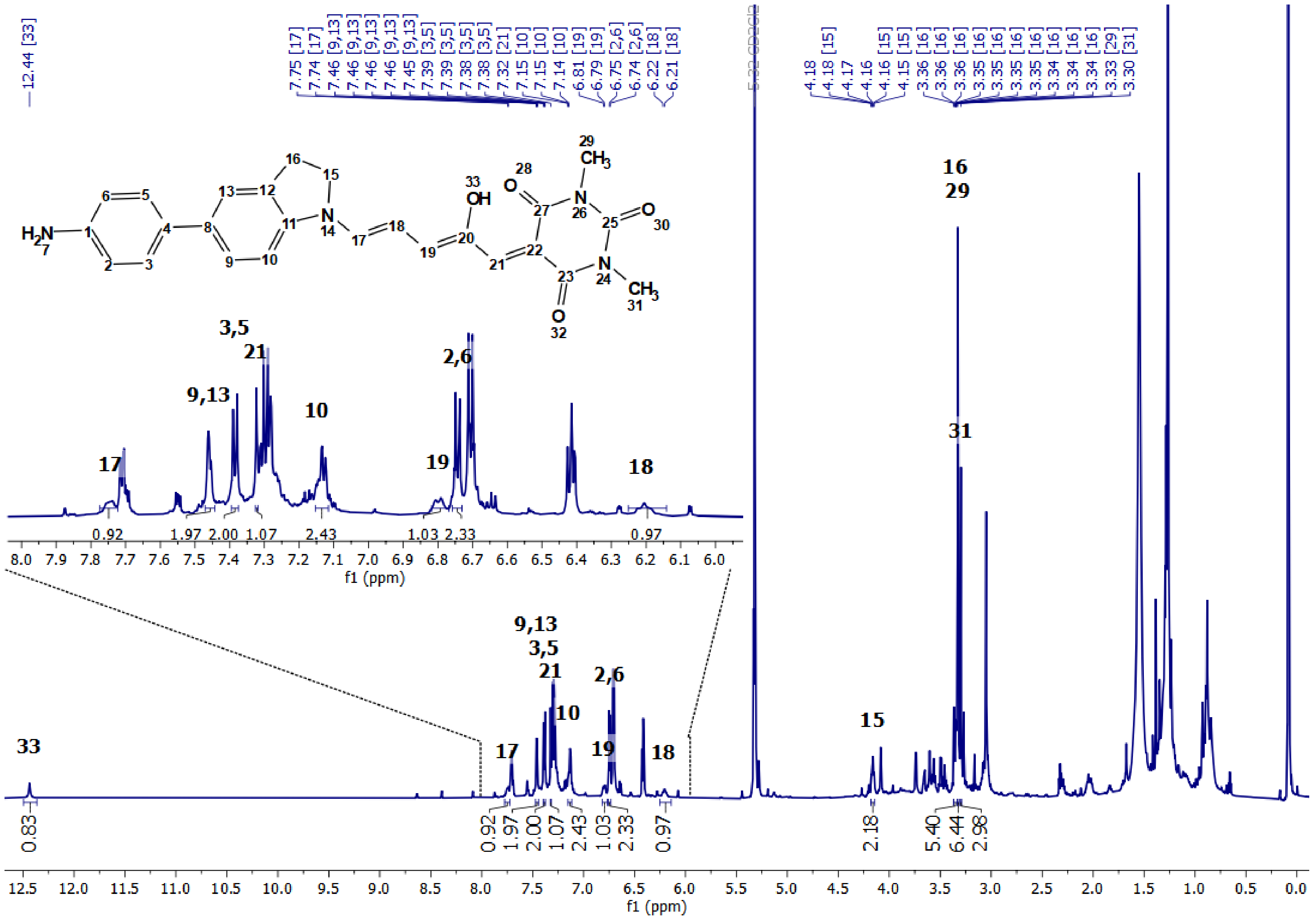

Figure S70. ^{1}H NMR spectrum of DASA **6** in open form (700 MHz, CD_2Cl_2, 300 K). For the acquisition of ^{1}H, ^{13}C, COSY, HSQC and HMBC NMR data of DASA **6** in the 700 MHz equipment, right before the acquisition of the spectra, the samples were irradiated at the sample handling robot. This increases drastically the population of the cylopentenone isomers which facilitates the assignments.

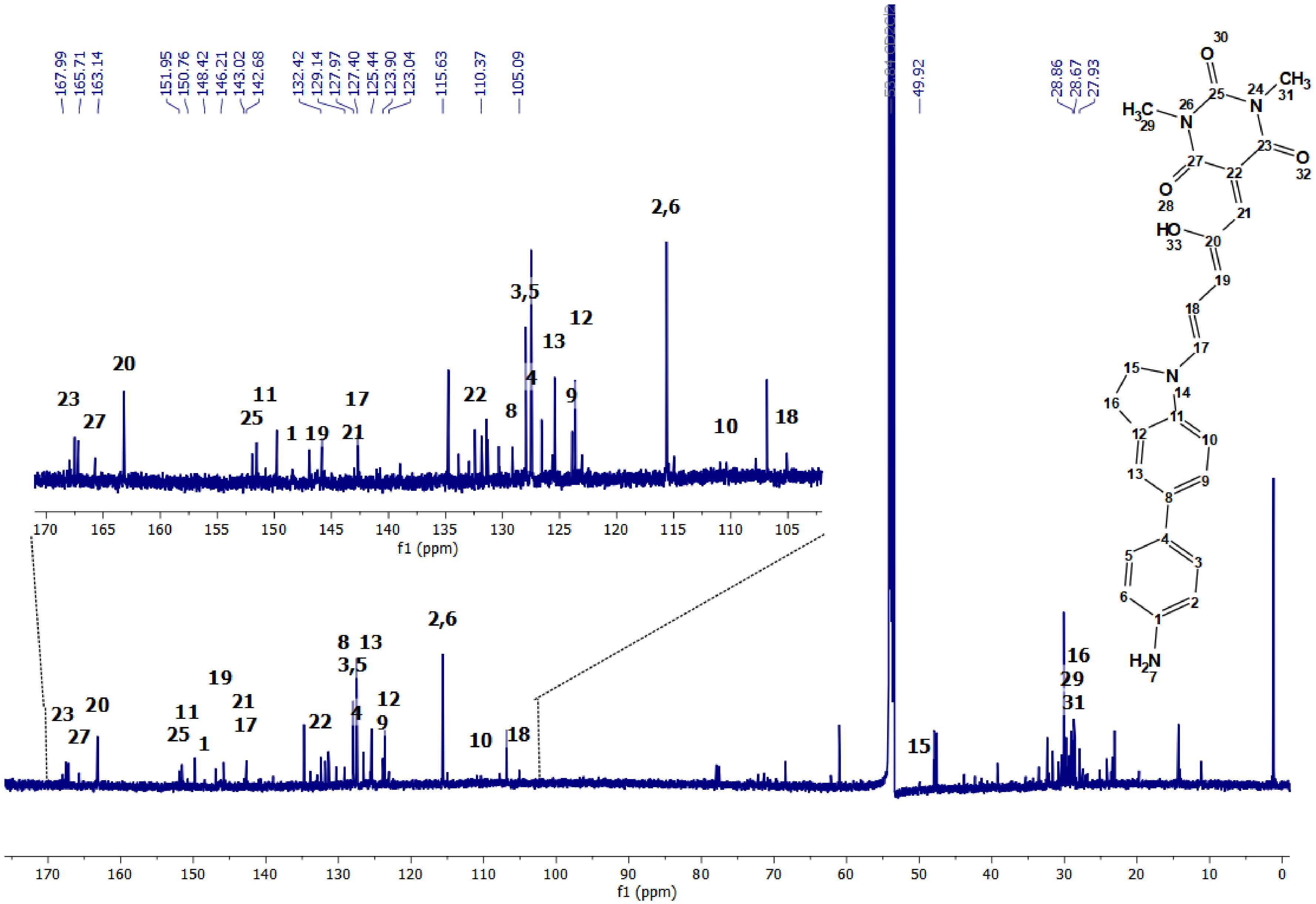

Figure S71. ^{13}C NMR spectrum of DASA **6** in open form (176 MHz, CD_2Cl_2, 300 K). For the acquisition of ^{1}H, ^{13}C, COSY, HSQC and HMBC NMR data of DASA **6** in the 700 MHz equipment, right before the acquisition of the spectra, the samples were irradiated at the sample handling robot. This increases drastically the population of the cylopentenone isomers which facilitates the assignments. The minor signals correspond to the open isomer.

Reference

(1) Hemmer, J. R.; Poelma, S. O.; Treat, N.; Page, Z. A.; Dolinski, N. D.; Diaz, Y. J.; Tomlinson, W.; Clark, K. D.; Hooper, J. P.; Hawker, C.; Read de Alaniz, J. *J. Am. Chem. Soc.* **2016**, *138* (42), 13960.
